# Extensive lineage-specific gene duplication and evolution of the spiggin multi-gene family in stickleback

**DOI:** 10.1186/1471-2148-7-209

**Published:** 2007-11-04

**Authors:** Ryouka Kawahara, Mutsumi Nishida

**Affiliations:** 1 Ocean Research Institute, University of Tokyo, 1-15-1 Minamidai, Nakano-ku, Tokyo 164-8639, Japan

## Abstract

**Background:**

The threespine stickleback (*Gasterosteus aculeatus*) has a characteristic reproductive mode; mature males build nests using a secreted glue-like protein called spiggin. Although recent studies reported multiple occurrences of genes that encode this glue-like protein spiggin in threespine and ninespine sticklebacks, it is still unclear how many genes compose the spiggin multi-gene family.

**Results:**

Genome sequence analysis of threespine stickleback showed that there are at least five spiggin genes and two pseudogenes, whereas a single spiggin homolog occurs in the genomes of other fishes. Comparative genome sequence analysis demonstrated that Muc19, a single-copy mucous gene in human and mouse, is an ortholog of spiggin. Phylogenetic and molecular evolutionary analyses of these sequences suggested that an ancestral spiggin gene originated from a member of the mucin gene family as a single gene in the common ancestor of teleosts, and gene duplications of spiggin have occurred in the stickleback lineage. There was inter-population variation in the copy number of spiggin genes and positive selection on some codons, indicating that additional gene duplication/deletion events and adaptive evolution at some amino acid sites may have occurred in each stickleback population.

**Conclusion:**

A number of spiggin genes exist in the threespine stickleback genome. Our results provide insight into the origin and dynamic evolutionary process of the spiggin multi-gene family in the threespine stickleback lineage. The dramatic evolution of genes for mucous substrates may have contributed to the generation of distinct characteristics such as "bio-glue" in vertebrates.

## Background

Genome sequencing has shown that gene copy number variation (CNV) occurs more often than expected. Recently, a genome-wide examination of CNVs in humans revealed that many CNVs show linkage disequilibrium [1]. Moreover, CNVs contribute to inter-individual variation in responses to drugs, immune defence, and susceptibility to certain diseases in humans and mice [2,3]. These findings suggest that variation in gene copy number is sometimes under selection and that it is one of the driving forces for evolution in these species. However, because these studies focused on certain human and mouse diseases, it is unclear whether CNVs and these features of CNVs are common phenomena in vertebrates.

Threespine stickleback (*Gasterosteus aculeatus*), which inhabits marine, brackish, and freshwaters of the Northern hemisphere, is a classical model organism in ethology [4] and has recently attracted attention because of the evolution of diverse morphological characters among populations [5,6]. This fish is also well known for its characteristic reproductive mode in which mature males build nests using a glue-like protein called "spiggin" to adhere materials to the nest [5,7]. There are multiple occurrences of genes that encode spiggin, suggesting the existence of an ancestral gene prior to the expansion of teleosts and the duplication of spiggin genes both before and after the speciation of threespine stickleback [8]. This implies a possible relationship between spiggin gene duplication and the stickleback's specific reproductive nest-building behavior.

It is unclear how many genes compose the spiggin multi-gene family [8-10]. In previous studies, spiggin gene sequences were characterized mainly based on cDNA [8,9], and information derived from genome sequences was not considered. The results of genomic Southern analyses to estimate the number of spiggin genes differ among studies [8,10]. The genome sequence of threespine stickleback was recently published [11], making it possible to determine the number of spiggin genes and conduct comparative genomic analyses. Moreover, it may allow the exploration of the origin of and evolutionary processes occurring in the spiggin multi-gene family.

We aimed to resolve the spiggin multi-gene family in threespine stickleback and understand its origin and evolutionary processes. We isolated members of the spiggin multi-gene family from the threespine stickleback genome database and conducted phylogenetic and synteny analyses of these genes together with their homologs and related genes. We also performed molecular evolutionary analyses to examine the evolutionary forces that shaped the spiggin multi-gene family.

## Results

### Identification of the spiggin multi-gene family and homologs in genome sequences

**Figure 1:**
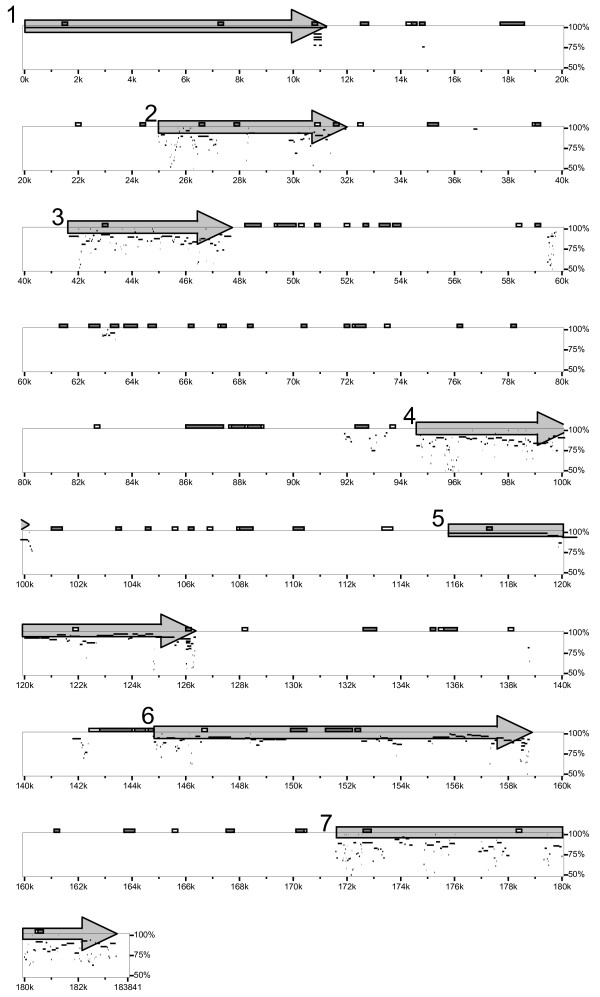
Chromosomal localization of the spiggin multi-gene family in threespine stickleback. Localization of the spiggin multi-gene family in LG IV (21,018,160–21,202,000 bp; 183,841 bp in length) of the threespine stickleback genome sequence was estimated using Gaac_spg1 as a query. Regions with > 50% similarity are plotted. The seven putative spiggin genes are numbered and shown as arrows. Boxes above the rows indicate GC-rich regions (white boxes: > 60%, gray boxes: > 75%).

**Figure 2:**
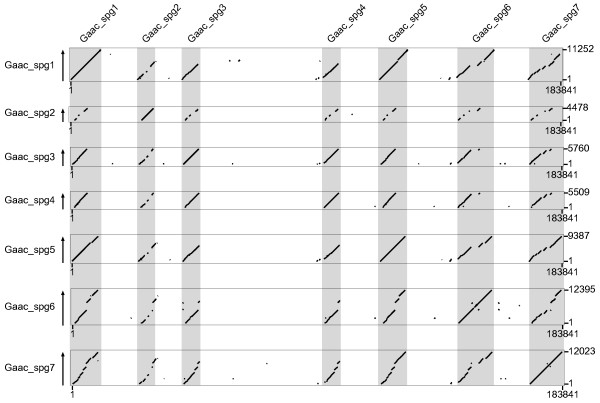
Similarity among spiggin genes in threespine stickleback. Regions of > 50% similarity with each spiggin gene sequence (Gaac_spg1-Gaac_spg7) are plotted in the region containing all members of the spiggin multi-gene family (21,018,160–21,202,000 bp in LG IV) of the threespine stickleback genome sequence. Putative spiggin genes are named and shaded.

We identified seven putative spiggin genes in linkage group (LG) IV of the threespine stickleback genome sequence. No other spiggin related genes have been found in other regions, although the whole genome was searched exhaustively. The length of the region in LG IV that contained the entire spiggin multi-gene family was approximately 200 kbp (Fig. 1). These genes were located tandemly in the same direction. We named these seven genes Gaac_spg1 to Gaac_spg7 (Fig. 1). A relatively long intergenic region (approximately 46 kbp) was observed between the third and fourth spiggin genes (Figs. 1, 2) compared with the lengths of the other intergenic regions.

Some of these spiggin genes had relatively high diversity in length and similarity. Gaac_spg2, Gaac_spg3, and Gaac_spg4 were shorter than the other spiggin genes because a region corresponding to the posterior region of the other genes was truncated (Fig. 2). Although a rather high level of similarity was observed among the spiggin genes, Gaac_spg2 had relatively low similarity with the other genes (Fig. 2). Low similarity was also found in the central region of Gaac_spg6, although the other regions of this gene had high similarity with the other copies (Fig. 2). We examined the region of low sequence similarity in detail.

To examine the detailed differences in the spiggin gene sequences, we aligned all of the genes that were identified in this study (Additional file 1). Examination of the Gaac_spg2 sequence indicated that the central region has been lost (Figs. 1, 2, and Additional file 1, P. 4–5). Even the regions that showed relatively high similarity with other spiggin genes had several indels, and some exon-intron boundaries were not conserved (Additional file 1), suggesting that this gene has been disrupted. Gaac_spg6 also contains several indels and mutations in the exon-intron boundaries (Additional file 1), as well as a low-similarity region containing GC-rich regions (Fig. 1), a repeated region (two repeat units, 332 bp in length), and a gap sequence that has not yet been sequenced (Additional file 1). We amplified and sequenced the regions that contained indels to confirm that such features were not caused by sequencing errors (data not shown). Based on these results, we judged that Gaac_spg2 and Gaac_spg6 are pseudogenes and thus excluded them from further analyses.

The percent similarity was estimated among the putative ORF regions of the remaining spiggin genes. High similarity was observed between Gaac_spg1 and Gaac_spg5 (99%), Gaac_spg1 and Gaac_spg7 (90%), and Gaac_spg5 and Gaac_spg7 (90%). Gaac_spg3 and Gaac_spg4 were also highly similar (92%). However, the similarities between the first three gene pairs (Gaac_spg1, Gaac_spg5, and Gaac_spg7) and the latter pair (Gaac_spg3 and Gaac_spg4) were relatively low (83–88%). The similarities among the translated amino acid sequences were all lower than the similarities among the nucleotide sequences, suggesting that there were more nonsynonymous than synonymous substitutions.

We identified one spiggin homolog in scaffold 898 of the medaka genome sequence. This scaffold is one of the shortest scaffolds (78 kbp) and assembled to none of the LGs of the medaka genome. Combined with the results of a previous study [8], this demonstrates the occurrence of a single spiggin homolog in four fish species: torafugu, spotted green pufferfish, medaka, and zebrafish.

We predicted the conserved domain structures of the translated sequences of the spiggin genes and the medaka spiggin homolog. All of the spiggins and the medaka spiggin homolog shared the von Willebrand factor D domain (VWD) structure. Except for the VWDs, we could not identify any domains characteristic of the translated products of secreted mucin genes, Muc2, 5AC, 5B, and 19, which are suggested to be related to the spiggin genes [8].

### Phylogenetic analyses of spiggin genes

**Figure 3:**
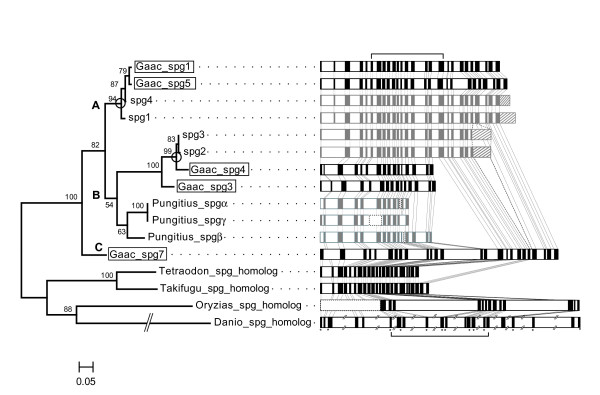
Phylogenetic tree and gene structures of the spiggin multi-gene family and its homologs. Threespine and ninespine stickleback spiggin genes from the genome sequence (Gaac_spg1, 3, 4, 5, and 7; boxed), published spiggin cDNA sequences (spg1-spg4, Pungitius_spgα-γ; DDBJ/EMBL/NCBI accession numbers: AB221477, AB221481-83, DQ018713-8), and spiggin homologs in four other fish species (Tetraodon, spotted green pufferfish; Takifugu, torafugu; Oryzias, medaka; Danio, zebrafish) were subjected to phylogenetic analyses, and the resulting ML tree is shown. Numbers at nodes in internal branches indicate % bootstrap values (500 replicates). Putative corresponding relationships between genome and cDNA sequences are indicated by circles. The exon-intron structures of the corresponding genes are shown on the right. Brackets indicate the region used for phylogenetic studies (exons 7–16). The gene structure of spiggin cDNA sequences is shown in gray. Shaded boxes in the cDNA sequences indicate that the ORF could not be estimated from the genome data. Unpublished sequence gaps from ninespine stickleback genes and an undetermined region in the medaka genome are indicated by dotted lines. Asterisks in the zebrafish gene structure indicate the parts incongruent for the determination of ORFs because of indels.

Phylogenetic analyses were conducted using the spiggin genes in threespine and ninespine sticklebacks and their homologs in other fishes (Additional file 2). We used various data from the spiggin genes in threespine stickleback: spiggin genes isolated in the threespine stickleback genome sequence (Gaac_spg1, 3, 4, 5, and 7) and spiggin cDNA sequences published in Genbank (spg1-spg4; DDBJ/EMBL/NCBI accession numbers: AB221477, AB221481-83). We also used spiggin genes from ninespine stickleback (spiggin α-γ; DDBJ/EMBL/NCBI accession numbers: DQ018713-8) and spiggin homologs isolated from the torafugu, spotted green pufferfish, medaka, and zebrafish genome sequences. No differences in topology among the analyses (neighbor joining [12], maximum likelihood [13], and Bayesian [14]) were found. The tree derived from the maximum likelihood (ML) analysis is shown (Fig. 3).

In the phylogenetic analyses, the threespine stickleback spiggin genes (Gaac_spg1, 3, 4, 5, 7, and spg1-spg4) and the ninespine stickleback genes (Pungitius_spgα-γ) formed a monophyletic group (Fig. 3). The clade of threespine and ninespine stickleback spiggin genes was divided into three subgroups: Clade A, containing Gaac_spg1, Gaac_spg5, spg1, and spg4; Clade B, containing Gaac_spg3, Gaac_spg4, spg2, spg3, and ninespine stickleback spiggins (α, β, and γ); and Clade C, containing Gaac_spg7 (Fig. 3). Clades A and B were most closely related, and Clade C was basal (Fig. 3). Although it is not clear how many spiggin genes occur in ninespine stickleback, this result suggests that the spiggin genes diverged both before and after the divergence of threespine and ninespine sticklebacks.

When we focused on the genes from the threespine stickleback genome sequence (Gaac_spg1, 3, 4, 5, and 7), the phylogenetic relationships of the genes were not congruent with their chromosomal locations (Figs. 1, 3), indicating that the genes that showed close relationships in the phylogenetic tree were not necessarily located close to each other on the chromosome. Thus, it was not possible to estimate the order of the gene duplication events based on the relative gene positions. Spiggin genes isolated from the threespine stickleback genome sequence and those from cDNA sequences did not show exact one-to-one corresponding relationships (Fig. 3). Corresponding relationships were found for only a few genes: Gaac_spg1 + Gaac_spg5 and spg1 in Clade A, and Gaac_spg4 and spg2 + spg3 in Clade B (Fig. 3).

### Gene structure

We estimated the gene structure of the spiggin genes in threespine stickleback. In the spiggin genes isolated from the genome sequence (Gaac_spg1, 3, 4, 5, and 7), the numbers and lengths of exons in the 5' region were diverse. The second exon of Gaac_spg7 and the fifth exon of Gaac_spg3 were absent, and the second exon of Gaac_spg4 was 20 nucleotides shorter than that of the other genes (Fig. 3). The length of introns also differed among the genes; introns in Gaac_spg7 were longer than those in the other genes (Fig. 3). In Gaac_spg3 and Gaac_spg4, exons in the posterior region were truncated, which was apparent in the dotplot analysis (Figs. 2, 3). When information about the putative exon-intron boundary in the threespine and ninespine stickleback spiggin cDNA sequences was taken into account, there was a tendency for spiggin genes in Clade A to have similar exon-intron structures, whereas those in Clade B had diversity in the length and the number of exons (Fig. 3). Although the gene structures of ninespine stickleback spiggins were unclear because only partial cDNA sequences have been published, Gaac_spg3 and Gaac_spg4 had fewer exons than did the other genes, and spg1, 2, 3, and 4 contained apparently untranscribed regions of the other genes. Probable changes in the consensus sequences of the exon-intron boundary or terminal codon may have caused the elongation of the ORF of these genes.

We also examined the gene structures of the spiggin gene homologs of other fishes. In the spiggin homologs of torafugu and spotted green pufferfish, the lengths and sequence similarities of exons were conserved. Based on sequence similarity in some exons, spiggin homologs of medaka and zebrafish also seemed to conserve the basic exon-intron structure with spiggin genes of threespine stickleback, although reliable estimation of the ORF region was difficult in these two species because of possible gaps in their genome sequence data. The lengths of introns were dramatically short in torafugu and spotted green pufferfish, whereas the homologs of medaka and zebrafish had very long introns (Fig. 3).

### Detection of positive selection and gene recombination

We conducted an evolutionary analysis to examine the possibility that the threespine stickleback spiggin genes were under positive selection and obtained evidence of positive selection (*p *< 0.01) in two subgroups: Clades A and B (Table 1). In Clade B, the 140th amino acid residue was estimated to be under positive selection with a high posterior probability (PP > 0.99). Although translated sequences of these genes conserved the VWD, which creates multimers with other molecules containing a VWD, the 140th amino acid residue was not located in this conserved region.

**Table 1:** Estimation of positively selected branches and sites using branch-site models.

Foreground branches	2ΔL	Parameter estimates in the modified model	Positively selected sites
Whole spiggin multi-gene family	4.578	P_0 _= 0.53341, P_1 _= 0.40016, P_2a _= 0.03795, P_2b _= 0.02847, ω_0 _= 0.14243, ω_2 _= 893.45416.	
Lineage A	7.544*	P_0 _= 0.55829, P_1 _= 0.42899, P2a = 0.00720, P2b = 0.00553, ω_0 _= 0.14530,ω_2 _= 588.82297	130, 202, 247
Lineage B	9.859**	P_0 _= 0.47669, P_1 _= 0.35041, P2a = 0.09965, P2b = 0.07325, ω0 = 0.14136,ω2 = 9.76851	2, 3, 4, 8, 10, 16, 37, 39, 66, 67, 71, 79, 81, 88, 109, 111, 122, 128, 132, 139, 140§, 157, 174, 184, 186, 188, 191, 198, 200, 212, 214, 215, 221, 227, 228, 229, 251, 260, 296

*, *p *< 0.05; **, *p *< 0.01.

§Posterior probability > 0.99

Evidence of gene conversion was found in two regions of Gaac_spg3 and Gaac_spg4 (*p *< 0.001; Table 2). The regions were 152 and 138 bp long, respectively.

**Table 2:** Putative regions of gene conversion as determined using GENCONV.

Sequences	Simulated *p*	BCKA^a ^*p*	Aligned begin/end	Offsets length	No. poly^b^	No. dif^c^	Total difs^d^
Gaac_spg3: Gaac_spg4	0	0.00016	808/959	152	33	0	152
Gaac_spg3: Gaac_spg4	0	0.00026	1546/1683	138	32	0	152

a. Bonferroni-corrected Karlin-Altschul.

b. Number of polymorphic sites within the fragment.

c. Number of internal mismatches within the fragment.

d. Overall number of different sites in the sequences.

### Comparison of synteny

**Figure 4:**
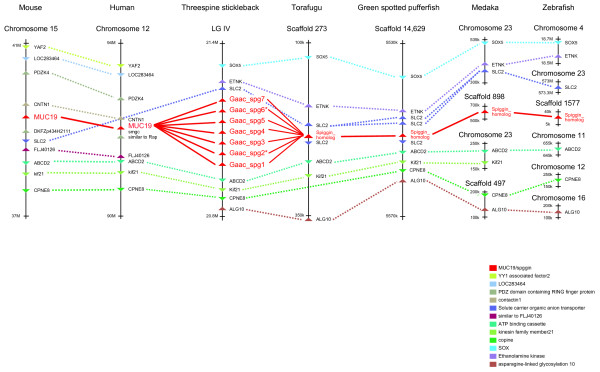
Diagrammatic representation of the chromosomal location of the spiggin multi-gene family, its homologs, and Muc19. Spiggin (threespine stickleback), spiggin homologs (torafugu, spotted green pufferfish, medaka, and zebrafish), and Muc19 (mouse and human) are indicated in red, and other genes are indicated in other colors. Orthologous genes are connected by dotted lines. Spiggin, its homologs, and Muc19 are connected by solid lines.

The chromosomal locations of the spiggin multi-gene family, its homologs, and their related genes were examined and compared among the species. Spiggin genes or their homologs were located in LG IV (threespine stickleback), scaffold 273 (torafugu), scaffold 14,629 (spotted green pufferfish), scaffold 898 (medaka), and scaffold 9975 (zebrafish). The chromosomal locations of all of these genes, except for those of threespine stickleback, are unknown because the scaffolds have not been annotated to a specific chromosome. In human and mouse, the chromosomal location of the secreted mucin gene family (i.e., Muc2, 5AC, 5B, 6, and 19), which is thought to be related to the spiggin multi-gene family [8], was examined. In human, Muc2, 5AC, 5B, and 6 were located on the 11th chromosome as a cluster, whereas Muc19 was located in the 12th chromosome. In mouse, Muc2, 5AC, 5B, and 6 clustered on the 7th chromosome, whereas Muc19 was located on the 15th chromosome. We also explored the genes around the spiggin multi-gene family, its homologs, and mucin genes in each species. Although other genes were not identified in the scaffolds of medaka or zebrafish because of their shortness, some genes in the syntenic region were identified by virtue of the other species' synteny information (Fig. 4). As a result, we found that torafugu, spotted green pufferfish, medaka, and zebrafish shared several genes around the spiggin gene homologs with threespine stickleback, as well as Muc19 in human and mouse (Fig. 4). These facts strongly suggest that spiggin and Muc19 are orthologous. In contrast, the cluster of Muc2/5AC/5B/6 and Muc19 did not share any genes around them, suggesting a paralogous relationship between them.

## Discussion

### Spiggin gene repertoire in threespine stickleback

There are at least five spiggin genes and two pseudogenes in the threespine stickleback genome sequence. However, spiggin genes from the genome sequence (Gaac_spg1, 3, 4, 5, and 7) and those from cDNA (spg1-spg4) did not show one-to-one correspondence (Fig. 3). Although these two types of spiggin gene are derived from different sources, they should show corresponding relationships because they were sequenced from a single species. The spiggin cDNAs (spg1-spg4) are extremely unlikely to contain alleles at spiggin loci because of their sequence diversity (Fig. 3).

One possible reason for the absence of such correspondence is that the two populations used for sequencing are highly differentiated genetically. A population in Bear Paw Lake, Alaska, USA, was used for the genome project [15-17]. The population used for cDNA sequencing was from the Pacific Ocean group in eastern Hokkaido, Japan [8,18]. Allozyme and SNP analyses show that they are genetically distinct populations [5,18,19]. In addition, our results suggest the possibility of population-specific evolution of the spiggin multi-gene family. Gene duplication/deletion, amino acid replacement, and gene conversion may have occurred independently in each stickleback population (Fig. 3; Tables 1, 2). To confirm this possibility, further study of the genome sequence of the Pacific Ocean group is necessary.

### Origin and evolution of the spiggin multi-gene family

Our previous phylogenetic analyses using partial amino acid sequences of the conserved domain structure suggested that the Muc19 gene is most closely related to the spiggin multi-gene family; phylogenetic analyses using full length spiggin/mucin genes could not be made because of high gene diversity [8]. We confirmed this hypothesis through the analysis of the chromosome location and synteny of the spiggin and related gene families of various vertebrate species. Thus, we conclude that spiggin genes originated from members of the mucin gene family. Translated spiggin gene products are high-molecular-mass glycoproteins that constitute glue-like proteins [20]; translated mucin gene products are also high-molecular-mass glycoproteins that represent major components of mucus-like substances [21]. This similarity clearly reflects the orthologous relationships of these genes.

Five spiggin genes and two pseudogenes were observed in the threespine stickleback genome sequence, and three types of spiggin genes have been identified in ninespine stickleback. In contrast, Muc19 is a single-copy gene in human and mouse, and a single spiggin homolog was found in fishes other than sticklebacks. These facts and the results of our phylogenetic analysis imply that the ancestral spiggin gene existed as a single gene in the ancestral fish lineage, and duplications of the spiggin gene occurred both before and after the divergence of threespine and ninespine sticklebacks (Fig. 3).

Spiggin mRNA is found in the kidney in threespine stickleback [8,10,20]. However, Muc19 mRNA is found in the submaxillary gland in human and mouse [22,23]. Thus, the expression pattern of the ancestral spiggin gene may have changed after the divergence of tetrapods and fishes. In human, secretory mucin genes other than Muc19 (i.e., Muc2, 5AC, 5B, and 6) are thought to have evolved from a common ancestral gene by two successive duplications [24]. These mucin genes show both spatially [25-27] and temporally specific expression patterns [28-31]. It is clear that these genes were neofunctionalized after the gene duplications. A recent study also showed lineage-specific gene duplication of the mucin gene family in chicken and found that the additional gene encodes ovomucin, which is abundant in egg white and responsible for its gel-like properties [32]. These findings show that expression pattern shifts and gains of new function have occurred repeatedly following gene duplication in the mucin gene family in various vertebrate lineages.

We examined the expression of spiggin homologs in torafugu and zebrafish. Although we did not find expression in any of the zebrafish tissues examined, we did find kidney-specific expression in male and female torafugu (Additional file 3). In threespine stickleback, spiggin genes are expressed only in the kidneys of mature males, and translated products of spiggin are secreted as a glue in male nest building [8,10,20]. Such a glue-like protein produced in the kidney has not been reported in torafugu. This implies that the function of spiggin should differ between threespine stickleback and torafugu. The shifts in expression and gain of new function as a glue may have occurred in the spiggin gene family in sticklebacks in a fashion similar to the mucin gene family in human or ovomucin in chicken. Of course, we cannot exclude the possibility that mutation events in regulatory regions of these genes, which could cause such divergent expression, also occurred in spiggins of other fish lineages. Further expression and functional analyses will explore the detailed evolutionary processes of the spiggin multi-gene family in threespine stickleback.

### Background of the evolution of the spiggin multi-gene family

Our results demonstrate that the spiggin multi-gene family of threespine stickleback is localized in a cluster. In general, such repeated genes can be divided into two types: variant and invariant repeats [33]. Variant repeats are copies of a gene that differ in sequence from the original, and sometimes can perform markedly different functions [34]. Invariant repeats are gene copies with identical or nearly identical sequences, which can result in the synthesis of large quantities of gene product [35].

Although it is reasonable to hypothesize that spiggin genes are invariant repeats because a large amount of glue is required for nest building, considerable diversity was observed in their sequences. The existence of many nonsynonymous substitutions in the sequences confirmed that this multi-gene family is constructed of variant repeats. Our evolutionary analysis suggests that diversifying evolution occurred by positive Darwinian selection, rather than the random fixation of neutral mutations.

Adaptive evolution to the local environment seems to exist in the background of the evolution of the spiggin multi-gene family. There are distinct wild populations of threespine stickleback that have morphological, behavioral, and physiological differences. Various characters in each population may have evolved to match the local environment; one character is nest-building behavior [36]. During nest building, a glue-like protein is used to adhere materials to the nest [20]. To maximize its function, the character of the glue should be adapted to the local environment (e.g., temperature, pH, and salinity). The spiggin multi-gene family might have evolved in conjunction with the adaptation of the glue-like protein to various environments.

Nest-building behavior might also be under sexual selection by females because this behavior is an important component of reproduction. Mature females are more attracted to males with decorated nests than to males with undecorated nests [37,38]. Moreover, the relative weight of a nest building male's kidney, which secretes the glue-like protein, is positively correlated with nest characters such as neatness or compactness [39], which may reflect male quality. Because mature males use the glue-like protein in nest building, spiggin genes may have also evolved under sexual selection.

## Conclusion

Five spiggin genes and two pseudogenes were identified in the threespine stickleback genome sequence and single-copy spiggin homologs were found in other fishes. In addition, Muc19, a single-copy gene in human and mouse, was demonstrated to be a putative ortholog of spiggin. These results suggest that the ancestral spiggin gene originated from the mucin gene family as a single-copy gene and that subsequent gene duplication events occurred before and after threespine stickleback speciation. We also suggest a complex evolutionary trajectory of the spiggin multi-gene family, including positive selection after duplication events in stickleback populations. Further study of additional threespine stickleback populations will demonstrate population-specific evolution of the spiggin multi-gene family in detail and may provide insight into the evolutionary forces shaping the gene family. The dramatic evolution of genes such as mucin or the spiggin multi-gene family that encode mucous substrates may have helped to generate the characteristic of "bio-glue" in vertebrates.

## Methods

### Identification of spiggin genes and their homologs in genome databases

Genome sequences of threespine stickleback and medaka, which are available from the Ensembl site [40] (threespine stickleback: BROAD S1, assembled in Feb 2006; medaka:FHdrR, assembled in Oct 2005), were used for a TblastN search [41]. Published spiggin cDNA sequences (spg1-spg4: DDBJ/EMBL/NCBI accession numbers: AB221477, AB221481-83) were used as queries. Protocols for the identification of genes followed those of a previous study [8]. Isolated sequences were translated and subjected to a BLASTP search. Sequences that were considered products of homologs of the spiggin gene were used for further analyses. The prediction of coding regions was performed using wise2 [42] and confirmed by eye.

### Sequence analysis

The chromosome location and direction of the spiggin multi-gene family in threespine stickleback were estimated using PIPmaker [43]. We extracted a genomic region that contained all members of the spiggin multi-gene family from the genome sequence available in Ensembl (LGIV_21,018,160–21,202,000 bp; 183,841 bp in length). Similarity to this extracted region was plotted using one of the isolated spiggin genes (Gaac_spg1) as a query. Similarities among the spiggin genes were also examined using a dotplot. The extracted genomic region was compared with each of the seven isolated spiggin genes, and more than 50% similarity was plotted.

Estimation of the exon-intron structure was performed using wise2. Translated sequences of the isolated spiggin multi-gene family in threespine stickleback and its homologs in other fishes (torafugu, spotted green pufferfish, medaka, and zebrafish) were used as queries. Exon-intron boundaries of the threespine and ninespine stickleback spiggin cDNA sequences were also estimated based on these results.

The regions that contained the indels of Gaac_spg2 and Gaac_spg6 (exons 3 and 16 in Gaac_spg2; exons 13, 16 + 17, and 22 in Gaac_spg6; Additional file 1) were confirmed by additional sequencing of one individual. The stickleback collected from Bear Paw Lake, Alaska, USA, which was the resource for the genome project, was used for total DNA extraction using an Aquapure genomic DNA isolation kit (Bio-Rad Laboratories, Inc., Hercules, CA, USA). PCR reactions were performed using primers designed to amplify regions with indels (Additional file 1, 4), using TaKaRa Ex-taq (TaKaRa Bio Inc., Otsu, Shiga, Japan) in a GeneAmp PCR System 9700 (Applied Biosystems, Foster City, CA, USA) according to the manufacturer's instructions. The reaction conditions for PCR were: 94°C for 2 min; 30 cycles of 94°C for 15s, 53°C for 15s, 72°C for 1 min, and 72°C for 10 min. The amplified PCR products were cloned using a TOPO TA Cloning Kit for Sequencing (Invitrogen Corporation, Carlsbad, CA, USA). Cycle sequencing was conducted using dye-labeled terminators (Big Dye terminator ver.3.1, Applied Biosystems) and the reaction products were sequenced using an ABI PRISM 3130 Genetic Analyzer (Applied Biosystems).

We estimated the expression pattern of the spiggin homolog in torafugu and zebrafish. We extracted total RNA from kidney, liver, muscle, skin, gonad, heart, brain, fin, eye, gill (zebrafish), and stomach (torafugu) tissues of male and female torafugu and zebrafish using TRIZOL Reagent (Invitrogen). Total RNA was reverse transcribed using a TaKaRa RNA-PCR kit ver2.1 (TaKaRa Bio) and then subjected to PCR using a pair of primers designed to amplify a partial region of each obtained spiggin homolog gene (Additional file 4) as described above. The reaction conditions for PCR were: 94°C for 2 min; 30 cycles of 94°C for 15s, 51°C (torafugu) or 57°C (zebrafish) for 15s, 72°C for 1 min, and 72°C for 10 min. As a negative control, PCR amplification was also conducted using each RNA sample without reverse transcription. As a positive control, glyceraldehyde-3-phosphate dehydrogenase (GAPDH) mRNA was amplified using a pair of primers described previously [8].

### Phylogenetic analyses

Phylogenetic analyses were conducted using spiggin genes in threespine and ninespine stickleback and their homologs in other fishes. We used threespine and ninespine stickleback spiggin genes that were isolated from the genome sequence (Gaac_spg1, 3, 4, 5, and 7), published spiggin cDNA sequences (spg1-spg4; Pungitius_spgα-γ), and spiggin homologs isolated in torafugu (Takifugu_spg_homolog), spotted green pufferfish (Tetraodon_spg_homolog), medaka (Oryzias_spg_homolog), and zebrafish (Danio_spg_homolog; Additional file 2). Nucleotide sequences were translated and aligned using ClustalW implemented in MEGA3.1 [44]. To avoid misaligning, we first extracted VWDs, which are conserved in all of the sequences. Multiple alignment was performed for each VWD, excluding truncated domains, and a phylogenetic tree was constructed. The preliminary analysis indicated that the relative positions of VWDs are conserved in all of the genes except the medaka spiggin homolog (Additional file 5). Based on these results, we aligned the translated sequences of full-length spiggin and their homologs.

Because the ninespine stickleback spiggin genes were published as partial sequences, we concatenated them based on conserved domain region information. Gap sequences of ninespine stickleback spiggins were treated as missing data. Ambiguous regions were excluded, and the resulting aligned sequences (corresponding to exons 6–17; 211 amino acids in length) were subjected to neighbor-joining (NJ), maximum-likelihood (ML), and Bayesian analyses [14], using MEGA3.1, Treefinder [45], and MrBayes, respectively. In all analyses, the JTT model [46] was selected as a substitution model. In MrBayes, analyses were done for 1,000,000 generations and sampled every 1000th generation. The log likelihoods were observed graphically, and trees that were excluded before reaching "stationarity" were used to construct a 50% majority consensus tree and calculate posterior probabilities (PP).

### Estimating the pattern of nucleotide substitution and positively selected sites

The diversity of threespine stickleback spiggin genes was examined with molecular evolutionary analyses using ω, which is the ratio of nonsynonymous substitutions (dN) to synonymous substitutions (dS). We used the threespine stickleback spiggin genes isolated from the genome database (Gaac_spg1, 3, 4, 5, and 7) and the published spiggin cDNA sequences (spg1-spg4). In addition to these sequences, the spiggin homologs isolated in other fishes (torafugu, spotted green pufferfish, medaka, and zebrafish) were added and aligned using ClustalW in MEGA3.1, with gaps excluded. Considering that positive selection may act in very short episodes during the evolution of a protein and affect only a few sites along a lineage, we used a recently developed likelihood-accommodating ω ratio to vary among both lineages of interest and amino acid sites, i.e., an improved version of the "branch-site" model [47].

We tested for positive selection in the whole threespine stickleback spiggin gene lineage and in two subgroups (Clades A and B) as foreground branches. The branch-site model compares two models. The alternative model assumes four classes of sites in terms of ω. In the alternative model, site class 0 contains codons that are conserved throughout the tree, with 0 < ω0 < 1. Site class 1 contains codons that are neutral throughout the tree with ω1 = 1. Site classes 2a and 2b contain codons that are conserved or neutral on the background branches (2a, 0 < ω2 < 1; 2b, ω2 = 1), but are under positive selection on the foreground branches with ω2 > 1, estimated from the data. The null model differs from the alternative model in that ω2 is fixed at 1. Thus, in the likelihood-ratio test (LRT), a significantly higher likelihood of the alternative model than that of the null model indicates positive selection on the foreground branches. The Bayes empirical Bayes (BEB) approach was used to calculate the posterior probability that each site belongs to the site class of positive selection on the foreground lineages.

### Detection of recombination

The gene conversion detection method as implemented in GENECONV [48] was used to look for evidence of gene conversion in the spiggin multi-gene family in the threespine stickleback genome sequence. Aligned sequences of the isolated spiggin genes (Gaac_spg1, 3, 4, 5, and 7) were subjected to analysis using the default settings. The GENECONV program computes global and pairwise *p*-values and allows mismatches within converted regions. Global *p*-values are conservative and accurate because they are corrected for multiple comparisons, whereas the *p*-values of pairwise fragments are not. We considered *p *< 0.05 significant for both methods.

### Gene mapping

The chromosome locations of the spiggin genes, spiggin homologs, and relative genes were examined using the genome databases. Mapping information for the spiggin multi-gene family in threespine stickleback and its homologs in torafugu, spotted green pufferfish, medaka, and zebrafish was available on the Ensembl website [40]. In human and mouse, localization of the secreted mucin gene family (Muc2, 5AC, 5B, 6, and 19) was estimated based on the MAP viewer [49].

## List of Abbreviations

Copy number variation: CNV; Linkage group: LG; Open reading frame: ORF; Von Willebrand factor D-domain: VWD; Neighbor-joining: NJ; Maximum-likelihood: ML; Posterior probability: PP; Glyceraldehyde-3-phosphate dehydrogenase: GAPDH; Likelihood-ratio test: LRT; Bayes empirical Bayes: BEB.

## Authors' contributions

RK and MN designed the study. RK carried out the molecular work, analyzed the data and wrote the manuscript. MN helped write the manuscript. All authors read and approved the final manuscript.

## Supplementary Material

Additional file 1 "Alignment of spiggin genes identified in this study (Gaac_spg1-7)". Aligned sequences of the spiggin multi-gene family (Gaac_spg1-7) identified in the threespine stickleback genome. Putative exon regions are shaded in pink and numbered according to the gene structure of Gaac_spg1. Exon-intron boundaries with mutation sites are boxed in green and indels are boxed in red. In Gaac_spg6, repeated regions (two repeat units, 332 bp in length) are boxed in blue. Asterisks indicate sites that are conserved in all of the sequences. Click here for file

Additional file 2 "Sequences of spiggin genes in threespine and ninespine sticklebacks and their homologs in other fishes". Sequences of the spiggin multi-gene family in threespine stickleback (Gaac_spg1, 3, 4, 5, and 7; spg1-4), ninespine stickleback (Pungitius_spgα-γ), and their homologs in torafugu (Takifugu_spiggin_homolog), spotted green pufferfish (Tetraodon_spiggin_homolog), medaka (Oryzias_spiggin_homolog), and zebrafish (Danio_spiggin_homolog) that were used in the phylogenetic analyses. The resulting data matrix, excluding ambiguous regions, was aligned before phylogenetic analyses were performed. Click here for file

Additional file 3 "Expression analysis of spiggin homologs in torafugu and zebrafish". Expression of spiggin homologs in various tissues of torafugu and zebrafish determined by RT-PCR. Glyceraldehyde-3-phosphate dehydrogenase (GAPDH) was used as a positive control. Plus signs indicate amplification using reverse-transcribed cDNA from each tissue; minus signs indicate negative controls using RNA samples without reverse-transcription. Click here for file

Additional file 4 "Primers used". Sequences of primers that were used in this study. Click here for file

Additional file 5 "Phylogenetic tree using conserved domain structures". Reconstructed phylogenetic tree based on the conserved domain structures (VWD) of spiggins and their homologs. We used translated sequences of threespine and ninespine stickleback spiggin genes that were isolated from the genome sequence (Gaac_spg1, 3, 4, 5, and 7), published spiggin cDNA sequences (spg1-spg4; Pungitius_spgα-γ), and spiggin homologs isolated in torafugu (Takifugu_spg_homolog), spotted green pufferfish (Tetraodon_spg_homolog), medaka (Oryzias_spg_homolog), and zebrafish (Danio_spg_homolog). We extracted conserved domain structures (VWD), numbered them from the N-terminal end, and subjected them to phylogenetic analysis. The ML tree is shown; numbers at nodes in internal branches indicate % bootstrap values (500 replicates). The asterisk indicates the VWD domain of medaka spiggin homolog, for which the relative position is incongruent with those of other domains. Click here for file

## References

[B1] Redon R, Ishikawa S, Fitch KR, Feuk L, Perry GH, Andrews TD, Fiegler H, Shapero MH, Carson AR, Chen WW (2006). Global variation in copy number in the human genome. Nature.

[B2] Aitman TJ, Dong R, Vyse TJ, Norsworthy PJ, Johnson MD, Smith J, Mangion J, Roberton-Lowe C, Marshall AJ, Petretto E (2006). Copy number polymorphism in Fcgr3 predisposes to glomerulonephritis in rats and humans. Nature.

[B3] Gonzalez E, Kulkarni H, Bolivar H, Mangano A, Sanchez R, Catano G, Nibbs RJ, Freedman BI, Quinones MP, Bamshad MJ (2005). The influence of CCL3L1 gene-containing segmental duplications on HIV-1/AIDS susceptibility. Science.

[B4] Tinbergen N (1951). The study of instinct.

[B5] Colosimo PF, Hosemann KE, Balabhadra S, Villarreal JG, Dickson M, Grimwood J, Schmutz J, Myers RM, Schluter D, Kingsley DM (2005). Widespread parallel evolution in sticklebacks by repeated fixation of ectodysplasin alleles. Science.

[B6] Peichel CL, Nereng KS, Ohgi KA, Cole BLE, Colosimo PF, Buerkle CA, Schluter D, Kingsley DM (2001). The genetic architecture of divergence between threespine stickleback species. Nature.

[B7] Wootton RJ (1976). The biology of the sticklebacks.

[B8] Kawahara R, Nishida M (2006). Multiple occurrences of spiggin genes in sticklebacks. Gene.

[B9] Kawasaki F, Katsiadaki I, Scott AP, Matsubara T, Osatomi K, Soyano K, Hara A, Arizono K, Nagae M (2003). Molecular cloning of two types of spiggin cDNA in the three-spined stickleback, Gasterosteus aculeatus. Fish Physiology and Biochemistry.

[B10] Jones I, Lindberg C, Jakobsson S, Hellqvist A, Hellman U, Borg B, Olsson PE (2001). Molecular cloning and characterization of spiggin – An androgen-regulated extraorganismal adhesive with structural similarities to von Willebrand factor-related proteins. Journal of Biological Chemistry.

[B11] Kingsley DM, Zhu B, Osoegawa K, de Jong PJ, Schein J, Marra M, Peichel C, Amemiya C, Schluter D, Balabhadra S, Friedlander B, Cha YM, Dickson M, Grimwood J, Schmutz J, Talbot WS, Myers R (2004). New genomic tools for molecular studies of evolutionary change in threespine sticklebacks. Behaviour.

[B12] Saitou N, Nei M (1987). The neighbor-joining method: a new method for reconstructing phylogenetic trees. Mol Biol Evol.

[B13] Felsenstein J (1981). Evolutionary trees from DNA sequences: A maximum likelihood approach. Journal of Molecular Evolution.

[B14] Ronquist F, Huelsenbeck JP (2003). MrBayes 3: Bayesian phylogenetic inference under mixed models.. Bioinformatics.

[B15] Ostlund-Nilsson S, Mayer I, Huntingford FA, Lutz PL (2007). Biology of the three-spined stickleback. Marine Biology.

[B16] Zody MC, Mauceli E, Chang JL, Amemiya C, Schmutz J, Grimwood J, White S, Birney E, Kingsley D, Lindblad-Toh K (2006). Sequence of the stickleback genome: Anchorage, Alaska..

[B17] Cresko WA, Amores A, Wilson C, Murphy J, Currey M, Phillips P, Bell MA, Kimmel CB, Postlethwait JH (2004). Parallel genetic basis for repeated evolution of armor loss in Alaskan threespine stickleback populations. Proc Natl Acad Sci U S A.

[B18] Higuchi M, Goto A (1996). Genetic evidence supporting the existence of two distinct species in the Gasterosteus around Japan. Environmental Biology of Fishes.

[B19] Haglund TR, Buth DG, Lawson R (1992). Allozyme variation and phylogenetic relationships of Asian, North American, and European populations of the threespine stickleback, Gasterosteus aculeatus. Copeia.

[B20] Jakobsson S, Borg B, Haux C, Hyllner SJ (1999). An 11-ketotestosterone induced kidney-secreted protein: the nest building glue from male three-spined stickleback, Gasterosteus aculeatus. Fish Physiology and Biochemistry.

[B21] Bansil R, Stanley E, Lamont JT (1995). Mucin biophysics. Annual Reviews of Physiology.

[B22] Culp DJ, Latchney LR, Fallon MA, Denny PA, Denny PC, Couwenhoven RI, Chuang S (2004). The gene encoding mouse Muc19: cDNA, genomic organization and relationship to Smgc. Physiological Genomics.

[B23] Chen Y, Zhao YH, Kalaslavadi TB, Halmati E, Nehrke K, Le AD, Ann DK, Wu R (2004). Genome-wide search and identification of a novel gel-forming mucin MUC19/Muc19 in glandular tissues. Am J Respir Cell Mol Biol.

[B24] Desseyn JL, Buisine MP, Porchet N, Aubert JP, Degand P, Laine A (1998). Evolutionary history of the 11p15 human mucin gene family. J Mol Evol.

[B25] Vandenhaute B, Buisine MP, Debailleul V, Clment B, Moniaux N, Dieu MC, Degand P, Porchet N, Aubert JP (1997). Mucin gene expression in biliary epithelial cells. Journal of Hepatology.

[B26] Bartman AE, Buisine MP, Aubert JP, Niehans GA, Toribara NW, Kim YS, Kelly EJ, Crabtree JE, Ho SB (1998). The MUC6 secretory mucin gene is expressed in a wide variety of epithelial tissues. Journal of Pathology.

[B27] Audie JP, Janin A, Porchet N, Copin MC, Gosselin B, Aubert JP (1993). Expression of human mucin genes in respiratory, digestive, and reproductive tracts ascertained by in situ hybridization. Journal of Hystochemistry and Cytochemistry.

[B28] Reid CJ, Gould S, Harris A (1997). Developmental expression of mucin genes in the human respiratory tract. American Journal of Respiratory Cell and Molecular Biology.

[B29] Buisine MP, Devisme L, Copin MC, Durand-Reville M, Gosselin B, Aubert JP, Porchet N (1999). Developmental mucin gene expression in the human respiratory tract. American Journal of Respiratory Cell and Molecular Biology.

[B30] Buisine MP, Desseyn JL, Porchet N, Degand P, Laine A, Aubert JP (1998). Genomic organization of the 3 '-region of the human MUC5AC mucin gene: additional evidence for a common ancestral gene for the 11p15.5 mucin gene family. Biochemical Journal.

[B31] Reid CJ, Harris A (1998). Developmental expression of mucin genes in the human gastrointestinal system. GUT.

[B32] Lang T, Hansson GC, Samuelsson T (2006). An inventory of mucin genes in the chicken genome shows that the mucin domain of Muc13 is encoded by multiple exons and that ovomucin is part of a locus of related gel-forming mucins. BMC Genomics.

[B33] Graur D, Li WH (1999). Fundamentals of molecular evolution.

[B34] Li WH, Nei M, Koehn RK (1983). Evolution of duplicate genes and pseudo-genes. Evolution of genes and proteins.

[B35] Ohno S (1970). Enormous diversity in genome sizes of fish as a reflection of nature's extensive experiments with gene duplication. Transactions of the American Fisheries Society.

[B36] Ishikawa M, Mori S (2000). Mating success and male courtship behaviors in three populations of the threespine stickleback. Behaviour.

[B37] Kraak SBM, Bakker TCM, Mundwiler B (1999). Sexual selection in sticklebacks in the field: correlates of reproductive, mating, and paternal success. Behavioral Ecology.

[B38] Ostlund-Nilsson S, Holmlund M (2003). The artistic three-spined stickleback (Gasterosteous aculeatus). Behavioral Ecology and Sociobiology.

[B39] Barber I, Nairn D, Huntingford FA (2001). Nests as ornaments: revealing construction by male sticklebacks.. Behavioral Ecology.

[B40] Ensembl. http://www.ensembl.org/index.html.

[B41] Altschul SF, Madden TL, Schaffer AA, Zhang JH, Zhang Z, Miller W, Lipman DJ (1997). Gapped BLAST and PSI-BLAST: a new generation of protein database search programs. Nucleic Acids Research.

[B42] Birney E, Clamp M, Durbin R (2004). Genewise and genomewise. Genome Research.

[B43] Schwartz S, Zhang Z, Frazer KA, Smit A, Riemer C, Bouck J, Gibbs R, Hardison R, Miller W (2000). PipMaker – A web server for aligning two genomic DNA sequences. Genome Research.

[B44] Kumar S, Tamura K, Nei M (2004). MEGA3: integrated software for molecular evolutionary genetics analysis and sequence alignment. Briefings in Bioinformatics.

[B45] Jobb G, von Haeseler A, Strimmer K (2004). TREEFINDER: a powerful graphical analysis environment for molecular phylogenetics. Bmc Evolutionary Biology.

[B46] Jones DT, Taylor WR, Thornton JM (1992). The rapid generation of mutation data matrices from protein sequences. Computational and Applied Bioscience.

[B47] Zhang JZ, Nielsen R, Yang ZH (2005). Evaluation of an improved branch-site likelihood method for detecting positive selection at the molecular level. Molecular biology and evolution.

[B48] Sawyer S (1989). Statistical tests for detecting gene conversion. Molecular biology and evolution.

[B49] NCBI MAP viewer. http://www.ncbi.nlm.nih.gov/mapview/.

