# Supplementary material for: Extensive lineage-specific gene duplication and evolution of the spiggin multi-gene family in stickleback
Source: BMC Evol Biol. 2007 Nov 4;7:209. doi: 10.1186/1471-2148-7-209 (PMC2180178; doi:10.1186/1471-2148-7-209)
Supplement: Additional file 1 — "Alignment of spiggin genes identified in this study (Gaac_spg1-7)". Aligned sequences of the spiggin multi-gene family (Gaac_spg1-7) identified in the threespine stickleback genome. Putative exon regions are shaded in pink and numbered according to the gene structure of Gaac_spg1. Exon-intron boundaries with mutation sites are boxed in green and indels are boxed in red. In Gaac_spg6, repeated regions (two repeat units, 332 bp in length) are boxed in blue. Asterisks indicate sites that are conserved in all of the sequences. [file 1471-2148-7-209-S1.pdf]

```
Gaac_spg1 ---------------------------------------------------------------------------------------------------------------------GTTTTAAATGAATATGTTAAGTTCAATGTTTAA   33
Gaac_spg2 ------------------------------------------------------------------------------------------------------------------------------------------------------
Gaac_spg3 ------------------------------------------------------------------------------------------------------------------------------------------------------
Gaac_spg4 ------------------------------------------------------------------------------------------------------------------------------------------------------
Gaac_spg5 CAAACCAGAAAAGAGCTTCCCCTCTTTTTACAAAATGCCCTTTATATTTACCCATTATGTGTGGTGGGTGACTCAAATATTTACAGGTTGTTCTTATTTCTGGCAGATCACATTATGTTTTAAATGAATATGTTAAGTTCAATGTTTAA  150
Gaac_spg6 ------------------------------------------------------------------------------------------------------------------------------------------------------
Gaac_spg7 ------------------------------------------------------------------------------------------------------------------------------------------------------
          1.......10........20........30........40........50........60........70........80........90.......100.......110.......120.......130.......140.......150
```

1

```
Gaac_spg1 TTTGATTTTCTTTCTACTTCCAGTTTCCTAGTATATTGTGTGAATGAGTACGGGCGAGGCGGTGCCATTTCTTTTAGGATTAACACTGCATATATAAGCCATGTGGTGGGTTGTTTCTTTCAACTGCTCTCGTGCAGCATCAGGCAGCAT  183
Gaac_spg2 ----------------------------------------------------------------------------------------------------------------------------------------------------AT    2
Gaac_spg3 ----------------------------------------------------------------------------------------------------------------------------------------------------AT    2
Gaac_spg4 ----------------------------------------------------------------------------------------------------------------------------------------------------AT    2
Gaac_spg5 TTTGATTTTCTTTCTACTTCCAGTTTCCTAGTATATTGTGTGAATGAGTACGGGCGAGGCGGTGCCATTTCTTTTAGGATTAACACTGCATATATAAGCCATGTGGTGGGTTGTTTGTTTCAACTGCTCTCGTGCAGCATCAGACAGCAT  300
Gaac_spg6 ------------------------------------------------------------------------------------------------------------------------------------------------------
Gaac_spg7 ----------------------------------------------------------TACAGACATTTTTTTTAGGAATGACACTGCACATATAAGCCATGTGGTGTTTTTTTTGTTTCAACTGCTCTCCTGCAGCATCAGGCAGCAT   92
          .......160.......170.......180.......190.......200.......210.......220.......230.......240.......250.......260.......270.......280.......290.......300

Gaac_spg1 GACAACCCAGCGATGGAT---TCTGGCATTCTGCCTTTCACTG---GCCTCAGGTATTTCCACCGCAAAATGATGAAGATGCAATATTTAAACTCTTGCCATTTTGAAAATGATTTGTCTTTTTTTGTGATGTTTTCACAAGATGAGTAG  327
Gaac_spg2 GCCAAAACAGCGATGGAT---TCTGGCATTCTGCCTTTCACTG---GCCTCAGGTATTTCCACCACAAAATGATGAGATA-CAGTATTTAAACTCTTGCCATTCTGATAATTATTTTTTGTTTTACCTAATGTTTCACTAGATGAGCAGT  145
Gaac_spg3 GACAACCCAAAGACGGAT---TCTGGCATTCTCTCTTTCACTG---GCCTCAGGTATTTCCACCGCAAAATGATGAATGGGCAAAATTTAACTCTTGCCAGTCTGAAAATGATTTGTTGTTTTTCGTGATGTTTTCACTGGATGAGTAA  146
Gaac_spg4 GCAATTTGATCATTGGGTGAGTTTTGCATCAAGAGTTTTGAAAAATGGCTCTGAAATTAGCG--GCAAAGTGATTGGAGGCC----------------------------------------------------------------------   82
Gaac_spg5 GACAACCCAGCGATGGAT---TCTGGCATTCTGCCTTTCACTG---GCCTCAGGTATTTCCACCGCAAAATGATGAAGATGCAATATTTAAACTCTTGCCATTTTGAAAATGATTTGTCTTTTTTCGTGATGTTTTCACAAGATGAGTAG  444
Gaac_spg6 ------------------------------------------------------------------------------------------------------------------------------------------------------
Gaac_spg7 GACAACCCAGCGATGGAT---TCTTGTATTCATCTTTTCACTG---GCCTCAGGTATTTCAACCGCAAAATTATGGAAATCAATATTATATATGAATATAATATTTTATGTCTTGCCAGTTCATAAACTTAGATTTAGTTTTTTTGTCAG  236
          .......310.......320.......330.......340.......350.......360.......370.......380.......390.......400.......410.......420.......430.......440.......450

Gaac_spg1 GTAGAAGAAAGATGTATAATTTTTGCAGCTTAATATTTCAATCTCATGCTACCTTATATCTATACTATACTATAAAGTTTTGTTAGCTTCCGCGATTAGTTCCATGTCGAAGTGTCCCTGAGCAAGACACCTAACCCCTAATTGCTCCCC  477
Gaac_spg2 TAGANNNNNNNNNNNNNNNNNNNNNNNNNNNNNNNNNNNNNNNNNNNNNNNNNNNNNNNNNNNNNNNNNNNNNNNNNNNNNNNNNNNNNNNNNNNNNNNNNNNNGTTTCAACAAAAAAAGAAAGAAAAAAGAATTTAAACCCCGGCCATT  295
Gaac_spg3 GTTGAATAAAGATGTATAATTTTT------------------------------------------------------------------------------------------------------------------------------  170
Gaac_spg4 ------------------------------------------------------------------------------------------------------------------------------------------------------   82
Gaac_spg5 GTAGAAGAAAGATGTATAATTTTTGCAGCTTAATATTTCAATCTCATGCTACCTTATATCTATACTATACTATAAAGTTTTGTTAGCTTCCGCGATAAGTTCCATGTCGAAGTGTCCCTGAGCAAGACACCTAACCCCTAATTGCTCCCC  594
Gaac_spg6 ------------------------------------------------------------------------------------------------------------------------------------------------------
Gaac_spg7 GTTTCCACTAGATGAGTATAATTACCATAATAAGTAATTCTCATGCTACTTATATTTACAATACTATCAAAAATACAATTTTAATCCAAAAATGTATTCATTAACTTCTGTGACTGGTTACATCTACATCTAAAATATGGT---------  377
          .......460.......470.......480.......490.......500.......510.......520.......530.......540.......550.......560.......570.......580.......590.......600

Gaac_spg1 AGGCAAAAATGTGAAAAGCCATGGATTTAAAGTGTAATGTAAGTCGCTTTGGATAAAAGCGTCTGCTAAATGACCTGTAATGTAATGTAATGTAGTTACCATACCAATCAAAAATACGAACACAAAACATATCCTAAACATAAATAGAAT  627
Gaac_spg2 GTGCGAATATAATAAAATTTACCCGAGAGTTTGCGAGTAAAAGAGCAGGTAGAAAAAAAGTTTTTCATGCACCCTCCTGGCAGATTTTTCAATTCTCGTCACCTTTTTATTTGTTCTGTGTTTCAAAGATTTCTTCTGTTATTTTTGTTG  445
Gaac_spg3 -----GCAACCTTATAGGCTATATATATATATATATATATATATATATATATATATATATATATATATATATACATACTATATCATCAAAAATTTTTGTTA-GCTAATCTAAAATGTGCACACAGAAAATAT---GAATA-AAATATGAT  310
Gaac_spg4 ------------------------------------------------------------------------------------------------------------------------------------------------------   82
Gaac_spg5 AGGCAAAAATGTGAAAAGCCATGGGTTTAAAGTGTAATGTAAGTCGTTTTGGATAAAAGCGTCTGCTAAATGACCTGTAATGTAATGTAATGTAGTTACCATACCAATCAAAAATATGAACACAAAACATATCCTAAACATAAATAGAAT  744
Gaac_spg6 ------------------------------------------------------------------------------------------------------------------------------------------------------
Gaac_spg7 -----------------------------------------------------------------------------------------------------------------------CACAAAACATATGATGGCCTTAAAAAAAT  407
          .......610.......620.......630.......640.......650.......660.......670.......680.......690.......700.......710.......720.......730.......740.......750

Gaac_spg1 AAGATGCACTGTTGAAGAATGAATAGTTGACACTGACAAAAGCAATTCCTTCCAATTATCAACATATAAACCTTCGGTACTTTAAAATAGAGGTAGCTGCAATTTGTTGTCTCTGCTTACACGTATGAATACTTTATCTGGTATAACAAG  777
Gaac_spg2 AGTAGTTACACACCAATCTGAAATATGAAAATGAAACCTCATAAACATGTGAATAAAATATG--ATAGAA--TGTTGTAGAATAAAATAGTTGATACTGACAAAAACCTTTT------------------------------------  553
Gaac_spg3 TAACTTCCTT-----AGAATAAATAGTGGACACTGACAAAAGCAATTTCTTCCAATTATGAACATTTGAACTTTTGGTCATCTAAAATAGAGGTAGCTGCGATTTGTAGTTGCTGCTTACACATATAAATACTTTA-CTGGTAAAACAGT  454
Gaac_spg4 ------------------------------------------------------------------------------------------------------------------------------------------------------   82
Gaac_spg5 AAGATGCACTGTTGAAGAATGAATAGTTGACACTGACAAAAGCAATTCCTTCCAATTATCAACATATAAACCTTCGGTACTTTAAAATAGAGGTAGCTGCAATTTGTTGTCTCTGCTTACACGTATGAATACTTTATCTGGTATAACAAG  894
Gaac_spg6 ------------------------------------------------------------------------------------------------------------------------------------------------------
Gaac_spg7 GATGACACTATTAT-AGATTAAATCGTTGACACTGACAAGAGAAATCTCTCCAAATTCTGAGTATTTTTACTTTTTAAGTTGAAGTAGATAATCATTTTAATTAGTAGACATTTTCAAACTAGCACTTGAATGTTTTTTAGTATTATTCA  556
          .......760.......770.......780.......790.......800.......810.......820.......830.......840.......850.......860.......870.......880.......890.......900
```

2

```
Gaac_spg1 TATT-TTCTTTTCAATCCATACATTTCCC---TCATAATTCATGTTTACTTGCACCCTGACACTGTTTTTTTCTTCTAG-TTTTTGGCACAGTGGAATTGTTCAAGACCAAAGAAATTCAGACATGTGAGTAAAAAACGGATCAATATGT  922
Gaac_spg2 --------------------------------------GTACTATAGAGTGAACCATGACATTGTATTTTTCTTCTAGTTTTTTGGCACAGAGGAATTGTTCAAGACCAAGATAAATTCTGACTTGTGAGTACAAACCAAACAATGTGT  664
Gaac_spg3 AAGAATTTTTTTTTATTTAGAAATTTCTA---TGATAATTAATGTTTACTTGCATAATGACACTGTATTTTTCTTCTAG-TTTTTGGCACAGTGGAATTGTTAAAGACCAAAGAAACTCAGACTTGTGAGTACAAA-CCAATCAATGTGT  599
Gaac_spg4 ------------------TAAATTTCCC---TCATAATTAATGTTTACTTACATAATGTCACTGTATTTTTCTTCTAG-TTTTTGGCACAGTGAAATTGTTCAAGACCAACGAAACTCAGACTTGTGAGTACAAATCAATCATGTATAT  209
Gaac_spg5 TATT-TTCTTTTCAATCCATACATTTCCC---TCATAATTCATGTTTACTTGCACCCTGACACTGTTTTTTTCTTCTAG-TTTTTGGCACAGTGGAATTGTTCAAGACCAAAGAAATTCAGACTTGTGAGTAAAAAACCGATCAATATGT 1039
Gaac_spg6 --------------------------------------------------------------------------------------------------------------------------------CTTCTTCTAGTTTTTGGCACAG   22
Gaac_spg7 TAATTCATAATATTATTCATAATTAGTATTTTTCATAATTCATGTTTACTTTCACCATGACAGTGTATTTTTCTTCTAG-TTCTTGGTACAGTGGAATTGTTCAAGACCAAAGAAGCTCAGACATGTGAGTACAAACCGATGTATGGTAT  705
          .......910.......920.......930.......940.......950.......960.......970.......980.......990......1000......1010......1020......1030......1040......1050

               *                                                                        *          *                *
Gaac_spg1 GATATTCATCAGTT------CACTACCCAGATTACTTTTTTGAAATGATTGTATATG--TTGTAGGTACTAGCTACACTG--TTTCAGGTTTTTT-AAACACATGCTGTTTCATTGTTTGGATGTTGCATTTCTCTCATTTATTGTCTTA 1061
Gaac_spg2 GATATTTATCAGTTCACTACAATGATTTCTTTTTTGACATTATTGTATATGATATAGGTACTAGCTACACTGTTTCAGGTTTTTAAAACACATGCTGTTTCATTGTTTGGATGTTGTCGTGGCGCAGGGGTTAGAGAAGGTGTGCTGGG  814
Gaac_spg3 GATATTTATCATTTT-----CACTACCCAGATTACTTTTTTACA----TTAT---TA--TCGTAGGTACTGGCTAGATTG--TTTCAGGTTTTCTGAAACATATGCAGTTTCATTATTTGGCTGTT-----------------TGTCTTA  716
Gaac_spg4 AATCATTATATTTATCAGTTCACTACCCAGATTACTTTTTCACATTATTGTATTATATGTCGTAAGTACTGACTACTATTATTTCAGGTTTTCACTAATTAATT-------------------------------------------CTTA  318
Gaac_spg5 GATATTCATCAGTT------CACTACCCAGATTACTTTTTTGAAATGATTGTATATG--TTGTAGGTACTAGCTACACTG--TTTCAGGTTTTTT-AAACACATGCTGTTTCATTGTTTGGATGTTGCATTTCTCTCATTTATTGTCTTA 1178
Gaac_spg6 TGGAATTGTTCAAGACCAAGGAAATTCTGACTTGTGAGTACAAACCAATCAATGTGTTATACTAAGTACTGGCTACATTG--TTTTAGGTTTTATGAAACACATGCTGTTTCATTGTTTGGCTCTTGCATTTCTCTCATTTATTGTCTTA  170
Gaac_spg7 TCATCAGCTCAAAACACAAATTACTTAGCATCATTATTTAGCATCATTGCATATGTTGTAAGTACTGGATATATTGTTTTCAGGTTTTTTAAACACATGCGGTTTTATTATTTTGCTACTGTATTTCATTTCTATGAAATGATTTATTG  855
          ......1060......1070......1080......1090......1100......1110......1120......1130......1140......1150......1160......1170......1180......1190......1200

                   *  *                     *       *
Gaac_spg1 CTGTTCCTTTTGTGATCACAATT-------TTGCAAGTTGCTTTTT-------------------------------------------------------------------------------------------------------- 1100
Gaac_spg2 CAAGCACAAGGTTGGTGGTTTGATTCCAGGCTGCCCCTTGTTCCATGTCGAAGTGTCCCTGAGCAAGACACCTAACCCCTAATTGCTCCCTGGGCAAAAATGTGAAAAAAGCCATGGGTTTAAAGTGTAATGTAAGTCGCTTTGGATAAA  964
Gaac_spg3 CTGTTCCTTTTGTGATCACATTT-------TTGCTGTTTGCTTTTT--------------------------------------------------------------------------------------------------------  755
Gaac_spg4 AAGTTCCTTTTGTGATCACATTT-------TTGCTATTTGCTTTTTGCAATACAAACCAAGACTCAAAATTGCCTGTAGGTATGTGAATGTGAGTGTGAATGGTTGTATGTCTCTGTGTTAGCCCTGCGATTGGCTAGCGTCCGGGATGT  461
Gaac_spg5 CTGTTCCTTTTGTGATCACAATT-------TTGCAAGTTGCTTTTT-------------------------------------------------------------------------------------------------------- 1217
Gaac_spg6 CTGTTCCTTTTGTGATCACATTTTTGCTAGTTGCTAGTTGCTTTTTGCAAAACAAACCAAACAAAAAGCAACTAGAACTGATCGTTGCAATGTGTAGGGTTATATGG-------------------------------------------  277
Gaac_spg7 TCCTATTGTTCCTTCTGTGGTCA-------CATTAAATGTGAAAATGTGAAGAACATTACAAAATTGATTTCTTTTTTAT----------------------------------------------------------------------  928
          ......1210......1220......1230......1240......1250......1260......1270......1280......1290......1300......1310......1320......1330......1340......1350

Gaac_spg1 ------------------------------------------------------------------------------------GCAAAACAAACCAAACAAACAAAATCCACTGACACTCACAATGTGTAGGATTCTGTACATGGTA 1164
Gaac_spg2 AGCGTCTGCTAAATAACCTGTAATGTAATGTAATGTTGCATTTCTCTCATTTATTGTCTTACCTTTTGTGATCACATTTTTGCCAGTTACTATTTGCAAAATAAACCAAACAAATTCCACTGCAGTGAGTAG-GGTTT--GTACGTGGCA 1111
Gaac_spg3 ------------------------------------------------------------------------------------GCAAAACAAACCAAACAAAT----TCCACTGGCTGTTGCAATGTGTAGGATTGTGTACATGGAA  815
Gaac_spg4 ACCCTGGTCTCTCGCTAGTTAGGTGGGATAAACAGCAGCCCCCTGCGACCCTATGTGCAGGATAAGCGGTTTGACAATGAATGGATGGAAAACAAACCAAACAAACAAATTCCACTGACCATTGCAATGTGTAGGGTTGTGTATATCGGA  611
Gaac_spg5 ------------------------------------------------------------------------------------GCAAAACAAACCAAACAAACAAAATCCACTGACACTCACAATGTGTAGGATTCTGTACATGGCA 1281
Gaac_spg6 ----------------------------------------------------------------------------------------------------------------------------------------------ACATGGTA  285
Gaac_spg7 ------------------------------------------------------------------------------------------------------------------------------------------------------  928
          ......1360......1370......1380......1390......1400......1410......1420......1430......1440......1450......1460......1470......1480......1490......1500

Gaac_spg1 GATAAACA---TAATTTAGACGT---GAATTTAGACGT------GAGTT-GAGCATTTGGCTTATTGAGTTGAAAAGA--CACTCTAAAAAATCGTATTTTTCATTCAATGATTGTATTCCTTAGACTGAGTAAGGAAAATCGAGTACAC 1299
Gaac_spg2 GATAAACACAATTTGGGATATGC---TGTTTGGACTTGAAATAAAAGTT-GATCATTTGGCTTAATCAGTTAAAAAGA------TATAATATTTATCGTTCAATGAAATGAATGGTATTCTTTACACTAGGTAAGTAAAATCGAATACAT 1251
Gaac_spg3 GATAAACA---CAATT-----GT---TGTTTAGAATTA------GAGTT-GAGCATTTGGCTTATTGAGTTGAAAAGA--CACTCTTAAAAATC---CTTTTCATTCAATGATAGTTTTCCTTACACTAGGTAAGGAAAATCGAGCACAC  942
Gaac_spg4 TAATAACGGTTTAGGTATGTTGTTTAGAATTGAAATAA------AAGTTTAAGCATTTGGCTTATTTATTTAAAAAAAGGCAGTCTTAAAAATCATATTCGGAATTCAATGATAGTATTCCTTGCACTGGGAAAGGAAAATCGAATACAC  755
Gaac_spg5 GATAAACA---TAATTTAGACGT---GAATTTAGACGT------GAGTT-GAGCATTTGGCTTATTGAGTTGAAAAGA--CACTCTAAAAAATCGTATTTTTCATTCAATGATTGTATTCCTTAGACTGAGTAAGGAAAATCGAGTACAC 1416
Gaac_spg6 GATAAACA---TAATTTCGCATTTGCTGTTTAGACGTGAAATAAGAGTT-GAGCATTTGGCTTATTGCGTTGAAAAGA--CACTGTGAAAAATTATATTTTTCATTCAATGGTAGTATTCCTTACACTGGGTAAGGAAGATCGAGTACAG  429
Gaac_spg7 ------------------------------------------------------------------------------------------------------------------------------------------------------  928
          ......1510......1520......1530......1540......1550......1560......1570......1580......1590......1600......1610......1620......1630......1640......1650
```

3

```
                                                                                                 *** ******  * * * ***   *  ** ************** *********** ********
Gaac_spg1 TTTAACCCAAACATTTAAATCTAAAGTC--------------ATTTCATTTACATATTTCTTAATTTCTGACCTCACATTTCTCCTTATTGTTCAACCTTAATTGTTGAATTGTGATCAGACACTTGCAGGACATTTGGCAGCGGGATCG 1435
Gaac_spg2 GTTGACCATAATAATTAAATCTTAAT-CCAATATTATAAAATAAATGTATTTACTTAATCTCAATTTCTGACCTCACATTTGTCCTTATTGTCTAGCATTAATCCCTCAATTGTGATCAGACACTTAAAGGACATTTGGGAGCGGGATCA 1400
Gaac_spg3 CTTGACCCTAACATTAAAATCTAAATTCCACATTACAAAGTAATTTAATTTACTTATTTCTTCATTTCTGATC-CACATTTGTCCCTATTGTTTAACATAAATTGTTGAATTGTGATCAGACACTTGCAGGACATTTGGCAGCGGGATCG 1091
Gaac_spg4 GTTGACCATAACATTCAA-----------------------------------------------------------GTCCTTATTGTCTAACTTTAATCCCTCAATTGTGATCAGACACTTGCAGGACATTTGGCAGCGGGATCG  842
Gaac_spg5 TTTGACCCAAACATTTAAATCTAAAGTC--------------ATTTCATTTACATATTTCTTAATTTCTGACCTCACATTTCTCCTTATTGTTCAACCTTAATTGTTGAATTGTGATCAGACACTTGCAGGACATTTGGCAGCGGGATCG 1552
Gaac_spg6 ATTGACCCAAACATTTAAATCAAAATTC-------CAAAGTAATTTCATTTACCTATTTCTTAATTTCTGACCTCACATTTGTCCTTATTGTTTAACATTAATCGTTGAATAGTGATCAGACACTTGCAGGACATTTGGCAGCGGGATCG  572
Gaac_spg7 ----------------------------------------------------------------TTTCTGACCCCACATATGTCCATATTGTCTAGCATTAATTGTTACATTGTGATCAGACACTTGCAGGACATTTGGCAGCGGGATTG 1014
          ......1660......1670......1680......1690......1700......1710......1720......1730......1740......1750......1760......1770......1780......1790......1800

          *********** ************* ***** ***** ******* *****  **** ********  ******    ***************************** *** ***************** ********* * ** ****
Gaac_spg1 TCCAGCCTTTTAAGGGGGAGAGTTACTATGTTCGGTCCGACTGCCCGTTCAAACTCACAAGCTTCAACGTCAACCGGGGGGAATATTCTGTCACCATACGGCGAGGTCACAACGGGCTGTTGGTCCAAGTCGAGATCATCGTCAACAAAG 1585
Gaac_spg2 TCCAGCCTTTTAAGGGGGAGAGTTACTATGTTCGGTCCGACTGCCCGTTCA--CTCACAAGCTTCACCGTCAACCGGGGGGAATATTCTGTCACCATACGGCGAGGTCACAACAGGCTGTTGGTCCAAGTCGAGATCATCATCAACAAAG 1548
Gaac_spg3 TCCAGCCTTTTCAGGGGGAGAGTTATTATGTTCGGTCTGACTGCCCGTTCACACTCACAAGCTTCAACGTCAACCAGGGGGAATATTCTGTCACCATACGGCGAGGTCCCAACGGGCTGTTGGTCCAAGTCGAGATCATCGTCAACAAAG 1241
Gaac_spg4 TCCAGCCTTTTAAGGGGGAGAGTTACTATGTTCGGTCCGACTGCCAGTTCATACTCATAAGCTTCACTGTCAACGGGAAGGAATATTCTGTCACCATACGGCGAGGTCACAACGGGCTGTTGGTCCAAGTCGAGATCATCATCAACAAAG  992
Gaac_spg5 TCCAGCCTTTTAAGGGGGAGAGTTACTATGTTCGGTCCGACTGCCCGTTCAAACTCACAAGCTTCAACGTCAACCGGGGGGAATATTCTGTCACCATACGGCGAGGTCACAACGGGCTGTTGGTCCAAGTCGAGATCATCGTCAACAAAG 1702
Gaac_spg6 TCCAGCCTTTTAAGGGGGAGAGTTACTATGTTCGGTCCGACTGCCCGTTCAAACTCACAAGCTTCAACGTCAACCGCGGGGAATATTCTGTCACCATACGGCGAGGTCATAACGGGCTGTTGGTCCAAGTTGAGATCATCATTAACAAAG  722
Gaac_spg7 TCCAGCCTTTTAAGGGGGAGAGTTACTATGTCCGGTCCGACTGCCCGTTCACACTCACAAGCTTCACCGTCAACCGGGGGGAATATTCTGTCACCATACGGCGAGGTCGCAACGGGCTGTTGGTCCAAGTCGAGATCATCATCAATAAAG 1164
          ......1810......1820......1830......1840......1850......1860......1870......1880......1890......1900......1910......1920......1930......1940......1950
```

3

```
                  ***  ** ******* * ***** ** *  * *** ******* * ********    *                                            **    *
Gaac_spg1 TCACAACACTTTTGCAGAATGGCCACATCCTAGTGCAGAACAACAGGTTTGAGCCCAAGCAGAAAACTGCAGCCTCACTGAACAACAAATAATGAGTAATGCGAAAAA-GTCAATTTAAAAATTCATCACATGACTGTTCTTCCAAAACA 1734
Gaac_spg2 TCACAACACTTTTGCGGAATGGCCACATCCTGGTGCAGAACAAAAGGTTTGAGCCCAGAGCTGAAAACTGCAGCCACACTGAACACCAAGGAATGAGTAATGCAAAAAA-GTATCTTTAATCATACATCACATGACTGTTCTTCTAAAACA 1697
Gaac_spg3 TCACAACACTTTTGCAGGATGGCAACGTTGTGGTGAAGAACAAAAGTTTGAGCCCAAGCAGAAAACTGCAGCCACACTGAACACCAAGTAAAGACTAATGCAAAAAAAGTCAATTTTGTTAAATGAAAACATACTGAGTTAACACACCA 1391
Gaac_spg4 TCACGACACTTTTGCAGAATGGCCACGTTGTGGTGAAGAACAAAAGGTTTGAGCTCAGGGAGAAAACTGGTTTGGGTGACC---------GCCACCAATGGAGAAGACAG---------------------------------------- 1093
Gaac_spg5 TCACAACACTTTTGCAGAATGGCCACATCCTAGTGCAGAACAACAGGTTTGAGCCCAAGCAGAAAACTGCAGCCTCACTGAACAACAAATAATGAGTAATGCGAAAAA-GTCAATTTAAAAATTCATCACATGACTGTTCTTCCAAAACA 1851
Gaac_spg6 TCACGACACTTTTGCGGAATGGCCACATCCTGGTGCAGAACAAAAGGTTTGAGC-AATGCA---------------------------------------------AAATA-GACTATTTAATCATTCATCACATGACTTTTTCCAAAACGGA 828
Gaac_spg7 TCATGACCCTTTTGCAGAATGGCCACGTTGTGGTGCAGAACAAAAGGTTTGAGCCCAAGCAGAAAACTGCAGCCACACTGAACACCAAGGAATGAGTAATGGAAAAAA-GTCTATTTAATCATTCATCACATGACGGTTTTTCCAAAACT 1313
          ......1960......1970......1980......1990......2000......2010......2020......2030......2040......2050......2060......2070......2080......2090......2100

                                                                                                                                                   *      *  **
Gaac_spg1 -GACAACTATTTTAATTAAATGGACATT---CACAACACTTTAAAAAAAATGTTCACCTGCCTAATGCCACCAATCCCAGGTGATTATTTGCTTTGAGTCAGGGACATTGTATTATCAGTGTTTTGTGAAATTAAAACGTACTGAGTAA 1880
Gaac_spg2 ----AGCG--CTCAATTAAATGGACATTTCACCCATTATTTTT-----------ACCTGCCTAATGCCACCAATCCCAGGTAATTAATAGCTTTGAGTCTAGAATACTGTATTATCAGTGTTTTATAAAATGAAAACATACTGAGTAA 1830
Gaac_spg3 AGTCAAGC--TGTTACCAAGTTATCACTTAGGGACTTATTTCAGAGAGGTGGA--AACTGTAATAGTTCACCCATTCGGTCTTTAATTAAAAAAAAAAGTTGAGTAAGAAAAACTATCCAAAGTAAAAGCAAGCCTTTTTTTAATGTCAA 1537
Gaac_spg4 -----------------------------------------------------------------------------------------------------------------CGACTAAAGTAAAAACAAACACCTGTTTTATGTCAA 1129
Gaac_spg5 -GACAACTATTTTAATTAAATGGACATT---CACAACACTTTAAAAAAAATGTTCACCTGCCTAATGCCACCAATCCCAGGTGATTATTTGCTTTGAGTCAGGGACATTGTATTATCAGTGTTTTGTGAAATTAAAACGTACTGAGTAA 1997
Gaac_spg6 CAGCTCTTAAAATTAGATAGACATTTCA-----CAACGCTTTATTTTTTTT---CATTT---------CACCAATCCCATGTGATTATTAGCTTTGAGTCTGGGATACTGTATTATCAGTGTTTTG------TAAAA-----TGAGTAA 950
Gaac_spg7 CCTTCGATAAAATGTATACTACACAAGACATTTTTTAATTTTGCATGCTTAATGTT------------ACCAATGCTAGGTGATTATTAGCTTTCAGTCTGGGAAACTGTATTGGCAGTATTTTGTGAAATGAAAACATACTGAGGTAA 1450
          ......2110......2120......2130......2140......2150......2160......2170......2180......2190......2200......2210......2220......2230......2240......2250

                *      *
Gaac_spg1 CATACTGAGTCAAGATGTTACCGAGTTATCACATAGGGACTTATT--------------------------------------------------------------------------------------------------------- 1925
Gaac_spg2 CACACTGAGTCAAG-------------------------------------------------------------------------------------------------------------------------------------- 1844
Gaac_spg3 AT-GGTAGTCATAATTTGT-AAAAAGACCTTTAATGTATTTGATGTTAGAACCCATTTTCT--------GTATTGTTTTCCAATGGTCCATTGTTACTTGGTACATTTTTTCAACTATTGGCGTACTTCCTCTACTACTCATCATCTAGC 1677
Gaac_spg4 GT-GGTGGTCACAATTGGTGAAAAAGACTATTAATGTATTCAATGTTAAAACGCATTTTCTCTGTATCTGTATTTTTATCCAATGGTCCATTGTTACTAGGTACATGTATTTAACTATTGGACCACATCCTCCACAACTCTTCATCTAGC 1278
Gaac_spg5 CATACTGAGTCAAGATGTTACCGAGTTATCACATAGGGACTTATT--------------------------------------------------------------------------------------------------------- 2042
Gaac_spg6 TACACTGAGTCAATCTGTTACCGAGTTATCACGTAGGGACTTATT--------------------------------------------------------------------------------------------------------- 995
Gaac_spg7 TATACTGACTCAAGCTGTTACCGAGTTATCACATATGGACTTTCCATTTTTTCCATGAGGTGGAATCTAATAGTTCAACCATTCCATCATTATTTTCAAAAAAAGTTAAACGAGTGAGAACACTCTGTCCAAAGA-------------- 1585
          ......2260......2270......2280......2290......2300......2310......2320......2330......2340......2350......2360......2370......2380......2390......2400

Gaac_spg1 ------------------------------------------------------------------------------------------------------------------------------------------------------ 1925
Gaac_spg2 ------------------------------------------------------------------------------------------------------------------------------------------------------ 1844
Gaac_spg3 AAATATGCACATTTTACACAACTACAGTTATTTGAAAACTTCAGTTCCTAGTTTGTGG-ACATTTTTGGACACACAGCATTTGGAAATAAGAGTAGCATGTTGATTAGCTGAGGACATTCCACTCCGACTCTAGCTCAAATAGGGACATT 1826
Gaac_spg4 AAAAACGCACATTTTACACAACTACAATTATTTGAAAACTTCAGTTCCTAGTTTTTTTAGATTTTTAGACACACAGCATTTGGAAATTAAGAGTAGCATGTTGATTAGCTGAGGAATAAAAGACATTTTTCTTCTTCCTTTAGATGTATT 1428
Gaac_spg5 ------------------------------------------------------------------------------------------------------------------------------------------------------ 2042
Gaac_spg6 ------------------------------------------------------------------------------------------------------------------------------------------------------ 995
Gaac_spg7 ------------------------------------------------------------------------------------------------------------------------------------------------------ 1585
          ......2410......2420......2430......2440......2450......2460......2470......2480......2490......2500......2510......2520......2530......2540......2550

                                                              *                                  * *             *        *        *
Gaac_spg1 --TCCGGGAGGTGGAAACTGTAATAGTTCACCCATTCTGTCTTTAATTAAAAA-CAAACACCTGTTTTATGTCAAGTGGTGATCGTAATTGGTCAAAAGAATTTATTTGTTGATAAAATCCATTTTCTCTGTATTGTATTGTTTTCCGA 2072
Gaac_spg2 ---------------CAAAAGTTGAACCACTGAGAAGACTGTTACACCTATT-TTACGTCAAGTGCTGGTCATAATTGGGCAAGCTTTTAATTAATTTATTTGTTGATAAAATCAATTTCCCCTGCATCTGTATTGTTATCCAA 1978
Gaac_spg3 GGTCTTTAAAAAAAAAAGTTGTGATACATTACATAGATTGGATTG-TTCTGCA-TTGTTTACGTTTCAAACTT----------------CATAGTACTGACATACTTTTACTTAAATGCCATTTTTATTGCAGATTT----------A 1947
Gaac_spg4 -------------AAACGTTGTGATACATTACATTGATTGGATTT-TTCAGCA-GTGTTTGCTTTTAAACTTGAATTGAAATATATCCTCATTGTACTCCAATACTTTTACTTATATGCAATTTTCACTGCAGGATTTTTAAAACAGTA 1563
Gaac_spg5 --TCCGGGAGGTGGAAACTGTAATAGTTCACCCATTCTGTCTTTAATTAAAAA-CAAACACCTGTTTTATGTCAAGTGGTGATCGTAATTGGTCAAAAGAATTTATTTGTTGATAAAATCCATTTTCTCTGTATTGTATTGTTTTCCGA 2189
Gaac_spg6 --TCAGGGAGGTGGAAACTGTAATAGTTCACCTGTTCTGTCTTTAATTAAAAAACAAAATACCTGTTTTGTCAAGTGGTGGTCATGATTGGTCAAAAGAATTTATTTGTTGATAAAATCTATTTTCTCTGTATTGTATTTTTATCCAA 1143
Gaac_spg7 -----------------------------------------------AAAAA-CAAACACCTGTTTCATGTCAAGTGGTGGTCATAATTCTCTAGGACTTTTAAATGTATTTGTTGTTATAACCTATTTCTCTGTGTCTGTATTGTTAT 1686
          ......2560......2570......2580......2590......2600......2610......2620......2630......2640......2650......2660......2670......2680......2690......2700

                       *
Gaac_spg1 TGGTCCATTAAT---TGGTACATTTATTGAAATATTGGATAACTTACTCCACTCC---------TCTT------------------------------------------------------------------------------------ 2128
Gaac_spg2 TGATCTATTGTTACTAGGTACATTTATTCAACTATTATTTCCCCTACTACTCATCTGGCAAAAATGCA--------------------------------------------------------------------------------- 2046
Gaac_spg3 TT---GCAATGT---AG-TACTTG-TACTTCTACTACTACCCCTAGTATTTAATAACAGTCGATGTTTA------------------------------------------------------------------------------- 2007
Gaac_spg4 TTTTAACAATGT---AG-TACTTG-TACTTCTCTAGTACACCAAATATTTAATAACAGTCGATGTTTA------------------------------------------------------------------------------- 1626
Gaac_spg5 TGGTCCATTAAT---TGGTACATTTATTGAAATATTGGATAACTTACTCCACTCC---------TCTT------------------------------------------------------------------------------------ 2245
Gaac_spg6 TGGTCCATTTTT---TGGATTACTTCCTCACCTCTTCTCTTCTGGCAAAAATGCACATTTTACACAACTACAATTATTTTAAAACTTCAGCTAGTTTGTGAAGATTTTTGCACAATTATTTTGTCTTAAAACTAAGAGTAGCATGTTGA 1290
Gaac_spg7 CCAAAGGTCTATTGACACGAGGTACATTTATATAATTATCGGACCACATCCTTTACTACTCTTCATCTGGGAAAATTGCGCATTTTACACAACAATAATTATTTGAAAAGTTCAGTTCCTAGTTTGTTGTTCCTAGATTTTTGGACTCAC 1836
          ......2710......2720......2730......2740......2750......2760......2770......2780......2790......2800......2810......2820......2830......2840......2850

Gaac_spg1 ------------------------------------------------------------------------------------------------------------------------------------------------------ 2128
Gaac_spg2 ------------------------------------------------------------------------------------------------------------------------------------------------------ 2046
Gaac_spg3 ------------------------------------------------------------------------------------------------------------------------------------------------------ 2007
Gaac_spg4 ------------------------------------------------------------------------------------------------------------------------------------------------------ 1626
Gaac_spg5 ------------------------------------------------------------------------------------------------------------------------------------------------------ 2245
Gaac_spg6 TTAGCTGAGGACATTCCACTCCGATGCTAGCTAAAATAAGAAATTTCTCTTTACTTCCTTTAGATGTTTAAAAGTTGTGATACATTATATTTATTGGATTGTTCAGCATTGTTTACTTCACTATAGTACTGACATACTTTTACTTAGATG 1440
Gaac_spg7 AGCATTTGAAAATAAGACTAGCATGTTGATTAGCTGAGGACATTCCACTCCAATTCTAGTGAAGATAAGAAATGTCTTCTTCCTTTAGATTTTATATATATATATATATATATATATATATATATATATATATATATATTATTGATTGAATT 1986
          ......2860......2870......2880......2890......2900......2910......2920......2930......2940......2950......2960......2970......2980......2990......3000

                                                                                                                                                 * **
Gaac_spg1 -------------------------------------------------------------------------------------------------------------------------------------------TGTCTTG 2135
Gaac_spg2 -------------------------------------------------------------------------------------------------------------------------------------------CATTTA 2053
Gaac_spg3 -------------------------------------------------------------------------------------------------------------------------------------------TGTCTTG 2014
Gaac_spg4 -------------------------------------------------------------------------------------------------------------------------------------------TGTCTTG 1633
Gaac_spg5 -------------------------------------------------------------------------------------------------------------------------------------------CGTCTTG 2252
Gaac_spg6 CCATTTCACTGCAGGATTTTAAGAATAGTTTTTTAACCATGTAGTACCTGTACTTCCCTAGTAACCCAGATATTAATTAACAGTTGATTTA---------------------------------------------------- 1540
Gaac_spg7 GTTCAGTATTGTTTACTTTAAAACTTCCTCATTGTACTGACATACTTTTACTTAAATGCAATTTTAGATTGTAAATGTATTATTTAACAATGTAGTACCTGTACTTCCCTAGTAACCCAAATATTTATTAACAGTTGATTTATGTCTTG 2136
          ......3010......3020......3030......3040......3050......3060......3070......3080......3090......3100......3110......3120......3130......3140......3150
```

4

```
              *      *     **   *           *      **              *     *   *  *  *  *             *  ***          *          **       ***   *   *  ** *        *
Gaac_spg1 ATAG-GTTTCACTTCCATACGACCACACCTACCAGCATATCTTTAAATACGGCATCTACACTAGACTGAGGAGCTCGCTGCTTCCTTTTACTGTCACCTGGCACAATGTACATGGGGAATAAACTCTCTGTGGGTAAGTTGACGTTATA 2284
Gaac_spg2 CACTACTAGAATTAATGAAACTCTCACTCCAGTTAGTTTTTGGGGATTTTGGAGACACAGAAGAGTAGCACGTTGATTAGCTGAGGACATTCCACTCCAATTCTAGCTAAATAAAGACATTTTTCTTCTTTCTTTAGATCAGCGATTC 2203
Gaac_spg3 ATAGTGTTTCCGATCATCGCACCAAAAATACCAGCAAATCTTTAAATACAGCATCTACACTAGACTGAGGAGCTCGCTGCTTCTTTTAGTGTCACCTGGCAGCAGTGTATGTGGGGAATAAACTCTCTGTGGGTAAGTTGGCGTTATA 2164
Gaac_spg4 ATAGGGTAACCCATCCATGCAACCGAAAATACCAGCATATCTTTAAATACGGCATCTACAGTAGACTGAGGAGCTCGCTGCTTCCTTTTACTGTCACCTGGCACAATGTACATGGGGAATAAACTCTCTGTGGGTAAGTTGACGTTATA 1783
Gaac_spg5 ATAG-GTTTCACTTCCATACGACCACACCTACCAGCATATCTTTAAATACGGCATCTACACTAGACTGAGGAGCTCGCTGCTTCCTTTTACTGTCACCTGGCACAATGTACATGGGGAATAAACTCTCTGTGGGTAAGTTGACGTTATA 2401
Gaac_spg6 ATAGTGTTTCCCTTCCATACGACCACACCTACGAGCATATCTTTAAATACGGCATCTACACTAGACTGAGGAGCTCGCTACTTCCTTTTACTGTCACCTGGCACAATGTACATGGGGAATAAACTCTCTGTGGGTAAGTTGACGTTATA 1690
Gaac_spg7 ATAGTGTTTCCCTTCCATACGACCACACCTACCAGCATATTGTTAAATACGGCATCTACACAAACTGAGGAGCTCGCTGCTTCCGTTTACTGTCACCTGGCACAATGTAGACGGGGAATAAACTCTCTGTGGGTAAGTTGACGTTATA 2286
          ......3160......3170......3180......3190......3200......3210......3220......3230......3240......3250......3260......3270......3280......3290......3300

          *     *  *    *          *  ***        *   ***          *   * **   **    *
Gaac_spg1 CAGTCGGCTCAACACACTTTTAAGAGCAAAATTACAGTAAGACAAATGCATGCAGGCAAAATCAATGTCTC------------------------------------------------------------------------------ 2355
Gaac_spg2 CCAAAGTGTGGGCCGCGAAATTACTGCAGGTGGGCCGCGAGAGGTCATTTACAACAAATAAATAAAGTTTT----------------------------------------------------------------------------- 2274
Gaac_spg3 CTGTCGGCTTAACACACTTTTAAGAGCAAAATCACAGTAAGACAAATGCATGCAGGCATAATCAATGACTT------------------------------------------------------------------------------ 2235
Gaac_spg4 CAGTCGGCTCAACACACTTTTAAGAGCAAAATCACAGTAAGATAAATGCATGCAGGCATAATCAATGTCTC------------------------------------------------------------------------------ 1854
Gaac_spg5 CAGTCGGCTCAACACACTTTTAAGAGCAAAATTACAGTAAGACAAATGCATGCAGGCAAAATCAATGTCTC------------------------------------------------------------------------------ 2472
Gaac_spg6 CAGTCGGCTCAACAAACTT--AAGAGCAAAATCACATTAAGACAAATGCATGCAGGCATAATCAATATCTCATGTTAATTAAAGGGCGGCACGGTGGTGTGGTGGTTAGCACCGTTGCCTCACAGCAAGAAGGTTTCAGGTTCGATTCTG 1838
Gaac_spg7 CAGTCGGCTCAACACAC--GTAAGAGCAAAATCACATTAAGACAAGTGCATCCAGGCATAATCAATGTCTC------------------------------------------------------------------------------ 2355
          ......3310......3320......3330......3340......3350......3360......3370......3380......3390......3400......3410......3420......3430......3440......3450

Gaac_spg1 ------------------------------------------------------------------------------------------------------------------------------------------------------ 2355
Gaac_spg2 ------------------------------------------------------------------------------------------------------------------------------------------------------ 2274
Gaac_spg3 ------------------------------------------------------------------------------------------------------------------------------------------------------ 2235
Gaac_spg4 ------------------------------------------------------------------------------------------------------------------------------------------------------ 1854
Gaac_spg5 ------------------------------------------------------------------------------------------------------------------------------------------------------ 2472
Gaac_spg6 CCCAGTGGCCTTTCTGTGTGGAGTCTGCATGTCTGTATGTCTCTGTGATTGGCTGGCGACCAATCCAAGGTGTACCCCGTCTCTCGCCCGAAGTTGGCTGGGATGGACTCCAGCCCCCCCGCGACCCTGTGTGTAGGATAAGCGGTTGA 1988
Gaac_spg7 ------------------------------------------------------------------------------------------------------------------------------------------------------ 2355
          ......3460......3470......3480......3490......3500......3510......3520......3530......3540......3550......3560......3570......3580......3590......3600
```

5

```
                                *  *                                                                                                             **        *
Gaac_spg1 --------------ATGTTAATTGGAACATACAACACTAACCTCACTCACTGAAGCCCATGTGTTTAAACTTGTTATTAA-GGTGACACTGGAGTCGGAGCTGTGCACCGACATGTGTGGACTGTGTGGAAAACAAACGTTGCAGGTAT 2490
Gaac_spg2 --------------TTAATTTTCAATTTTTAAAAGTTTGAAATGAATATAAAAATATTTATGATGCGGCACATTAGTATGTGAAACATAGCCCTCTTGTAGTATTAAAGTAGATATATGGCATTTTTCGCACTCCAAGGCGCACTGTAA 2410
Gaac_spg3 --------------AAGTTAATTGGAACACGTGG-------------------------------------------------------------------------------------------AAAACGTTGCAGGTAT 2271
Gaac_spg4 --------------AAGTTAGTTGGAACATGCAACACTAATTTCACT----GAAGCCCATTTGTTTAAACTTGTTGTTAAAGGTGACATTGGAGTCGGAACTGAACACCACATCTGGCTGTGTGGAAAACAAACATTGCAGGTAT 1986
Gaac_spg5 --------------ATGTTAATTGGAACATACAACACTAACCTCACTCACTGAAGCCCATGTGTTTAAACTTGTTATTAA-GGTGACACTGGAGTCGGAGCTGTGCACCGACATGTGTGGACTGTGTGGAAAACAAACGTTGCAGGTAT 2607
Gaac_spg6 CAATGGATGGATGGATGTTAATTAAAATATCAAAACTAACCTCACTCACTGAAGCCCATGTGTTTAAACTTGTTAGTAA-GGTGACACTGGAGTCGGAGCTGTGCACCGACATGTGTGGACTGTGTGGAAAACAAACGTTGGAAGTAT 2137
Gaac_spg7 --------------AAGTTAATTGGAACATACAACACTAAACTCACTCAGTGAAGCCCATGTGTTTAAACTTGTTATTAA-GGTGACACTGGAGTCGGAGCTGAGCACCGACATGTGTGGACTGTGTGGAAAACAAACCTTGCAGGTAT 2487
          ......3610......3620......3630......3640......3650......3660......3670......3680......3690......3700......3710......3720......3730......3740......3750

             ** *  *   **   **          *    ** *  *   *             *      *      *      *            *      ** *    *        *     *  * *       **                   *     *
Gaac_spg1 TA---TTAACTGACTGGAAGTGAGACATTGATTGTTGTAATATAG-----TAGTAACATTGACAGCAGTATTTTTAGTAGCAACTGCAATCATTTCTTTTGGTGGTAGTGAAAGTGGTACAAATAGTGATTTA----AATAGCCAAAACA 2628
Gaac_spg2 AGGCTTTAATTTTCTCAAAAACGACAGTGCCTTATAATCCTTATATATATGGATCAATTGGTAATCGGTTGATCCATACTGGTTGTACACGGCGCTCAAGGCGCTCTGCCAAACATGCCAAAGCACGGCCTTCAACGGCGAGATGCTGA 2560
Gaac_spg3 TA---TTCACTGACTGGAAGTGAGACATTGATTGTTGTAATATAG---AGTCGTAGCATTGACAGCAGTACTTCCAGTAGCAGCTGCAATCATTTCTTTTGGTGGTGGTGAAA-----------GTGATTTG----AATAGTCAAA-CC 2398
Gaac_spg4 TA---TTAACTGACTGGAAGTGAGACATTGATTGTTGTAATATAG---AGTAGTAACATTGAAAGCAGTCATTTCAGGAGCAGCTGCAATCATTTCTTTTGTTTGGTGGTGAAAGTGGTAATAAATAGTGATTTAGCCAAATAGCCAAA-CA 2129
Gaac_spg5 TA---TTAACTGACTGGAAGTGAGACATTGATTGTTGTAATATAG-----TAGTAACATTGACAGCAGTATTTTTAGTAGCAACTGCAATCATTTCTTTTGGTGGTAGTGAAAGTGGTACAAATAGTGATTTA----AATAGCCAAAACA 2745
Gaac_spg6 TA---TTAACTGACTGGAAGTGAGACATTGATTGTTGTAATATAGC--AGTAGTAACATTGACAGCAGTATTTTTAGTAGCAACTGCAATCATTTCTTTTGGTGGTGGTGAAAGTGATACAAATAGTGATTTA----AATAGCCAAAACA 2278
Gaac_spg7 TAGAGTTAAATGACTGGAACTTTGACATTGATTGTTGTAATATAGCATATAAGTAACATTCACAGCAGTA-TTTTAGTAGCAACTGTAATCATTTATTTTAGTGGTGGTGAAAGTGGTACAAATAGTGATTTA----AATAGCCAAAACA 2632
          ......3760......3770......3780......3790......3800......3810......3820......3830......3840......3850......3860......3870......3880......3890......3900
```

```
                                                6
           *          ** **   **   *   *   **          *  **       *   *** *         **                        *            *               *        *    *  *
Gaac_spg1 ATTGTTTGCATCCAGTTTGGTTTAACAAAGTACATTTTTCTTGACATCAGGCCACAGGGACGAGTTGATCAGAGAAAGCAAGCTTCACGACCACAGATGTAAAATCAGAGATCCTGTGTTGCAAAAAAATCACGTGAGTTAACAGATCTA  2778
Gaac_spg2 GCGGGCGCTCAAGCGTGTGAGATATGGAGCTTGTTTCAGGGCGCGTCGGAGAAACAAAGCTGATGAGATAATGAGATTAAAGAGCGAGAGAAGGCGTCCTTTTCTTTACAGTAGCTGAACCATCTCAACGGGGAAAAAAATTATTCTAAA  2710
Gaac_spg3 TTTGTTTGCATCCAGTTTGGTTTAACAATGTATATTTTTCATGACATCAGGCCACAGGGACAAGTTGATCAGAGACAGCAAGCTCCATGACCACAAATGTAAAACCAGCAGTCCTGTATTGCAAAAAAACGATGTAAGTTAACAGTTGTA  2548
Gaac_spg4 TTTGTTTGCGTCCAGTTTGGTTTAACAATGTACATTTTTCATGACATCAGGCCACAGGGACAAGTTGATCAGAGACAGCAAGCTTCATGACCACAAATGTAAAACCAGCAGTCCTGTGTTGCAAAAAAATGATGTAAGTTAACAGTTGTA  2279
Gaac_spg5 ATTGTTTGCATCCAGTTTGGTTTAACAAAGTACATTTTTCTTGACATTAGGCCACAGGGACGAGTTGATCAGAGAAAGCAAGCTTCACGACCACAGATGTAAAATCAGAGATCCTGTGTTGCAAAAAAATCACGTGAGTTAACAGATCTA  2895
Gaac_spg6 ATTGTTTGCATCCAGTTTGGTTTAACAAAGTACATTTCTCTTGACATCAGGCCACAGGGACGAGTTGATCAGAGACAGCAAGCTTCTTGACCACAGATGTAAAATCAGAGATCCTGTGTTGCAAAAAAATCATGTGAGTTAACAGTTCTA  2428
Gaac_spg7 ATTGTTTGTATCCAGTTTGGTTTAACAATGTACATTTT--------TCAGGCCACCGGCACGAGTTGATCAGAGACAGCCAGCTTCATGACCTCAGATGTAAAACGAGAGATCCTGTGCTGCAAAAGAATCATGTGAGTTAACAGTTGTA  2774
          ......3910......3920......3930......3940......3950......3960......3970......3980......3990......4000......4010......4020......4030......4040......4050

          *  *   *   ****
Gaac_spg1 ATGATTAACTCATTTCGA------------------------------------------------------------------------------------------------------------------------------------  2796
Gaac_spg2 AGGAGGCAAAGATTTAAGGCGTGGATGGCGAGGGGGCGGCGGGAGCGGACGCACCTCGCAGCATCGATCGGACCCTGAAAGCACCGTCTCTACCATACTGTCAAAAAGAACAACAGCGAGCGCGTAGAAAACTCCTTCGAGCGCGAGCAG  2860
Gaac_spg3 ATGATTAACTTATTTCAA--------------------------------------------------------------------------------------------ACACAGCCAGGTTTACGCTGTGATATTAGATTGCAGTA  2604
Gaac_spg4 ATGATTAACTCATTTCAA--------------------------------------------------------------------------------------------ACACAGCCAAGTTCACGCTGTGATATTAGATTCCAGTA  2335
Gaac_spg5 ATGATTAACTCATTTCGA------------------------------------------------------------------------------------------------------------------------------------  2913
Gaac_spg6 ATGATTAACTCATTTCGA------------------------------------------------------------------------------------------------------------------------------------  2446
Gaac_spg7 ATAATTAACTAATTTCAAACACAGCTTACGCTGTGTTACTAGATTCCAGTATGCTATTGGTAAGGCTCTTTTCTACCTACTTTCTAAAAGAAAAACCTCAATGTATTAACAATAATAATCATAACAATGAACCAATAGCAATTAAGTAGT  2924
          ......4060......4070......4080......4090......4100......4110......4120......4130......4140......4150......4160......4170......4180......4190......4200

                                                                                                                                           ****** * * *    ** *
Gaac_spg1 ---------------------------------------------------------------------------------------------------------------------------CAAGCAAAATAGAACACAA--TAGAATACA  2824
Gaac_spg2 AGATTCTCCTCGCGAGCATTCATGGATCTCACTGGAAAGAGTTTGAAAGAATTCACGGTACCAACTTTTATTAATTAGTTAATTAGTGACTCATGGGAAAAAGAGGATGCTAAAGTAAAATCAGTAAAATAGAACACAA--TAGATACAT  3008
Gaac_spg3 TGCTATTGGTAAGGCTCTTTTCTACCTACTTTCATCTTTTTTTTCACTCATTGATCTCACGGTACTGACTTTTATTAATTAATTGGGGACTCATAGGAAAAAGAGGACGCTTAAGTAAAATAAGAAAAATAAAACACAA--TAGAATACA  2752
Gaac_spg4 TGCTATTGGTAACGCTCTTTTCTACCTACTAATATCTTTTTTTT------------------TTCACTCATTTATTAATTCATTAAGGACTCATAGGAAAAAGAGGACGCTTAATTAAAATAAGAAAAATAGAACACAA--TAGAATACA  2465
Gaac_spg5 ---------------------------------------------------------------------------------------------------------------------------CAAGCAAAATAGAACACAA--TAGAATACA  2941
Gaac_spg6 ---------------------------------------------------------------------------------------------------------------------------CAAGCAAAATATAACACAA--TAGAATATA  2474
Gaac_spg7 CATTTATTGATTTTTATTGAATAATCATACATTTTATTTGTATAGTACATTTCACTCAAGAAATTACGCATGTGATTTATAATACAATTAAATATACCTCCATAGTTAATAGAATTTTGGGT---AAAATAGA-CGCGTGTTAAAATACC  3070
          ......4210......4220......4230......4240......4250......4260......4270......4280......4290......4300......4310......4320......4330......4340......4350

           *  *    *                                                                                                                     *   *   *   *   *
Gaac_spg1 -TGGACA-TATTAGGA--------------------------------------------------------------------------------------------------------AGCATCTCCATTCTC----------------------  2853
Gaac_spg2 -TGAATA-CATAGACATAGCAGGTGGAAAATAAAACGC--AAATATAAGAAATAATATAAGATCAGTAACCATATTTTCAGTACTCACTTTCAACACAAACACAGCAGCCGCAGCATCTCCATTCTC----------------------  3131
Gaac_spg3 -TAGACA-TA----GCA----GGTGGAAAATAAACCCATGAAAAAATAAGAAATAATATTAAGATCAGTAATGATTTCACTACATAACTACAAACATAAACACAGCAGCCGGATAATCTGCATTTTC----------------------  2869
Gaac_spg4 -TAGACA-TA----GCA----GGTGGAAAATAAACCCATGAAAAAATAAGAAATAATATTAAGATCAGTAATGATTTCACTACATAACTACAAACATAAACACAGCAGCCGGATAATCTGCATTTTC----------------------  2582
Gaac_spg5 -TGGACA-TATTAGGA--------------------------------------------------------------------------------------------------------AGCATCTCCATTCTC----------------------  2970
Gaac_spg6 -TAGACA-TATTAGGC--------------------------------------------------------------------------------------------------------AGCATCTCCATTCTC----------------------  2503
Gaac_spg7 ATGAAATTGAGTAAAAAAA--GACGAAGAATATAAAAGACTACTGACTTACTGATGATTGACAGAGGCATAGCATAAGATTAAAAAATAAAACCCAAGGAATTATGAGTAGTATCTTGATCCGTAACCACATATTCAGAATTCACTTGCA  3218
          ......4360......4370......4380......4390......4400......4410......4420......4430......4440......4450......4460......4470......4480......4490......4500

                                                  ** * ******** *********  ***         ***               * **    *                 *     *   *   *       *   *
Gaac_spg1 ------------------------------------------TTCTCTTCTTTTCTACATCATTGGGGGAGGGTTGATTTTATGAGACTGAGAGACTCACAAAATAAGTGAA---CA-ATTATGTGTCCTAGTTCCAGATATTAAAGTGG  2957
Gaac_spg2 ------------------------------------------TTCTGTTCTTTTCCACATCATTGGGGGA-------TTTTAGAAGAC--AGAGACTCACA-AAATTGAGCAATTCATATTATGTGTCCTTGTTCCAGACATTAAAGTGG  3229
Gaac_spg3 ------------------------------------------TTCTGTTCTTTTCTACATCATTGGTGGAAGGTTTATTTTTTAAGATTGAGACTCACAAAATAAGTGAACAATCCATAGTATGTGTCCTAGTTCCAGATATTCAAGTGG  2977
Gaac_spg4 ------------------------------------------TTCTGTTCTTTTCTACATCATTGGTGGAAGATTTATTTTTTAAGATTGAGACTCACAAATTAATTGAACAATCCATAGTATGTGTCCTAGTTCCAGATTTTCAAGTAA  2690
Gaac_spg5 ------------------------------------------TTCTCTTCTTTTCTACATCATTGGGGGAGGGTTGATTTTATGAGACTGAGAGACTCACAAAATAAGTGAA---CA-ATTATGTGTCCTAGTTCCAGATATTAAAGTGG  3074
Gaac_spg6 ------------------------------------------TTCTCTTCTTTTCTACATCATTGCGGGAGGGTTGATTTTATAAGACAGAGAGACTCACAAAATAAGTGAA---CC-ATTATGTGTCCTAGTTCCAGATATTAAAGTGG  2607
Gaac_spg7 TCAATAAACACAGTAGCAGCATCTCTATTCACTTCTACTTTGTTTTGTTCTTTTCTACATCATTGGTGGAAGGTTGAATTTTCTCACAAAGTGCACTCAATCCATACTGTATGTATTAGTTTCAAATGTTCAATTGGTTTCTCTGATGAT  3368
          ......4510......4520......4530......4540......4550......4560......4570......4580......4590......4600......4610......4620......4630......4640......4650

                               7
           **                  * *   **             *** ****  **** *** * ****** ******** *** * *******  *** ******* ******* ****** ***      **************** **
Gaac_spg1 TTTCCCTGATTATATCTACAGATATGCCGTCGATTCTTTTTGAAAACCAAGAACTGTCTGCAAGACAACAATTCTCACTATCACCGACTCTGTAAAGAGAACATTTGTGGCTTTGAAAACAGCCAGAGCATCTTCTGTCCTTTCTTCCAA  3107
Gaac_spg2 TTTCCCTGATTATATCTGCAGATATGCCGTCGATTCTTTCGGAAAACCAAGAACTGTCTGCAAGACAACAATTCTCACTATCACCGACTCTGTAAAGAGAACATTTGTGGCTTTTAAAACAGCCAGAACGTCTTCTGTCCTTTCTTCCAA  3379
Gaac_spg3 TTTCCTCATGTT----TGCAGATATGCCGTCAATTCTTTCAAGAAATCAAGGACTGTGTGCGATACAACAATTCTCACTATCAGCAACTCTGTGAAGAGAACATTTTTGGCTTTGAAAACAGCCAGAGCGTCTTCTGTCCTTTCTTCAAA  3123
Gaac_spg4 TTTCCTCGTGTT----TGCAGATATGCCGTCAATTCTTTCAGGAAATCAAGGACTGTGTGCAATACAACATTTCTCACTATCAGCGACTCTGTGAAGAGAACATTTTTGGCTTTGAAAACAACCAGAGCGTCTTCTGTCCTTTCTTCAAA  2836
Gaac_spg5 TTTCCTGATTATATCTACAGATATGCCGTCGATTCTTTTTGAAAACCAAGAACTGTCTGCAAGACAACAATTCTCACTATCACCGACTCTGTAAAGAGAACATTTGTGGCTTTGAAAACAGCCAGAGCATCTTCTGTCCTTTCTTCCAA  3224
Gaac_spg6 TTTCCCTGATTATATCTACAGATATGTCGTCAATTATTTCGGAAAACCAAGAACTGTCTGCAAGACAACAATTCTCACTATCACCGACTCTGTAAAGAGAACATTTGTGGCTTTGAAAACAGCCAGAGCATCTTCTGTCCTTTCTTCCAA  2757
Gaac_spg7 ATTTGTAGGTATGCAGCCAATTCTTTC-GT------------AAAACCAAGAGCTGTCTGCAAGACAACAATTCTCACTTTCACCAACTCTGTCGAGACAACATTTTTGGCTTTGAAAACAGCCATGAGGTCTTCTGTCCTTTCTTCCAA  3505
          ......4660......4670......4680......4690......4700......4710......4720......4730......4740......4750......4760......4770......4780......4790......4800

          *** **************** *** ************  ****  *    * *   *    *  *
Gaac_spg1 GAAGTTGCAAGCCAGTGTAACCAATCAAGAATCAACCGATTTTGGAGACGTTTAACCAGATGTGGTAGGCACTCTT------------------------------------------------------------------------------  3183
Gaac_spg2 GAAGTTGCAAGCCAGTGTAATCAATCAAGAATCAACCAATTTTTGAAACTTCTCCTCTGTAGCAGCTCCGTTGACCTCGTTAACCAGTCCAGCAAAACCCTTTGTATGGTCTAATGAGTGCAAACAATCTTTTGAGAGCCTAAAAGGAAT  3529
Gaac_spg3 GAATTTGCAAGCCAGTGTAACCAATCAAGAATCAACCGATTTTGGAGGGATTTAACCAAATGTGGTAAGCACTCTT------------------------------------------------------------------------------  3199
Gaac_spg4 GAATTTGCAAGCCAGTGTAACCAAACAAGAATCAACCGATTTTGGAGACGTTTAACCAAATGTGGTAAGCACTCTT------------------------------------------------------------------------------  2912
Gaac_spg5 GAAGTTGCAAGCCAGTGTAACCAATCAAGAATCAACCGATTTTGGAGACGTTTAACCAGATGTGGTAGGCACTCTT------------------------------------------------------------------------------  3300
Gaac_spg6 GAAATTGCAAGCCAGTGTAACCAATCAAGAATCAACCGTTTTTGTAGACGTTTAACCAGATGTGGTAGGCACTCTT------------------------------------------------------------------------------  2833
Gaac_spg7 GAAGTTGCAAGCCAGTGTAACCAATCAAGAATCAACCGATTTTGGAGACGTTTAACCAGATGTGGTAAGCACTCTTAAAACTATACTGAAAAAAATATATATATGTTAAAAACATTTGCACGATTGCTGATTT----------------  3638
          ......4810......4820......4830......4840......4850......4860......4870......4880......4890......4900......4910......4920......4930......4940......4950

Gaac_spg1 ------------------------------------------------------------------------------------------------------------------------------------------------------  3183
Gaac_spg2 AATGTGTTGCACTCCAGTGCTGAAGGCCCCGGATTTCATGTTGCCTTTCAAGTTGGAGGTTGATGCCAGCGCAGTTGGAGCTGGTGCAGTTCTTCTGCAGGAGGATGCCCACGGCATCGACCACCCTGTGAGTTATTTCTCATGGAAATT  3679
Gaac_spg3 ------------------------------------------------------------------------------------------------------------------------------------------------------  3199
Gaac_spg4 ------------------------------------------------------------------------------------------------------------------------------------------------------  2912
Gaac_spg5 ------------------------------------------------------------------------------------------------------------------------------------------------------  3300
Gaac_spg6 ------------------------------------------------------------------------------------------------------------------------------------------------------  2833
Gaac_spg7 ------------------------------------------------------------------------------------------------------------------------------------------------------  3638
          ......4960......4970......4980......4990......5000......5010......5020......5030......5040......5050......5060......5070......5080......5090......5100

                                                                                                                                     8
                                                                                   **               *                 ** *  * *       *        * * *     *
Gaac_spg1 ------CAAAAA--CAAAATGAAAAAAAA--------------------TCTTATGG---ACTCATTGGAGAAGAACACATGATGTTAT-------TT-------TCTGTGGTTTAATGCAGCGAAGCCGAGGTGTCCAGGAGACCTGAT  3288
Gaac_spg2 CAACAGGCATCAGCTTGGGTATTCAACAATAGAGAAGGAGGCTCTGGCCCTGTTGTTTGCTCTACAGCATTTTGAGGTTTATCTTGGGTCAAGTGAAA-------AGCCTGTTCAAGTATTCACCGATCATAACCCTCTTGTGTTTCTTT  3822
Gaac_spg3 ------CAAAAAAACAAAAAAACAAAAAA------------ATCATTGATATTATAGGACACTCATTGGAGAAGAAAACATTAAGTTATTGCCCATTT-------TCTGTGGTTTAATGCAGCAGAACCGAGGTGTCCAGGAGACCTGAT  3324
Gaac_spg4 ------CAAAAA--CAAACAAACAACAAAACAACAAAAAAAAAGATTGG-TATTATAGGACACTCATTGGAGAAGAACACATTATGTTATTGCCCATTT-------TCTGTGCTTTAATGCAGCAGAACCGAGGTGTCCAGGAGACCTGAT  3046
Gaac_spg5 ------CAAAAA--CAAAATGAAAAAAAA--------------------TCTTATGG---ACTCATTGGAGAAGAACACATGATGTTAT-------TT-------TCTGTGGTTTAATGCAGCGAAGCCGAGGTGTCCAGGAGACCTGAT  3405
Gaac_spg6 ------CAAAAA--CAAAATTTAAAAAAA--------------------TCTTGG------CTCATTGGAGCAGAACACATGATGTTAT-------TT-------TTTGTGGTTTAATGCAGCAAAGCCGAGGTGTCCAGGAGACCTGAT  2935
Gaac_spg7 ----------------------------------------------------------------------GAAGAACACATGATGTTAT-------TTCCTAGTGTCTGTGGTTTAATGCAGAGAAACCAAGTTGTCCAGGAGACCTGAT  3711
          ......5110......5120......5130......5140......5150......5160......5170......5180......5190......5200......5210......5220......5230......5240......5250

           *      * *    *   **            *     *     *      *   **      *    *   *  *    *  ***    *     **     *      *     *            *     **     * *      *     *
Gaac_spg1 TTATGAGAAAAAAAGGTCCAGCATTTATTCCCAGCTGCTCCAACCCGAACCCTGCACCCTTCTACCAGGAACTCACTGAAACCTGTGCCTGTCCAAACGGTGAAAGAGCACGATCTGTCTTTATGATGTAGCTCTTTAAAAGTGTCATAGC  3438
Gaac_spg2 CCAGGATGCATAACTACAACCAACGTCCGATGCGTTGGTCTCTCATTGTTCAGGACTACAATCTTAACATCGTGCATAAAAAGGGATCTGAAAATGTTCTTGCTGATGCTTTGTCTCGTGCGGTATAGTTATGTTCCCCTATTAATTTAG  3972
Gaac_spg3 TTATAGGGAAAAGGGTCCAGCAGTTATTCCCAGCTGCTCCAACCCGAAACCTCCACCCTTCTACCAGGAACTCACTGAAAGCTGTGCCTGTCCAGAGGGTGAAAGAGCACGATATGTCTTTATGATGTAGCTCTCTAAAAGTTTCATAGC  3474
Gaac_spg4 TTATAGGGAAAAGGGTCCAGCAGTTATTCCCAGCTGCTCCAACCCGAAACCTCTACCCTTCTACCAGGAACTCACTGAAAGCTGTGCCTGTCCAGAGGGTGAAAGAGCACAATACGTCTTTATGATGTAGCTCTCTAAAAGTTTCATAGC  3196
Gaac_spg5 TTATGAGAAAAAAAGGTCCAGCATTTATTCCCAGCTGCTCCAACCCGAACCCTGCACCCTTCTACCAGGAACTCACTGAAACCTGTGCCTGTCCAAAGGGTGAAAGAGCACGATCTGTCTTTATGATGTAGCTCTTTAAAAGTTTCATAGC  3555
Gaac_spg6 TTATGAGAAAAAAAGGTCCAGCATTTATTCCCAGCTGCTCCAACCCGAACCCTGCACCCTTCTACCAGGAACTCACTGAAACCTGTGCCTGTCCAAAGGGTGAAAGAGCACAATCTGTCTTTATGATGTAACTCTTTAAAAGTTTCATAGC  3085
Gaac_spg7 TTATGAGAAAAAGGGTCCCGCATTTATTCCCAGCTGCTCCAACCCAGACCCTGCACCCTTCTACCAGGAACTTACTGAAACCTGTGTATGTCCAAGAGGTGAAAG----GAATTTGTCTTTGTGATGTAGCTCTTCACATGTTTGATAGT  3857
          ......5260......5270......5280......5290......5300......5310......5320......5330......5340......5350......5360......5370......5380......5390......5400

                                                         9
           *  * *   *     **  *  *    *           * ****    * *                          * *      *       * **  *     *           *                * *
Gaac_spg1 TTGTTTAGCCTCAATGAGCCTAATAACATGGTGT---TATTTGTATTTTAGGTAAAGTTTTGAATAATTGTGAAAAGGGCTATCGCTGTATACCTAAATCCAGCTGCTCCTGTGAGTTCGCTGGCAAGACCTACGGAAACGGAGAAATAC  3585
Gaac_spg2 ATCGTAACTTTATGGGAGATTGTTTTTGTGGCAT---TCATTTTTTTTTCTGAGCTTATATGGGTGGGGGTGTTACATGCCAGACGGGGACGGTCTGCATGTGTTCTTGTCGTTCTCCCTCCTGCTCATTGGTTTCTCTCCCCCAGTGA  4119
Gaac_spg3 TTGTTTAGTCTCAATGAGCCTAATGACATGGTGT---TATTTGTATTTTAGGTAACGTTTTGAATAATGGTGCAAAGGGCTATCGCTGTATTCCTTGGTCCAACTGCTCTTGTGAGTTTGCTGGCAAGAGTTACAGAAACGGAGAAATAC  3621
Gaac_spg4 TTGTTTAGCCTCAATGAGCCTAATAACAT---GT---TATTTGTATTTTAGGTAAAGTTTTGAATAATGGTGCAAAGGGCTATCGCTGTATTCCTTGGTCCAACTGCTCTTGTGAGTTTGCTGGCAAGAGTTACAGAAACGGAGAAATAC  3340
Gaac_spg5 TTGTTTAGCCTCAATAAGCCTAATAACATGGTGT---TATTTGTATTTTAGGTAAAGTTTTGAATAATTGTGAAAAGGGCTATCGCTGTATACGTAAATCCAGCTGCTCCTGTGAGTTCGCTGGCAAGACCTACGGAAACGGAGAAATAC  3702
Gaac_spg6 TTGTTTAGCCTCAATGAGCCTAATAACATGGTGC---TATTGGTATTTTAAGGAAGTTTTGAATAATTGTGCAAAGGGCTATCGCTGTATTCCTAAATCCAACTGCTCTTGTGAGTTTGCTGGCAAGACCTACAGAAACGGAGAAATAC  3232
Gaac_spg7 TTGTTTACCCTCCATGAGCCTGATAACATCATGTACAAATCTGTATTTTAGATAAAGTCTTCAATAATCATGCAATTGGTCATCACTGCATTCCTGAGTCCAGCTGCTCTTGTGAGTTTGCTGGCAAGAGCTACGGAAACGGAGAAATAC  4007
          ......5410......5420......5430......5440......5450......5460......5470......5480......5490......5500......5510......5520......5530......5540......5550

                  *   * **              * *      ** *               *     *        *       *     * *       *                  *           *
Gaac_spg1 GGAGTTCCAGGTGTCAGTCATGGTAATATACTTCAAACTTC--ATTTCAGACTCACAAGATGGTTCCCAGCAAAAAA-ATGGGCATACAAACTGTTTCCTTACCTCTTTATAAGTT-AATAACATGAACTATTCT------------TAA  3719
Gaac_spg2 TTACGCTGATTGGCCAACTCCAAACACAGGTGGAAAGCAATCAGCAGTTCCGGTGGCAGTCCATAAAGGAAGACTCCTGAGAAGTGAAAAGGGTGTGTGTTCTGTGCTGCGGGCTGGTGGCCAGTCTTCCTGTAC------------GTG  4257
Gaac_spg3 GACGTTCCAAGTGTCACTCATGGTAATATACTTCAAACTTTCAATTTCAGAGACACAAGATGGTCCCTAGCAAAATA--TGGGCAGATAAACAGTTGCCTTACCGCTTTTTAAGTTCAATAACATGATCTATTGT-------------AA  3756
Gaac_spg4 GACGTTCCAAGTGTCACTCATGGTAATATACTTCAAACTTT--ATTTTAAACACACACGATGATCCCCAGCAAAAAAAATGGGCATATATACTGCTGCTTTACCTCTGTTTATGTTCAATAACATGAACTATTC-------------AAA  3475
Gaac_spg5 GGAGTTCCAGGTGTCAGTCATGGTAATATACTTCAAACTTC--ATTTCAGACTCACAAGATGGTTCTTAGCAAAAAA-AGGGGCATATAAACTGTTTTCTTACCTCTTTACAAGTT-AATAACATGAACTATTCT------------TAA  3836
Gaac_spg6 GGAGTTCCAGGTGTCAGTCATGATAATATACTTTAAACTTT--ATTTCAGACTCACAAGATGGTGCCCAGCAAAAAA-ATGGGCATATAATCTGTTTCCTTACTTCTTT-TAAGTTCAATAACATGAACTATTCT------------TAA  3366
Gaac_spg7 GGAGTTCCAAGTGTCAGTCATGGTAATACACTTCAAACTTT--ATTTCAGACTCACAAGATGATCCCCAGCAAAAAA-ATGGGTGTATAAACTGTTTTCACACCTCATTTAAGTTAAATAAACTTAATAAAATGAAGTCATTTATGTAA  4154
          ......5560......5570......5580......5590......5600......5610......5620......5630......5640......5650......5660......5670......5680......5690......5700

                                      10
             **** *                 **          *        **    *     *        *  **     *  * **     *          *****    ***  * * *   *         * *    *     ** **
Gaac_spg1 GTTTTTAATT-CGGTTTTATCTTCACACAGTACGTGTGATGGTGGGAAATGGCGATGCTCAGAGAACTTTTGCCACAGAAGATGTGTCATTGAAGGCCAGTTTGTGACAACATTCGATGGAAAACAATATGTCCTCCCTAACAAATGTTT  3868
Gaac_spg2 AGCTTTAGCTGGCTGCTAGCAAAACTGGAGTTTTGTAAGGTGGAGGTTGAGGACCTTAGTAAGTTGGGGTACCCTGTACATTTTTGCACGTAGGTATACCTTTGTATAGACACACCAGGTGTAGTGGTTGTTGCCTTCCGTGTAACGTTT  4407
Gaac_spg3 GTTTTTAATTTAGGTTCTATTTTCACACAGTACGTGTCATGGTGTGGTATGGCGATGCTCAGAGAACTTTTGCCGCAGAAGATGTGTCATTGAAGGCCCGTTTGTGACAACATTCGATGGAAAACAACATGTCCTCCCTCAAAAATGTTC  3906
Gaac_spg4 GTTTTTAATTTAGGTTTTATTTTCACACAGTACGTGTCATGGTGTGGTATGGCGATGCTCAGAGAATTTTTGCCACAGAAGATGTGTCATTGAAGTCCCGTTTGTTACAACATTCGATGGAAAAAAATATGTCCTTCCTTACAAATGTGC  3625
Gaac_spg5 GTTTTTAAATTCGGTTTTATCTTCACACAGTACGTGTGATGGTGGGAAATGGCGATGCTCAGAGAACTTTTGCCACAGAAGATGTGTCATTGAAGGCCAGTTTGTGACAACATTCGATGGAAAACAATATGTCCTCCCTAACAAATGTTT  3986
Gaac_spg6 GTTTTTAATTTAGGTTTTATCTTCACACAGTACGTGTCATGGTGGGAAATGGAGATGCTCAGAGAACTTTTGCCACAGAAGATGTGTCATTGAAGGCCAGTTTGTGACAACATTCGATGGCAAACAATATGTCCTCCCCTACAAATGTTT  3516
Gaac_spg7 GTTTTTAATTTAGATTTTATCTTCAAACAGCACGTGCCATGGTGGTAAATGGCGATGCTCAGAGAACCTTTGCCACAGAAAATGTGTCATTGAAGGCCAGTTTGTGACAACATTCGATGGAAAACAATATGTCCTCCCTCGCAAATGTTT  4304
          ......5710......5720......5730......5740......5750......5760......5770......5780......5790......5800......5810......5820......5830......5840......5850
```

```
10
         * * *        **
Gaac_spg1 ATATGTGGCTTCAAAGGTAATTCTTTTCAATTCCCTAACATGTAACAT--------------TACAG---------ATGTATTCTATTTATGATAC------------------------------------------------------ 3941
Gaac_spg2 TCCTTTAGTCCTTTTTTTAGAGAAGCAGGTAAGGTATTTTGTTTGACTTGTTTTGG------ATGAAGAGCCAGTGGTGAGCTAGGCCTCGTTTTG------------------------------------------------------ 4497
Gaac_spg3 ATATGTGGCTTCAAAGGTAATCTTTGCCAATTCCCCATCATGTAACAGACACAGAA------TACATTCTTATCTTATATATTCTTTTCATTATAC------------------------------------------------------ 3996
Gaac_spg4 ATATTTAGCTTCAAAGGTAATTTTTGCCAATTCCCCAACATGTAAGAGACACAGAA------TATAG---------ATGTATTCTTTTCATGATAT------------------------------------------------------ 3706
Gaac_spg5 ATATGTGGCTTCAAAGGTAATTCTTTCCAATTTCCTAACATGTAACAT--------------TACAG---------ATGTATTCTATTTATGATAC------------------------------------------------------ 4059
Gaac_spg6 ATATGTGGCTTCAAAGGTAATTCTTTCCAATTTCCCAACGTCACGATGTAACAGACACAGAATACAG---------ATGTATA----TTATGGTAC------------------------------------------------------ 3599
Gaac_spg7 ATATGTCGCTTCAAAGGTAATTTTTCCCAATTCGCCAACATGTAATATTATCATTG------TATTACCTATTTCTTACTAATGTCGTGTTCACACCAAACCGGACGCAATTTATTACAAAGTTTATGCACAGACACAATGAGACTGGAA 4448
          ......5860......5870......5880......5890......5900......5910......5920......5930......5940......5950......5960......5970......5980......5990......6000

Gaac_spg1 ------------------------------------------------------------------------------------------------------------------------------------------------------ 3941
Gaac_spg2 ------------------------------------------------------------------------------------------------------------------------------------------------------ 4497
Gaac_spg3 ------------------------------------------------------------------------------------------------------------------------------------------------------ 3996
Gaac_spg4 ------------------------------------------------------------------------------------------------------------------------------------------------------ 3706
Gaac_spg5 ------------------------------------------------------------------------------------------------------------------------------------------------------ 4059
Gaac_spg6 ------------------------------------------------------------------------------------------------------------------------------------------------------ 3599
Gaac_spg7 CAAAGCAAAATTGCAATAGCGGCGCATTTGTTGCGTCGACCTTGCAGCAAACTCAATTCTGGCGAAGTTGACTGAATTTGCGCCAAACTGCAAAACGCACCAAATGTGAATGTTTAAAGATTTTAACTTTGGCGACGAAATGGCGTGACA 4598
          ......6010......6020......6030......6040......6050......6060......6070......6080......6090......6100......6110......6120......6130......6140......6150

Gaac_spg1 ------------------------------------------------------------------------------------------------------------------------------------------------------ 3941
Gaac_spg2 ------------------------------------------------------------------------------------------------------------------------------------------------------ 4497
Gaac_spg3 ------------------------------------------------------------------------------------------------------------------------------------------------------ 3996
Gaac_spg4 ------------------------------------------------------------------------------------------------------------------------------------------------------ 3706
Gaac_spg5 ------------------------------------------------------------------------------------------------------------------------------------------------------ 4059
Gaac_spg6 ------------------------------------------------------------------------------------------------------------------------------------------------------ 3599
Gaac_spg7 TTTTCCCCCTGTTGTCACTGAAGTCCTGCAGTTTGTAAATCAGACAAAAATAATAGTAACGTTAACTGTGTGTGGGTACCACGAGCTCTACGACACATCCTCGTACCTATATTGCTAGCGTTCTAGGGAAGACTTCACTACACCACACAT 4748
          ......6160......6170......6180......6190......6200......6210......6220......6230......6240......6250......6260......6270......6280......6290......6300

Gaac_spg1 ------------------------------------------------------------------------------------------------------------------------------------------------------ 3941
Gaac_spg2 ------------------------------------------------------------------------------------------------------------------------------------------------------ 4497
Gaac_spg3 ------------------------------------------------------------------------------------------------------------------------------------------------------ 3996
Gaac_spg4 ------------------------------------------------------------------------------------------------------------------------------------------------------ 3706
Gaac_spg5 ------------------------------------------------------------------------------------------------------------------------------------------------------ 4059
Gaac_spg6 ------------------------------------------------------------------------------------------------------------------------------------------------------ 3599
Gaac_spg7 GCATTTTTTGAAACATCCTATCATTGCACTGAATAGTAAACTACGGCATATTAAATTAGTGGATAATTATATTGTTAGGACGGACTCCTACTAAATATTAAGTAATATTGTAGGGCTATCTAGGGTTATCTAACTGGCTAACAATTAGCC 4898
          ......6310......6320......6330......6340......6350......6360......6370......6380......6390......6400......6410......6420......6430......6440......6450

Gaac_spg1 ------------------------------------------------------------------------------------------------------------------------------------------------------ 3941
Gaac_spg2 ------------------------------------------------------------------------------------------------------------------------------------------------------ 4497
Gaac_spg3 ------------------------------------------------------------------------------------------------------------------------------------------------------ 3996
Gaac_spg4 ------------------------------------------------------------------------------------------------------------------------------------------------------ 3706
Gaac_spg5 ------------------------------------------------------------------------------------------------------------------------------------------------------ 4059
Gaac_spg6 ------------------------------------------------------------------------------------------------------------------------------------------------------ 3599
Gaac_spg7 TTACGTACCACTGAATGCGGCAGTTATCCAAACGCATTTTTTAGAAGTTGGTGAAATTCACTAAGCTGTCTGCGCTTATGAATGCTATTTGTACCAAATTTGGTGGTCCGACATGAGTAAGACGGCAGACTCTTCCACAAAAGGTACAT 5048
          ......6460......6470......6480......6490......6500......6510......6520......6530......6540......6550......6560......6570......6580......6590......6600

Gaac_spg1 ------------------------------------------------------------------------------------------------------------------------------------------------------ 3941
Gaac_spg2 ------------------------------------------------------------------------------------------------------------------------------------------------------ 4497
Gaac_spg3 ------------------------------------------------------------------------------------------------------------------------------------------------------ 3996
Gaac_spg4 ------------------------------------------------------------------------------------------------------------------------------------------------------ 3706
Gaac_spg5 ------------------------------------------------------------------------------------------------------------------------------------------------------ 4059
Gaac_spg6 ------------------------------------------------------------------------------------------------------------------------------------------------------ 3599
Gaac_spg7 AGTCTATATGGCTTCCATGGTTGATGCGGAGGAAAAACAATGGCGGAAAGTTGAACTGTTTTCACGCTTCTTCCCGGGGCCACTCTCCAGCGTGTCGAGCGTGAAAGATGCAAGGCATGCAAATTAACCAGAAATGGTCCCAAGACCACT 5198
          ......6610......6620......6630......6640......6650......6660......6670......6680......6690......6700......6710......6720......6730......6740......6750

                                                                             11
                                                       * *     ***  **    ** *              *   *   *   *   *                    *   *   **    *
Gaac_spg1 -------------------------------------------------TAACTAGTCTTTTACTTTAAAGGGTCCCAACTGGATAATAATAATAGAGTTTTCACAAAAAAAACTCCACATTAGAAAGGTTACGGTTCAGCTCATGGAGGT 4043
Gaac_spg2 -------------------------------------------------TTATATTCATTTCATTTGTTTGCACAACCTGTAGGTCCCATCTTCCACCATCTCCCTCCCTCCTGTTGGGAGCCATCTTTTTGTGTAAGAATAAAAACTAG 4599
Gaac_spg3 -------------------------------------------------TAACCACCCTTTTACTTTAAAGGGTCCCAACTGGAGAATAAGAATACATTTTTCACGAAAAGGACTCTATCTTAGAAAGGTTGTGGTCCGGTTCTCTGAGGT 4098
Gaac_spg4 -------------------------------------------------TAACTACTCTTTTACTTTAAAGGGTCCCAGCTGGAGAATAAGAATACATTTTTCGCGAAAAGGACTCTATCTTAGAAAGGTTGTCGTGCGGCTCCCAGGGGT 3808
Gaac_spg5 -------------------------------------------------TAACTAGTCTTTTACTTTAAAGGGTCCCAACTGGATAATAATAATAGAGTTTTCGCAAAAAAAACTCCACATAAGAAAGGTTACGGTTCAGCTCATGGAGGT 4161
Gaac_spg6 -------------------------------------------------TAACTAGTCTTTTACTTTAAAGGGTCCCAACTGGATAATAATAATAGAGTTTTCGCAAAAAAAACTCCACATAAGAAAGGTTACGGTTCAGCTCATGGAGGT 3701
Gaac_spg7 CTGCGCAATTAGAGTCTCTTAGGCGATGCGTTTGGTGTGAATACCACATAACTACTGTTTTTCTTTAAAGGGCCCCAACTGGATGATAACAATACAGTTTAATCAAAAAGGACTCTCTCTTAGAAAGGTCATGGTTCATCTTTCAAAGGT 5348
          ......6760......6770......6780......6790......6800......6810......6820......6830......6840......6850......6860......6870......6880......6890......6900

                                                                                                                                      12
 * *  **   **                 *     *           *  *     ***  *  **         *          *
Gaac_spg1 ACAAGCAGTCTTTGAGGAATTAATTTAATATAAAACATTCTTTCAAACACATGTAATGCAACAATAGTGTGCTATAATGGAATGTAAACATCTTTT-TTTTGCAGGAACTGTTTGTATTCAAAAAGAACAAGGTTTTGTTTGATGGACAG 4192
Gaac_spg2 AAAGACACCTTTTACCCTGACTCTGTCGCCTGTCTTTGATTGTTGATTTACTGTCCTGGAATCTACAAAAGGGCCGTAACA--------------------------------------------------------------------- 4680
Gaac_spg3 ACAAGCAGTCTT-GAGGAATTAATTTAAAATAACACAATCTTTCAAACCCATGTAATTCAAGAGTACTGTGGTGTCATGGAATATAAACA--TTTT-TTTTGCAGGAACTGTTTGTATTCAAAGAGAACAAGGTTTTGTGGAATGGGCAG 4244
Gaac_spg4 ACAAGCAGTCTT-GAGGAAT-AATTTAAAATAACACAATCTTTCAAACACATGTATTGCAACAGTAGTGTGCTATCATGGAATAAAAACACCTTTT-TTTTGCAGGAACTGATTGTATTCAGAAAGAACAAGGTTTTGTGGAATTGGCAG 3955
Gaac_spg5 ACAAGCAGTCTTTGAGGAATTCATTTAAAATAACAATATCTTTCGAACACATGTAATGAAACAGTAGTGTGCTATCATC-AATATAAACACCTTTT-TTT-GCAGGAACTGTTTGTATTCACGAAGAACAAGGCCTTGTTTGATGGGCAG 4308
Gaac_spg6 ACAAGCAGTCTTTGAGGAATTCATTTAAAATAACAATATCTTTCGAACACATGTAATGAAACAGTAGTGTGCTATCATGGAATATAAACA-CCTTT-TTTTGCAGGAACTGTTTGTATTCACGAAGAACAAGGCCTTGTTCGATGGGCAG 3849
Gaac_spg7 ACAAGCAATCTTTGAATTTAAAACATAATCTG----------TCAAACAATTGTAATGCAACACTAGGGTGCCGTCATGGAATATAAACAACTTTTCTTTTGCAGGAACTGTTTGTATTCAAAAATAGCAAGGTTGTGTTTGAAGGGCGG 5488
          ......6910......6920......6930......6940......6950......6960......6970......6980......6990......7000......7010......7020......7030......7040......7050

Gaac_spg1 GAGATCCCTGAATTCCATTTTTCTGGTAAAGAAAATTAGAAGGATATGTAGTTAATTACGTTAATTGTCTGGATTTTATTTCAAATGTGTAGTACGTCTAGTTGCCTAACAATCCATTCATCATTCAGC--ATTTACCCCGCCACCCATT 4340
Gaac_spg2 ------------------------------------------------------------------------------------------------------------------------------------------------------ 4680
Gaac_spg3 GAGATCACTAAATTCCATCAGTTCC---AAGAAAATTGTGAGGAAATATAGTTAATTACATTATTTGGCTGCAATTAATTTTAAATGTGTAGTACGTCTTGTTGCCTAGCAATCCATTCATCAT-GGCT-AATTTACACGGCCACCCATT 4389
Gaac_spg4 GAGATCACACAATTTCATCAGTTCC---AGGAAACTTGTGAGGAAATATAGTTAATTACATTATTTGGCTGGAATTCATTTTAAATGTGTAGTACGTCTAGTTGCCTGGCAATCCATTCGTCATCGGCTCAATTTACCCGGCCACCCATT 4102
Gaac_spg5 GAGATTTCGGAATTCCATCTGTCTGGTAAAGAAAATTGAAAGCATATGTAGTTGATTACATT--TTGGCTGGATTTTCTTTCAAATGTGTAGTACGTCCAGTTGCCTAGCAATCCATTCATCAT-GGCT-AATTTACCCAGCCACCCATT 4454
Gaac_spg6 GAGATTTCGGAATTCCATCTGTCTGGTAAAGAAAATTGAAAGCATATGTAGTTGATTACATT--TTGGCTGGATTTTATTTCAAATGTGTAGTACGTCCAGTTGCCTAGCAATCCATTCATCATGGCTA--ATTTACCCAGCCACCCATT 3995
Gaac_spg7 GAGATCATGGAATTCCATCAGACTGGTAAAGACAATTGGGAATAAATCTAGTAAATTGCATCAATTGTCTGGACTTTAATTTGAATGAGTAAACCGTCTAGTTGCCTAGCAATCCAAAATGAGTAA------TTTACTTTCCCACCCATT 5632
          ......7060......7070......7080......7090......7100......7110......7120......7130......7140......7150......7160......7170......7180......7190......7200

                       13
Gaac_spg1 CATTT------ACTGAGTT-CTCTCCTGTAGGTCATGCTCAGGTTTACTGGGTGTCCTCCATGTTCG-TCCAGGTCCACACGACCATTGGTATAAACTTCCAAATTCAGCTCTCCCCTGAAATCCATCTGTTCATTGACGCACCTGACAC 4482
Gaac_spg2 ------------------------------------------------------------------------------------------------------------------------------------------------------ 4680
Gaac_spg3 AATTGAATTGAACTGAATTATCTCCCTGCAGGTAATGCTAAGATTTACTGGGTGTCCTCCACGTTCC-TCCAGGTCCACACAACCTCTGGGTTTTTTTTCAAATTCAGCTGTCCCCTGAAATCCAGCTGTTCATCGACGCACCCGACAA 4538
Gaac_spg4 ATTACTGAAT----------CTCTCCTGTAG-T-ATGCTAAGATTTACTGG-TGT-CTC-ACGT--C-T-CAGGTCCACACAAC-TCCGGTT--ATTTTTCAAATTCAGCTGTCCCCTGAAATCCAGCTGTTCATCGACGCACCTAACCA 4230
Gaac_spg5 AATTT------ATTGAGTT-CTCTCCTGTAGGTCATGCTCAGGTTTACTGGGTGTCCTCCATGTTCA-TCCAAGTCCACACGACCATTGGTATAAACTTCCAAATTCAGATGTCCCCTGAAATCCAGCTGTTCATCGACGCACCTGACAC 4596
Gaac_spg6 AATTT------ATTGAGTT-CTCTCCTGTAGGTCATGCTCAGGTTTACTGGGTGTCCTTCATTTTTCATCCAGGTCCACACAACCATTGGTATAAACTTCCAAATTCAGATGTCCCCTGAAATCCAGCTGTTCATCGACGCACCTGACAC 4138
Gaac_spg7 AATTTACTGAATTTT-------CTCCTTTAGATCATGCTCAGGTTTACTGGCTGTCTTCCATGTTCG-TCCAGGTCCACACGACCTGTGGTATACATTTCCAAATTCAGCTATCTCCTGAAATCCAGCTGTTCATCGACACACCCGACAA 5774
          ......7210......7220......7230......7240......7250......7260......7270......7280......7290......7300......7310......7320......7330......7340......7350

Gaac_spg1 CTCCAATGACAAGATTAAAG------GTGCGGACCACAGTCTACAGACAGGCCAAAATGTATTCACTTG---------TATTCACTT---------CCATTATGTCTTTTTTTAAATTAACTCTCAAGAAGAAAAAGA-------AAATC 4601
Gaac_spg2 ------------------------------------------------------------------------------------------------------------------------------------------------------ 4680
Gaac_spg3 CTCCAACTACAAGATTGAAG------GTGCAGACCATAGTCTAAAGACAGGCCAAAATATATTCCCTTCAA-------GATTTACTTGAAG-----GGAATATATTTCATATTTCATTCATTATCATTATGAATTAGC--------ATTT 4662
Gaac_spg4 ATCCAAGTCCGATGACAAGATTGAAGGTGCGGACCACAGTTTACAGACAGGCCCAAATATATTTGCTTCAGGATCTTCTTTTCACTTTGGGCTTCACTTTTATTATGACCTTTAAATTAGCGTTTAAGATTAAATGAATAAACTGAAATG 4380
Gaac_spg5 CTCCAATGACAAGATTAAAG------GTGCGGACCACAGTCTACAGACAGGCCAAAATATATTCACTGG---------CAATCACTT---------CCTTTATGACTTTTTAAAAATTAGCTCTCAAGATGAAAAAGA-------AAATC 4715
Gaac_spg6 CTCCAATGACAAAATTAAAG------GTGCGGACCACAGTCTACAGACAGGCCAAAATATATTCACTGG---------CAATCACTT---------CCTTTATGACTTTTTAAAAATTAGCTCTCAAGATGAAAAAGA-------AAATC 4257
Gaac_spg7 GTCCAATGACAAGATCAAAG------GTAAGGA---TCTGCGTCTTACACG-CAAAATATATTCATTTGGGGATCCACATTCGTTATGACATTAATTAAATTAGCTTAAATTAGTTGAAATGAATACAAGAATCAAGGAAAGAGTATACA 5914
          ......7360......7370......7380......7390......7400......7410......7420......7430......7440......7450......7460......7470......7480......7490......7500

                                                                            14
Gaac_spg1 AAT--AGGAAGTACATCATTTTTTCTGCGGCATGATCAAGTATGATTTGTAATCATAGCCAGTATGTTTTTTAGGTCTTTGTGGCAATAGCAACAGTGACACCACAGATGACTTCACCACCAACAGCGGGATCATTGAGAACTCAGCTAA 4749
Gaac_spg2 ------------------------------------------------------------------------------------------------------------------------------------------------------ 4680
Gaac_spg3 AATTAATAAACTAAA--ATGTTTTCTGTGACGTGATCCAGTATGATTTGTAATCATAG--ACTATGTCTTTTAGGTCTTTGTGGCAATAGCAACAATAACACCACAGATGACTTCATCACCAAGAGCAGGATCATTGAGAACTCAGCTGA 4808
Gaac_spg4 AATCAAGGAAATACATTATATTTTCTGTGGCATGATACAGTATGATTTGTAAACTTAGCCAGTATGTCTTTTAGGTCTTTGTGGTAATAGCAACAGGAACACCACAGATGACTTCACCACCGAAACCGGGTTCTTTCCGAACTCAGTTCA 4530
Gaac_spg5 AAT--AGAAAGTACATCATATTTTATGTGGCATGATCCAGTATGATTTGTAATCATAGCCAGTATGTCTTTTAGGTCTTTGTGGCAATAGCAACAGTGACACCACAGATGACTTCACCACCAACAGCGGGATCATTGAGAACTCAGCTAA 4863
Gaac_spg6 AAT--AGAAAGTACATCATATTTTATGTGGCATGATCCAGTATGATTTGTAATCATAGCCAGTATGTCTTTTAGGTCTTTGTGGCAATAGCAACAGTGACATCACAAATGACTTCACCACCAACAGCGGGATCAGCGAGAACTCAGCTAA 4405
Gaac_spg7 TTATTGATTTGTAATCAGAGGC----------------------------------------ATGTCTTTTAGGTCTTTGTGGCAATAGCAACAGTGACACCACAGATGACTTCACCGCCAACAGCGGGATCATTGAGAACTCAGCTCA 6023
          ......7510......7520......7530......7540......7550......7560......7570......7580......7590......7600......7610......7620......7630......7640......7650

Gaac_spg1 ACCATTTGCTATGTCCTGGAGTTTGCTTAATTGTTTCGGGAACATACCCACCACCTGCACCAACCTTGAGAATGGTACGAATTAATAATAT-----------------GTTTGAGATTCTCTCTGTATGTCCGTCCTTTGATTTTTCTTG 4882
Gaac_spg2 ------------------------------------------------------------------------------------------------------------------------------------------------------ 4680
Gaac_spg3 ACCATTTGCTCTCTCCTGGAGTTTGGGGAATTGTCAAGGGAACATACACACCAACTGCACCAAACGTGAATATGGTACGAATTAATAATATAAATATATTTGAAATATGTTTGAGATTCTCTCT----------CATCTG---------- 4938
Gaac_spg4 ACTATTTGCTCTCTCCTGGATTATGGGGAATTGTCAAGGGAACATACCCACTACCTGCACCAAACGTGAAAATGGTACGAATTTATAAT-------TTAAATGTGTATATTTGAGATTATCTCTGTATGCATGTTCTTTGATTTTTTTTG 4673
Gaac_spg5 ACCATTTGCTATGTCCTGGAGTTTGCTTAATTGTTTCGGGAACATACCCACCACCTGCACCAACCTTGAGAATGGTACGAATTAATAATATAAATGTGTTTCAAATATGTTTGAGATTCTCTCTTTATGTCCGTCCTTTGATTTTTCTTG 5013
Gaac_spg6 ACCATTTGCTATGTCCTGGAGTTTGCTTAATTGTTTCGGGAACATACCCACCACCTGCACCAACCTTGAGAATGGTAAAAATAAATAATATAAATGTGTTTCAAATATGATTGAGATTCTCTCCTTATGTCCGTCCTTTGATTTTTCTTG 4555
Gaac_spg7 ACCATTCGCTCTGTCGTGGAGTTTGGGTCATTGTCACGGGAACATACCCAGCAACTGCACCAACCGTGAAAATGGTACCAATCAATATTATAAATGTGTTAAAAATTCATTTTTTTCTGGGAACCAGTCATTTGATTG----------- 6161
          ......7660......7670......7680......7690......7700......7710......7720......7730......7740......7750......7760......7770......7780......7790......7800
```

```
                                    ********* *  * ****   *    **** ***                 *  *     * **       *      *     **          *    *
Gaac_spg1 AGAACTACCCATCCGATTAACAGAACACTTGCATGATGCTGAGGACCGCAGCGAATCAAGTGTCAAGATAATAGGATATATACGTTGGTAGTGCATATTTACACAGTGGTTTGTGTTTACTGTGCCATTGCTACTTTTTGCAATTTTGTA  5032
Gaac_spg2 --------------------------ACTTGCATGGTGCTGAGGACCGCAGGGAATCTAGTGTCGAGATTTAGGGATATTTTCATTGGTAGTGCATATTTACACAATGGGTTGTGTCTGCTGTGCCATTACTACTTTTTGCAATTATGTA  4804
Gaac_spg3 --AACTACTCATCCGATTGACAGAACACTTGCATGATTTTGAGGATGGCATCAAATCCAGTTTCAAGATAAGGGGATATATTCATGGGTAGTGCATATTTACACAGTAGGTTGTGTTTACTGTGCCATTGCTACTTAT------------  5074
Gaac_spg4 AGAACTACTCATTCAATTGACAGAACACTTGCATGATTTTGAGGACGACTTTGAATCCAGTTTTAAGAGAAGGGGATATATTCATGGGTAGTGCATATTTACACAGTAGGTTGAGTTTACTTTGCCATTGCTACGTATTGCAATTTTGTA  4823
Gaac_spg5 AGAACTACTCATCCGATTAACAGAACACTTGCATGATGCTGAGGACCGCAGCGAATCAAGTGTCAAGATAATAGGATATATACGTTGGTAGTGCATATTTACACAGTGGGTTGTGTTTACTGTGCCATTGCTACTTTTTGCAATTATGTA  5163
Gaac_spg6 AGAACTACTCATCCGATTAACAGAACACTTGCATGATGCTGAGGACCGCAGGGAATCAAGTGTCAAGATAATAGGATATATACGTTGGTAGTGCATATTTACACAGTGGGTTGTGTTTACTGTGCCATGGCTACTTTTTGCAATTATGTA  4705
Gaac_spg7 -------------------ACAGAACACTTGCATGATGCTAAGGACCGCACGGAATCCAGTCAGGTAGAAGCTCCTGAGATAATGGGATATATTCATTGGTAGTACATATTTACACAGTGGGTTATGTGTACTGTAGCTATTTGCTAGTT  6292
          ......7810......7820......7830......7840......7850......7860......7870......7880......7890......7900......7910......7920......7930......7940......7950

                *         *     *    *       *          *       *                       * *  *   *
Gaac_spg1 TGTCATAGCAGTTGTATAAATACCCAGGACAAAGAAGCGCAGCATCGAGTGTGAA-GGGGTACCAGAGCCCCAGCAATGGGCCTTTTCTTATATGGCACCT-----------------------TTTAGAAAT-----------------  5141
Gaac_spg2 TGTCAGAGCAGTTGTATTATTACCCAGGACAAGGAAGCGCAGTGTAGAATCTGAAAGCAGTACGAGAGGCTAAGCAATGGGCCTTTTCTAACATGGCACCT-------------------------------------------------  4905
Gaac_spg3 ---CCTAGCACTTGGATTATTACCCAGGAAAATGAAGCGCAGAATCGGGTGCGTA-GCAGTACCAGAGGCCCAGCAAGGGGCCTTTTCCTATATGGCACCTTTTATAAGGGGCACAAATCAAATATTGAGTGT-----------------  5203
Gaac_spg4 TGTCATAGCAGTTGGATTATTACCCAGGACAAGGAAGCACAG--TGGGATGTGTA-GCAGTACCAGAGGCCCAGCAATGGGCTTCTTCTTATATGGCACCT-----------------------TTTAGAAGG-----------------  4930
Gaac_spg5 TGTCATAGCAGATGTATTATTACCCAGGACAAGGAAGCGCAGCATCGAGTGTGAA-GCAGTACCAGAGCCCCAGCAACGGGCTTCTTCTTATATGGCACCT-----------------------TTTAGAAGG-----------------  5272
Gaac_spg6 TGTCATAGCAGTTGTATTATTACCCAGGACAAGGAAGTGCAGCATCGAGTGTGAA-GCGGTACCAGAGCCCCAGCAATGGGCTTCTTCTTATATGGCACCT-----------------------TTTAGAAGG-----------------  4814
Gaac_spg7 GTTGCAACTCTGCATGTCTTAGCGGTTGTATTTTTACCCAGAACAAGGAAGCACAATGTTGGGTGTGAAGCACTACCGGAGAATCAGCAATAGGCCATGTCATGTTGTTGTAGTGCTGGACCCAGGAGCAGAATTCATACAGAAAGGAAT  6442
          ......7960......7970......7980......7990......8000......8010......8020......8030......8040......8050......8060......8070......8080......8090......8100

Gaac_spg1 ------------------------------------------------------------------------------------------------------------------------------------------------------  5141
Gaac_spg2 ------------------------------------------------------------------------------------------------------------------------------------------------------  4905
Gaac_spg3 ------------------------------------------------------------------------------------------------------------------------------------------------------  5203
Gaac_spg4 ------------------------------------------------------------------------------------------------------------------------------------------------------  4930
Gaac_spg5 ------------------------------------------------------------------------------------------------------------------------------------------------------  5272
Gaac_spg6 ------------------------------------------------------------------------------------------------------------------------------------------------------  4814
Gaac_spg7 GGTTGGCAGACTGGGAGCAGGTTGATTCCACATGGCAGGACTGACAATCTGGCAATGACCGGAGTGGAGGGCAAGAGCATATCAAGCATGGCACTTGAGTAGCCAGATTCTGCTGGTCAATTGGTAGGAAGGGCACTCCTCAGACCATGA  6592
          ......8110......8120......8130......8140......8150......8160......8170......8180......8190......8200......8210......8220......8230......8240......8250

Gaac_spg1 ------------------------------------------------------------------------------------------------------------------------------------------------------  5141
Gaac_spg2 ------------------------------------------------------------------------------------------------------------------------------------------------------  4905
Gaac_spg3 ------------------------------------------------------------------------------------------------------------------------------------------------------  5203
Gaac_spg4 ------------------------------------------------------------------------------------------------------------------------------------------------------  4930
Gaac_spg5 ------------------------------------------------------------------------------------------------------------------------------------------------------  5272
Gaac_spg6 ------------------------------------------------------------------------------------------------------------------------------------------------------  4814
Gaac_spg7 GCGGGGGGGCCCAGGCAGCCGACCAGGCTTGTCCGGATGAGTCTAGTGAAACTCCCTGAGTTGGGATCCAAGATGAAGGACCGAGAGACCCACCACCTCTGGACTATAGCCCTCCCAGTCAACCAAGATACAAGAACCCCTGCCACAGCA  6742
          ......8260......8270......8280......8290......8300......8310......8320......8330......8340......8350......8360......8370......8380......8390......8400

Gaac_spg1 ------------------------------------------------------------------------------------------------------------------------------------------------------  5141
Gaac_spg2 ------------------------------------------------------------------------------------------------------------------------------------------------------  4905
Gaac_spg3 ------------------------------------------------------------------------------------------------------------------------------------------------------  5203
Gaac_spg4 ------------------------------------------------------------------------------------------------------------------------------------------------------  4930
Gaac_spg5 ------------------------------------------------------------------------------------------------------------------------------------------------------  5272
Gaac_spg6 ------------------------------------------------------------------------------------------------------------------------------------------------------  4814
Gaac_spg7 ATGTACATCCAAGAGCTGCCAAACCGAGAAGGCAGGCGAAGGGGGTCGAGTTGGAAGAGGGCAGACGACTGGCGGACACTTGTTTTGACTATGAAACATGGAATGTGGGATGGACTCTTATGGTGAGAGGCAGTTTCAGCCCAGGAGCTG  6892
          ......8410......8420......8430......8440......8450......8460......8470......8480......8490......8500......8510......8520......8530......8540......8550

Gaac_spg1 ------------------------------------------------------------------------------------------------------------------------------------------------------  5141
Gaac_spg2 ------------------------------------------------------------------------------------------------------------------------------------------------------  4905
Gaac_spg3 ------------------------------------------------------------------------------------------------------------------------------------------------------  5203
Gaac_spg4 ------------------------------------------------------------------------------------------------------------------------------------------------------  4930
Gaac_spg5 ------------------------------------------------------------------------------------------------------------------------------------------------------  5272
Gaac_spg6 ------------------------------------------------------------------------------------------------------------------------------------------------------  4814
Gaac_spg7 GATTTGGATTGAGCGGAGTAGGGCAGAGTGACTGTTTCTCTAAATCTTACAGCAGTGGCAGAGGTGCATTTGAACAGAACAGCCATCTCATTCTCTTGACAGGGAAATAGGTGTGGCCAACAATAGACTCAGATGGGGATAAACCTGTGG  7042
          ......8560......8570......8580......8590......8600......8610......8620......8630......8640......8650......8660......8670......8680......8690......8700

Gaac_spg1 ------------------------------------------------------------------------------------------------------------------------------------------------------  5141
Gaac_spg2 ------------------------------------------------------------------------------------------------------------------------------------------------------  4905
Gaac_spg3 ------------------------------------------------------------------------------------------------------------------------------------------------------  5203
Gaac_spg4 ------------------------------------------------------------------------------------------------------------------------------------------------------  4930
Gaac_spg5 ------------------------------------------------------------------------------------------------------------------------------------------------------  5272
Gaac_spg6 ------------------------------------------------------------------------------------------------------------------------------------------------------  4814
Gaac_spg7 CAGCGCTGGTCAAGCAATTATGGGCGTATTCAACCCATGGATGTGAGCGATGGGACTGGTGGTGGTTACACACTGTAGAGCAGCATTTATCCAGCACCAACAGTGGATGAAGATGGTTTGGTTATACCAGCAGTCAGTGACTCCTCGATA  7192
          ......8710......8720......8730......8740......8750......8760......8770......8780......8790......8800......8810......8820......8830......8840......8850

Gaac_spg1 ------------------------------------------------------------------------------------------------------------------------------------------------------  5141
Gaac_spg2 ------------------------------------------------------------------------------------------------------------------------------------------------------  4905
Gaac_spg3 ------------------------------------------------------------------------------------------------------------------------------------------------------  5203
Gaac_spg4 ------------------------------------------------------------------------------------------------------------------------------------------------------  4930
Gaac_spg5 ------------------------------------------------------------------------------------------------------------------------------------------------------  5272
Gaac_spg6 ------------------------------------------------------------------------------------------------------------------------------------------------------  4814
Gaac_spg7 TAGTTCTCCATGGCCTCCCTCTCTGGAGGTACATGTTTTAAAGCCTGCTAGTGGGAAGAGGAGCACCGGAAAGGAGATCAATGGCACAGTCATGAGGACAGAGAGGAGGCAAGGAGAGAGCGAGCTCTTTACTGAACACCAAACATAGAT  7342
          ......8860......8870......8880......8890......8900......8910......8920......8930......8940......8950......8960......8970......8980......8990......9000

Gaac_spg1 ------------------------------------------------------------------------------------------------------------------------------------------------------  5141
Gaac_spg2 ------------------------------------------------------------------------------------------------------------------------------------------------------  4905
Gaac_spg3 ------------------------------------------------------------------------------------------------------------------------------------------------------  5203
Gaac_spg4 ------------------------------------------------------------------------------------------------------------------------------------------------------  4930
Gaac_spg5 ------------------------------------------------------------------------------------------------------------------------------------------------------  5272
Gaac_spg6 ------------------------------------------------------------------------------------------------------------------------------------------------------  4814
Gaac_spg7 CATGATACTTAGCAGGTATGGTGGACATATCTGAAGGCAAAGAGCTCGGTGGTGATATGGTGACATCTGCCGGGGACAGGGCTGACTGTCCATGGCAGAGACGGAGAGAGTGACAAAATGAGCTCCAGCCAAGGATTCTCCCTTCAGTAC  7492
          ......9010......9020......9030......9040......9050......9060......9070......9080......9090......9100......9110......9120......9130......9140......9150

Gaac_spg1 ------------------------------------------------------------------------------------------------------------------------------------------------------  5141
Gaac_spg2 ------------------------------------------------------------------------------------------------------------------------------------------------------  4905
Gaac_spg3 ------------------------------------------------------------------------------------------------------------------------------------------------------  5203
Gaac_spg4 ------------------------------------------------------------------------------------------------------------------------------------------------------  4930
Gaac_spg5 ------------------------------------------------------------------------------------------------------------------------------------------------------  5272
Gaac_spg6 ------------------------------------------------------------------------------------------------------------------------------------------------------  4814
Gaac_spg7 ATGTGTGGGTTGTGTGTCCATAACCAAGGCTGACCCAAAACCTGAAGGGAGTTAGGGAAGGCAGACTGGGAGAAGGGTGTTTCCACATGGCAGGACTGACAATCTGGCAAAGACTGGAATGGAGGGCTGGAGTGTATCAAGCATGGTGCT  7642
          ......9160......9170......9180......9190......9200......9210......9220......9230......9240......9250......9260......9270......9280......9290......9300

Gaac_spg1 -------------------------------------------------------------------------------------------------GGCACTG----CACTAGTGTGTATAAATTAAG----ACTCAATATTTGATTTG  5186
Gaac_spg2 ------------------------------------------------------------------------------------------------------------------------------------------------------  4905
Gaac_spg3 -------------------------------------------------------------------------------------------------GACTTTATACACACGAGTGCGTGTGTATAAAGTCACACTCAATATTTGATTTG  5256
Gaac_spg4 -------------------------------------------------------------------------------------------------GCCACTG----CACTAGTGTGT----CAC-------ACTCAATATTTGATTTG  4968
Gaac_spg5 -------------------------------------------------------------------------------------------------GGCACTG----CACTAGTGTGTATAACACTAGTCCCACTCAATATTTGATTTG  5321
Gaac_spg6 -------------------------------------------------------------------------------------------------GGCACTG----CACTAGTGTGTACAAAGTCCC----ACTCAATATTTGATTTG  4859
Gaac_spg7 TGAGTATTGGATGTGTAACAGCTGTGCCGGTCAATTGGTAGCAGGGGCACAGCTCTGGTCATCACAGGCCATTTTTGATTAGGCACATTTGTGAAAGGGCACTG----CATTAGTGTTTATAAATTCAC----AATCAATATTTGATTTG  7784
          ......9310......9320......9330......9340......9350......9360......9370......9380......9390......9400......9410......9420......9430......9440......9450

Gaac_spg1 TCTACCGATCAGGGCTGAAAAGTCATTTAAAATATTCTGCATCAAATCA--------ACTTG----------------------------------------------------------------------------------------  5240
Gaac_spg2 ------------------------------------------------------------------------------------------------------------------------------------------------------  4905
Gaac_spg3 TCTACCAATCAGGGTTGAGAAGTAATTTAGAAAATTATACAAATTATTATTATTAAGACTTT----------------------------------------------------------------------------------------  5318
Gaac_spg4 TCTACCGATCAGGGCTGAGAAGTCATTTAGAATATTATACAAATTATAAG-------ACTTT----------------------------------------------------------------------------------------  5023
Gaac_spg5 TCTACCGATCAGGGCTGAGAAGTCATTTAAAATATTCGACATCATATCA--------ACATT----------------------------------------------------------------------------------------  5375
Gaac_spg6 TCTACCGATCAGGGCTGAGAAGTCATTTAAAATATTCGACATCATATCA--------ACATGTCAGGGTTGCGGGTGGAAGGCAGGACTCAGACGCAGAGTTTTCCAATACAAAAAGACTTTAATAGTCAATGGTCAAAAATCCAAGGGG  5001
Gaac_spg7 TCTACCGGGATGACAAGTTACTTAAAAGAATATAACATCAAATCATACA--------ACTTT----------------------------------------------------------------------------------------  7838
          ......9460......9470......9480......9490......9500......9510......9520......9530......9540......9550......9560......9570......9580......9590......9600

Gaac_spg1 ------------------------------------------------------------------------------------------------------------------------------------------------------  5240
Gaac_spg2 ------------------------------------------------------------------------------------------------------------------------------------------------------  4905
Gaac_spg3 ------------------------------------------------------------------------------------------------------------------------------------------------------  5318
Gaac_spg4 ------------------------------------------------------------------------------------------------------------------------------------------------------  5023
Gaac_spg5 ------------------------------------------------------------------------------------------------------------------------------------------------------  5375
Gaac_spg6 GCAAAAACACACAGGCATGAACTATGGGTATCCAAGACAAAGACAGGACATGCGTGGCTAAAGACAATGACGCGACCAGTGACACAGGAGACACACGGCTTAAATACACAAGGGAGGTGCAGGTGATTGGACACAGGTGGAAACTATTAG  5151
Gaac_spg7 ------------------------------------------------------------------------------------------------------------------------------------------------------  7838
          ......9610......9620......9630......9640......9650......9660......9670......9680......9690......9700......9710......9720......9730......9740......9750
```

```
Gaac_spg1 ------------------------------------------------------------------------------------------------------------------------------------------------------ 5240
Gaac_spg2 ------------------------------------------------------------------------------------------------------------------------------------------------------ 4905
Gaac_spg3 ------------------------------------------------------------------------------------------------------------------------------------------------------ 5318
Gaac_spg4 ------------------------------------------------------------------------------------------------------------------------------------------------------ 5023
Gaac_spg5 ------------------------------------------------------------------------------------------------------------------------------------------------------ 5375
Gaac_spg6 ACGATCACAGGGGATGACGGGACAAGGCAGGAAGTGAAGTTACCCGGGGACACGAGTGGCAGAAAACTACAAAATACAACAGGAAGTGAAACCACACCGTGACAGTACCCCCCCCTCAAGGACCGACTTCCAGGCGGCTCACACTAGAGC 5301
Gaac_spg7 ------------------------------------------------------------------------------------------------------------------------------------------------------ 7838
          ......9760......9770......9780......9790......9800......9810......9820......9830......9840......9850......9860......9870......9880......9890......9900

Gaac_spg1 ------------------------------------------------------------------------------------------------------------------------------------------------------ 5240
Gaac_spg2 ------------------------------------------------------------------------------------------------------------------------------------------------------ 4905
Gaac_spg3 ------------------------------------------------------------------------------------------------------------------------------------------------------ 5318
Gaac_spg4 ------------------------------------------------------------------------------------------------------------------------------------------------------ 5023
Gaac_spg5 ------------------------------------------------------------------------------------------------------------------------------------------------------ 5375
Gaac_spg6 AAAACAAGGGGTGGGGGGGCAGGGAAACCAGAGACGTTGGTCGGACCACCCGGGAAACTCTGGCGGGCGGCCCGGCGGGTGGCCAGACGAGCGGGGAGCCTCTGGGGGTCGGCCCGGCGGGCGGTCACCAAGCCGGCTCCGGAGCGGCGA 5451
Gaac_spg7 ------------------------------------------------------------------------------------------------------------------------------------------------------ 7838
          ......9910......9920......9930......9940......9950......9960......9970......9980......9990.....10000.....10010.....10020.....10030.....10040.....10050

Gaac_spg1 ------------------------------------------------------------------------------------------------------------------------------------------------------ 5240
Gaac_spg2 ------------------------------------------------------------------------------------------------------------------------------------------------------ 4905
Gaac_spg3 ------------------------------------------------------------------------------------------------------------------------------------------------------ 5318
Gaac_spg4 ------------------------------------------------------------------------------------------------------------------------------------------------------ 5023
Gaac_spg5 ------------------------------------------------------------------------------------------------------------------------------------------------------ 5375
Gaac_spg6 CTGCTAGGGCACTGAACGGATCTATTATGCCTAGCTCATGGGACTATGAAAATCTCAGGTGTCGACGTCAGCAGATCTATGAACACATATCACGTACTCCATTTGTAGCCATATGTAGCTCTGGCGACACGGAAGACATACGCGTTTTGT 5601
Gaac_spg7 ------------------------------------------------------------------------------------------------------------------------------------------------------ 7838
          .....10060.....10070.....10080.....10090.....10100.....10110.....10120.....10130.....10140.....10150.....10160.....10170.....10180.....10190.....10200

Gaac_spg1 ------------------------------------------------------------------------------------------------------------------------------------------------------ 5240
Gaac_spg2 ------------------------------------------------------------------------------------------------------------------------------------------------------ 4905
Gaac_spg3 ------------------------------------------------------------------------------------------------------------------------------------------------------ 5318
Gaac_spg4 ------------------------------------------------------------------------------------------------------------------------------------------------------ 5023
Gaac_spg5 ------------------------------------------------------------------------------------------------------------------------------------------------------ 5375
Gaac_spg6 ACCGGCCGTGCCGAGCACACGGCAACACCTTGCGCGGTTGTCGGCGTCGGCACTGGGCACTGGAAGACAACCNNNNNNNNNNNNNNNNNNNNNNNNNNNNNNNNNNNNNNNNNNNNNNNNNNNNNNNNNNNNNNNNNNNNNNNNNNNNNN 5751
Gaac_spg7 ------------------------------------------------------------------------------------------------------------------------------------------------------ 7838
          .....10210.....10220.....10230.....10240.....10250.....10260.....10270.....10280.....10290.....10300.....10310.....10320.....10330.....10340.....10350

Gaac_spg1 ------------------------------------------------------------------------------------------------------------------------------------------------------ 5240
Gaac_spg2 ------------------------------------------------------------------------------------------------------------------------------------------------------ 4905
Gaac_spg3 ------------------------------------------------------------------------------------------------------------------------------------------------------ 5318
Gaac_spg4 ------------------------------------------------------------------------------------------------------------------------------------------------------ 5023
Gaac_spg5 ------------------------------------------------------------------------------------------------------------------------------------------------------ 5375
Gaac_spg6 NNNNNNNNNNNNNNNNNNNNNNNNNNNNNNNNNNNNNNNNNNNNNNNNNNNNNNNNNNNNNNNNNNNNNNNNNNNNNNNNNNNNNNNNNNNNNNNNNNNNNNNNNNNNNNNNNNNNNNNNNNNNNNNNNNNNNNNNNNNNNNNNNNNNNNNN 5901
Gaac_spg7 ------------------------------------------------------------------------------------------------------------------------------------------------------ 7838
          .....10360.....10370.....10380.....10390.....10400.....10410.....10420.....10430.....10440.....10450.....10460.....10470.....10480.....10490.....10500

Gaac_spg1 ------------------------------------------------------------------------------------------------------------------------------------------------------ 5240
Gaac_spg2 ------------------------------------------------------------------------------------------------------------------------------------------------------ 4905
Gaac_spg3 ------------------------------------------------------------------------------------------------------------------------------------------------------ 5318
Gaac_spg4 ------------------------------------------------------------------------------------------------------------------------------------------------------ 5023
Gaac_spg5 ------------------------------------------------------------------------------------------------------------------------------------------------------ 5375
Gaac_spg6 NNNNNNNNNNNNNNNNNNNNNNNNNGTTTGAGAACCCGCCGCTGGCACACGAGAGACCTTCCACCACAGACGCAAGTTGACATCCTGTAAGTACCTCAGCTGGTTTAGTAGACATCAGACCCAAGAGCTCTCCAGGAGCTCTGAGCCTCA 6051
Gaac_spg7 ------------------------------------------------------------------------------------------------------------------------------------------------------ 7838
          .....10510.....10520.....10530.....10540.....10550.....10560.....10570.....10580.....10590.....10600.....10610.....10620.....10630.....10640.....10650

Gaac_spg1 ------------------------------------------------------------------------------------------------------------------------------------------------------ 5240
Gaac_spg2 ------------------------------------------------------------------------------------------------------------------------------------------------------ 4905
Gaac_spg3 ------------------------------------------------------------------------------------------------------------------------------------------------------ 5318
Gaac_spg4 ------------------------------------------------------------------------------------------------------------------------------------------------------ 5023
Gaac_spg5 ------------------------------------------------------------------------------------------------------------------------------------------------------ 5375
Gaac_spg6 ATGCTCTCATGTGACTCTGAAAGACTGTCGCAACCAGACCAAAGGTTGACTTCTGATTGAGTATGGTTTACATCAACCTCCTTGGCGTTATACAGTACAGATAGAGTCACTTCATATTCGTCTCCCCGTCGGTAGACGTTCTTCTGAGTT 6201
Gaac_spg7 ------------------------------------------------------------------------------------------------------------------------------------------------------ 7838
          .....10660.....10670.....10680.....10690.....10700.....10710.....10720.....10730.....10740.....10750.....10760.....10770.....10780.....10790.....10800

Gaac_spg1 ------------------------------------------------------------------------------------------------------------------------------------------------------ 5240
Gaac_spg2 ------------------------------------------------------------------------------------------------------------------------------------------------------ 4905
Gaac_spg3 ------------------------------------------------------------------------------------------------------------------------------------------------------ 5318
Gaac_spg4 ------------------------------------------------------------------------------------------------------------------------------------------------------ 5023
Gaac_spg5 ------------------------------------------------------------------------------------------------------------------------------------------------------ 5375
Gaac_spg6 GTTTTAGTTTCTCCACAGAAAACACTGCATTTTTATCAGCTGTTAATTTGGCTAATTTTATTTGACCGTATGTGAAGATCCAGCTTCTTCGGGTGAGACTGAATCTGCTGATCTTCTCCCTTGATTCAACAAAGTAGTATTAGGTCACTA 6351
Gaac_spg7 ------------------------------------------------------------------------------------------------------------------------------------------------------ 7838
          .....10810.....10820.....10830.....10840.....10850.....10860.....10870.....10880.....10890.....10900.....10910.....10920.....10930.....10940.....10950

Gaac_spg1 ------------------------------------------------------------------------------------------------------------------------------------------------------ 5240
Gaac_spg2 ------------------------------------------------------------------------------------------------------------------------------------------------------ 4905
Gaac_spg3 ------------------------------------------------------------------------------------------------------------------------------------------------------ 5318
Gaac_spg4 ------------------------------------------------------------------------------------------------------------------------------------------------------ 5023
Gaac_spg5 ------------------------------------------------------------------------------------------------------------------------------------------------------ 5375
Gaac_spg6 ATGACCCAAAAGTCATTAGAGAATCCAGACTTTCCCTGGAGTGGAAGCAGTGGGAGGATCTTCTCGCATCACTGATCAGCCGGTCGTTGTCGGGTGTCTCCCACGGATTAGGCATCCTGAACCAGTCGGGTCGGGGCCTTGGAGGTCGGC 6501
Gaac_spg7 ------------------------------------------------------------------------------------------------------------------------------------------------------ 7838
          .....10960.....10970.....10980.....10990.....11000.....11010.....11020.....11030.....11040.....11050.....11060.....11070.....11080.....11090.....11100

Gaac_spg1 ------------------------------------------------------------------------------------------------------------------------------------------------------ 5240
Gaac_spg2 ------------------------------------------------------------------------------------------------------------------------------------------------------ 4905
Gaac_spg3 ------------------------------------------------------------------------------------------------------------------------------------------------------ 5318
Gaac_spg4 ------------------------------------------------------------------------------------------------------------------------------------------------------ 5023
Gaac_spg5 ------------------------------------------------------------------------------------------------------------------------------------------------------ 5375
Gaac_spg6 ACCTGTCCTGGTCGGGTCGGGGGCATGGAACGTGGGGCCGGACTGGTCGGGTCGGGGGCATGGAACGTGGTGCCGGTGCTGGTCGGGTCGGGGGCATGGAAGGTGGGGCCGGTACTGGTCGGGTCGGGGCCATGGAACGTGGCGCCGGAA 6651
Gaac_spg7 ------------------------------------------------------------------------------------------------------------------------------------------------------ 7838
          .....11110.....11120.....11130.....11140.....11150.....11160.....11170.....11180.....11190.....11200.....11210.....11220.....11230.....11240.....11250

Gaac_spg1 ------------------------------------------------------------------------------------------------------------------------------------------------------ 5240
Gaac_spg2 ------------------------------------------------------------------------------------------------------------------------------------------------------ 4905
Gaac_spg3 ------------------------------------------------------------------------------------------------------------------------------------------------------ 5318
Gaac_spg4 ------------------------------------------------------------------------------------------------------------------------------------------------------ 5023
Gaac_spg5 ------------------------------------------------------------------------------------------------------------------------------------------------------ 5375
Gaac_spg6 CTGGTCGGGTCGGGGGCATGGAACGTGGTGCTGGTACCGGGGGCATGGATCGTGGCGCTGGCTCGGGGGTCGGGGACATGGATCGTGGCACTGGCTCGGGGGTCGGGGACATGGATCGTGGCACTGGCTCGGGTGTCGGGGGGCATGGAT 6801
Gaac_spg7 ------------------------------------------------------------------------------------------------------------------------------------------------------ 7838
          .....11260.....11270.....11280.....11290.....11300.....11310.....11320.....11330.....11340.....11350.....11360.....11370.....11380.....11390.....11400

Gaac_spg1 ------------------------------------------------------------------------------------------------------------------------------------------------------ 5240
Gaac_spg2 ------------------------------------------------------------------------------------------------------------------------------------------------------ 4905
Gaac_spg3 ------------------------------------------------------------------------------------------------------------------------------------------------------ 5318
Gaac_spg4 ------------------------------------------------------------------------------------------------------------------------------------------------------ 5023
Gaac_spg5 ------------------------------------------------------------------------------------------------------------------------------------------------------ 5375
Gaac_spg6 CGTGGCACTGGCTCGGGGGTCGGGGGGCATGGATCGTGGCACTGGCTCGGGGGTCATGGGCTTGGGTCGGGAGGCCGGGACGGGAATCTGCTGCGGCCCAGCGCGGGACATCTTGCGCTGCGACCGAGCGGGGTCGGGACGTCGCTGCGG 6951
Gaac_spg7 ------------------------------------------------------------------------------------------------------------------------------------------------------ 7838
          .....11410.....11420.....11430.....11440.....11450.....11460.....11470.....11480.....11490.....11500.....11510.....11520.....11530.....11540.....11550

Gaac_spg1 ------------------------------------------------------------------------------------------------------------------------------------------------------ 5240
Gaac_spg2 ------------------------------------------------------------------------------------------------------------------------------------------------------ 4905
Gaac_spg3 ------------------------------------------------------------------------------------------------------------------------------------------------------ 5318
Gaac_spg4 ------------------------------------------------------------------------------------------------------------------------------------------------------ 5023
Gaac_spg5 ------------------------------------------------------------------------------------------------------------------------------------------------------ 5375
Gaac_spg6 CCCAGCGCTGGACATCTTGCGCTGCGACCGAGCGGGGTCGGGACGTCGCTGCGGCCCAGCGCTGGACATAGTGCGCTGCGGCCGAGCGTGGGGAGCATAGGTGGCCTGTAGTCGGACCGCAAGGTCCATTACCCGCTCCCAGTGCGAATC 7101
Gaac_spg7 ------------------------------------------------------------------------------------------------------------------------------------------------------ 7838
          .....11560.....11570.....11580.....11590.....11600.....11610.....11620.....11630.....11640.....11650.....11660.....11670.....11680.....11690.....11700
```

```
Gaac_spg1 ------------------------------------------------------------------------------------------------------------------------------------------------------  5240
Gaac_spg2 ------------------------------------------------------------------------------------------------------------------------------------------------------  4905
Gaac_spg3 ------------------------------------------------------------------------------------------------------------------------------------------------------  5318
Gaac_spg4 ------------------------------------------------------------------------------------------------------------------------------------------------------  5023
Gaac_spg5 ------------------------------------------------------------------------------------------------------------------------------------------------------  5375
Gaac_spg6 GCCGCCAGACGACCACGAAATGGTCTCCTCCTCTGGGGACCATGTGGGGGGCGGCGGATGGTGACCCAGATGCCTACTGAGGGCTCCAGATGGGGAGACGGAGTAGTACAAGTACCCAGCTGGAACCCCCGGGAGGACCGTTCCCCCATT  7251
Gaac_spg7 ------------------------------------------------------------------------------------------------------------------------------------------------------  7838
          .....11710.....11720.....11730.....11740.....11750.....11760.....11770.....11780.....11790.....11800.....11810.....11820.....11830.....11840.....11850

Gaac_spg1 ------------------------------------------------------------------------------------------------------------------------------------------------------  5240
Gaac_spg2 ------------------------------------------------------------------------------------------------------------------------------------------------------  4905
Gaac_spg3 ------------------------------------------------------------------------------------------------------------------------------------------------------  5318
Gaac_spg4 ------------------------------------------------------------------------------------------------------------------------------------------------------  5023
Gaac_spg5 ------------------------------------------------------------------------------------------------------------------------------------------------------  5375
Gaac_spg6 ACCAGCAGCCCGCTGGAGACTCCGTGGACCGCCCATTGCCAGACGGGTTCCCCTGAACTCGTCCAGGGGAAAAAACTCTGCTGAGTCCTTTCTAGGTCGCGTCATTCTGTCAGGGTTGCGGGTGGAAGGCAGGACTCAGACGCAGAGTTT  7401
Gaac_spg7 ------------------------------------------------------------------------------------------------------------------------------------------------------  7838
          .....11860.....11870.....11880.....11890.....11900.....11910.....11920.....11930.....11940.....11950.....11960.....11970.....11980.....11990.....12000

Gaac_spg1 ------------------------------------------------------------------------------------------------------------------------------------------------------  5240
Gaac_spg2 ------------------------------------------------------------------------------------------------------------------------------------------------------  4905
Gaac_spg3 ------------------------------------------------------------------------------------------------------------------------------------------------------  5318
Gaac_spg4 ------------------------------------------------------------------------------------------------------------------------------------------------------  5023
Gaac_spg5 ------------------------------------------------------------------------------------------------------------------------------------------------------  5375
Gaac_spg6 TCCAATACAAAAAGACTTTAATAGTCAATGGTCAAAAATACAAGGGGGCAAAAACACACAGGCATGAACTATGGGTATCCAAGACAAAGACAGGACATGCGTGGCTAAAGACAATGACGCGACCAGTGACACAGGAGACACACGGCTTAA  7551
Gaac_spg7 ------------------------------------------------------------------------------------------------------------------------------------------------------  7838
          .....12010.....12020.....12030.....12040.....12050.....12060.....12070.....12080.....12090.....12100.....12110.....12120.....12130.....12140.....12150

Gaac_spg1 ------------------------------------------------------------------------------------------------------------------------------------------------------  5240
Gaac_spg2 ------------------------------------------------------------------------------------------------------------------------------------------------------  4905
Gaac_spg3 ------------------------------------------------------------------------------------------------------------------------------------------------------  5318
Gaac_spg4 ------------------------------------------------------------------------------------------------------------------------------------------------------  5023
Gaac_spg5 ------------------------------------------------------------------------------------------------------------------------------------------------------  5375
Gaac_spg6 ATACACAAGGGAGGTGCAGGTGATTGGACACAGGTGGAAACTATTAGACGATCACAGGGGATGACGGGACAAGGCAGGAAGTGAAGTTACCCGGGGACACGAGTGGCAGAAAACTACAAAATACAACAGGAAGTGAAACCACACCGTGAC  7701
Gaac_spg7 ------------------------------------------------------------------------------------------------------------------------------------------------------  7838
          .....12160.....12170.....12180.....12190.....12200.....12210.....12220.....12230.....12240.....12250.....12260.....12270.....12280.....12290.....12300

                                                                                                                              15
                  * ** ***  *  * **    *   * *      **      *  *   *      *      ****  * **   **** **  * * ***** ************** *  **** *** *******  **
Gaac_spg1 --------GGTTTTAAAACATTGAAACATGTTGATAACCTTGCCTGTGATATAATACTAATACACAAGGTCAA----TAAAATGCAATAACGATTTAAATATTTTTTTTTGGTAGAGAATTATGCTCATGAAAAGTGTGCAGTGTTAAAC  5378
Gaac_spg2 --------ATTTTTTATACCTTGGTATTTGTTCACTAGCTTGCTTTTGAAATTATATCAAAATGGAATCTAAA----ATAAATGCAATAATGATTTCAATATGTATTTTT-GTAGAGAATTATGCTCATAAAAAGTGTTCAGTGTTGAAC  5042
Gaac_spg3 --------GGTTTTAAAACATTGAAATTTGTTCTCTAGCTTGCTTGTGATATGATACCAACATGCAAACCAAAGGTAAAAAATGCATTAATGATTTCAATATGTATTTTT-GTAGAGAATTATGCTCATGAAAAGTGTGCAGTGTTGAAC  5459
Gaac_spg4 --------AGTTTTAAAACATTGAAATTTGTTGACTAGCTTACTTGTGATATGATACAAATATGCAAGCCAGAGATATAAAATTCATTA---ATTTCAA--TGTGTTTTT-GTAGAGAATTATGCTCATGAAAAGTGTGCAGTGTTGAAC  5159
Gaac_spg5 --------GGTTTTAAAACATTGAAATTTGTTGATAACCTTGCTTGTGGTATGATACCAATATGCAAGGTCAA----TAAAATGCATTAATGATTTAAATATCTTTTTTTTGTAGAGAATTATGCTTACGAAAAATGTGCAGTGTTGAAC  5513
Gaac_spg6 ACAACATTGGTTTTAAAACATTGAAATTTGTTGATAACCTTGCTTGTGGTATGATACCAATATGCAAGGTCAA----TAAAATGCATTAATGATTTAAATATCTTTTTTTTGTAGAGAATTATGCTTATGAAAAGTGTGCAGTGTTGAAC  7847
Gaac_spg7 --------------AAAACGTTGTCATTT-TTCA-TATGATACC-----AATG---CAAGCAAGAAGTTTAAAG-----AAATGTATTAATGATTTCAATATGTATTTTT-GTAGAGAATTATGCTCATGAAAAGTGTGCAGTGTTACAC  7958
          .....12310.....12320.....12330.....12340.....12350.....12360.....12370.....12380.....12390.....12400.....12410.....12420.....12430.....12440.....12450

                                                                                                                                                16
          ******  **** * *****  ** ********************  ********    *******   *  * ******** ****   ** ** * **   *  *** *  **   ** ** *  * *  * ***  **** ******
Gaac_spg1 CAACCAACTGGGATATTTGCTAAGTGCCACCCTCATATCCCAACTGATTACTACTACACGGTAACAAGATTATT-CCCCATACTAAATTCTTCATAAATTTGAGAATTTCAGAAATTGACCTAATTATTACTTATTTC-CAGGCTTGCAT  5526
Gaac_spg2 CAACCAAATGGGATATTTGCTCAGTGCCACCCTCATATCCCAACTGAATACTACTACACGGTAACAAGATTTGTGCCCCATACTAAATCCTTTATAAGTTCGGGAGTTTTAGAAATTAACATATTACTTACTTATTTC-CAGGCTTGCAT  5191
Gaac_spg3 CAACCAACTGGGGTATTTGCCCAGGGCCACCCTCATATCCCAACTGATTACTACTATGAGGTAACAAGCGTTTTGCCCCATACCAAATCCTTTATAAGTTTGGGAGTTTAACAAAATAACATATTAATTACTTTTTTTACAGGCTTGCAT  5609
Gaac_spg4 CAACCAACTGGGGTATTTGCCCAGGGCCACCCTCATATCCCAACTGCTTACTACTATAAGGTAACAAGCGTTTTGCCCCATACCAAATCCTTTATAAGTTTGGGAGTTTAAGAAAATAACATATTAATTACTTTTTTTACAGGCTTGCAT  5309
Gaac_spg5 CAACCAACTGGGATATTTGCTAAGTGCCACCCTCATATCCCAACTGATTACTACTACACGGTAACAAGATTTTTTCCCCATACCAAATCCTTTATAAATTTGTGAGTTTCAGAAATTAACCTAATTATTACTTATTTC-CAGGCTTGCAT  5662
Gaac_spg6 CAACCAGCTGGGATATTTGCTAAGTGCCACCCTCATATCCCAACTGATTACTACTACACTGTAACAAGATTTTTTCCCCATACCAAATCTTTTATGAATTTGTGAGTTTCAGAAATTAACCTAATTATAAATTATTTC-CAGGATTGCAT  7996
Gaac_spg7 CAACCAACTGGGATGTTTGCTAAGTGCCACCCTCATATCCCAACTGATTACTACTACACGGTAACAATACTTTTGCCCCATACCAAATCCTTTATAAGTTTGGGGTTTCAGGAATCAACATATTCATTATGTATTTC-CAGGCTTGCAT  8107
          .....12460.....12470.....12480.....12490.....12500.....12510.....12520.....12530.....12540.....12550.....12560.....12570.....12580.....12590.....12600

           *************** ***     **** ** * *** * * *********** ******** ****** ******         ******** *** * ******* ** *  **  ** **  ** ****** **** ***  **
Gaac_spg1 CCAAAGAATATGTAACTCTGCCGGAAGTCGGAGACAGGGCTTGTGTATTGGTCTGGCCAGCTACGCCAAAGCCTGCGCCGGTGTTGGTGTTGTAATTGGTGACTGGAGGAGAATCACGGGCTGCGGTAAGTTTAGTTCTGAAAAATAAAC  5676
Gaac_spg2 CCAAAGAATATGTAACTCTG-----AGTCAGAAAGAGGCCATGTGTATTGGTCTTGCCAGCTACGCCAAAACCTGCGC------TGGTGTTGTAATTGGAGACTGGAGGAGATTCACGGGATGTGGTAAGTTTAGTTCTGAAAAGTAAAT  5330
Gaac_spg3 CCAAAGAATATGTAACTCTGCCAGGAGTCAGAAAGAGGCCTTGTGTATTGGTCTTGCCAGCTACGCCAAAGCCTGCGCCGGTGTTGGTGTTGTAATTGGTGACTGGAAGAAAAACATGGGCTGTGGTAAGTTTAGTTCTAAAATGTAA--  5757
Gaac_spg4 TCAAAGAATATGTAACTCTGGCAGGAGTCAGAAAGAGGCCTTGTGTATTGGTCTTGCCAGCTACGCCAAAGCCTGCGCCGGTGTTGGTGTTGTAATTGGTGACTGGAAGAAAAACATGGGCTGTGGTAAGTTTAGTTCTGAAAAGTAA--  5457
Gaac_spg5 CCAAAGAATATGTAACTCTGCCGGAAGTCGGAGACAGGGCTTGTGTATTGGTCTGGCCAGCTACGCCAAAGCCTGCGCCGGTGTTGGTGTTGTAATTGGTGACTGGAGGAGAATCACGGGCTGCGGTAAGTTTAGTTCTGAAAAATAAAC  5812
Gaac_spg6 CCAAAGAATATGTAACTCTGCCGGAAGTCGGAGACAGGGCTTTGTATTGGTCTGGCCAGCTACGCCAAAGCCTGCGCCGGTGTTGGTGTTGTAATTAGTGACTGGAGGAGAATCACAGGCTGTGGTAAGTTTAGTTCTGAAAAATAAAC  8146
Gaac_spg7 CCAAAGAATATGTAACACTGGCAGAAGTCGGAGAGAGGCCTTGTGTATTGGTCTGGCCAGCTATGCCAAAGCCTGCGACGGTCTAGGTGTTGTTATTGGTGACTGGAGGAGAAACACAGGCTGCAGTGAGTTTACTTCTGAAAAATATTC  8257
          .....12610.....12620.....12630.....12640.....12650.....12660.....12670.....12680.....12690.....12700.....12710.....12720.....12730.....12740.....12750

Gaac_spg1 AACGTATTCTGTGAACATC--CACACTATTTATTTTCTAGAGTTTACTGAAAAGAACCAATGGAGGTACGCTATGTTCAACA--------------AACTAAATCAACTGAATCAAATTAGTTCAGTTGCTTTTATGTGATTATGAGATG  5810
Gaac_spg2 AACGTATTCTGTGAATATCCTCACACTTTTTATTTTCTAGAGTTTACTGAAAAGAACCAATGGATGTACAGTATGTTCAACACAGTATGTTCAACAAACTAAATGAACTGAATCAAATTAGTTCCTTTGACTTTAATGAGATTGATTTTG  5480
Gaac_spg3 ------------------------------------------------------------------------------------------------------------------------------------------------------  5757
Gaac_spg4 ------------------------------------------------------------------------------------------------------------------------------------------------------  5457
Gaac_spg5 AACGTATTCTGTGAACATC--CACACAATTTATTTTCTAGAGTTTACTGAAAAGAACCAATGGAGGTACGCTATGTTCAACA--------------AACTAAATCAACTGAATCAAATTAGTT-----GCTTTTATGAGATTATGAGATG  5941
Gaac_spg6 AACATATTCTGTGAACATC--CACACACTTTATATTCTAGAGTTTACTGAAAAGAACCAATGGAGGTACGCTATGTTCAACA--------------AACTAAATCAACTGAATCAAATTAGTT-----GCTTTTATGAGATTATGAGATG  8275
Gaac_spg7 -------TATGTG--CATC--CTCCCACTTTATTTTCTAGAGTTCACTGAAAAGGACGAATGTAGGTACGGTATGTTCAACAAACTAAAACAAATCAGTTCCATTGGTTTTATAAGATTGTTATCTTATCTTTTTGATGATAACACAAT  8396
          .....12760.....12770.....12780.....12790.....12800.....12810.....12820.....12830.....12840.....12850.....12860.....12870.....12880.....12890.....12900

Gaac_spg1 TTATTTTCTTTTGTGATGATAATAATACAATGATGATTAAAAACTTTTCGTGTTGCAGAATTTTATAAATGTAA---TTAAAGATTTATACAGAATATAGTTTGAAATAATGCTGTTGATAAAAA----CGGGAGTCTAAATTAATAAAA  5953
Gaac_spg2 TCA------TTTGTGATGATAATACGATGATGATGATGACAAACAATGATTAAAAGCCTTTCAGATTCATT------TTAAGGATTTATACAGTTTATAGTTTGAA-TCAACATGTTTAAAAGACAACAGGTTTGTCCT-----------  5606
Gaac_spg3 ------------------------------------------------------------------------------------------------------------------------------------------------------  5757
Gaac_spg4 ------------------------------------------------------------------------------------------------------------------------------------------------------  5457
Gaac_spg5 TTATTTTCTTATTT--TGATAATAATGCAATGATGATTAAAAGCTTTTTGGGTTGCAGCATTTTATAAAAGTAATTGTTAAGGATTTATACAGAATATAGTTTTAAACAATGCTGTTGAAAAAAAACAACGGGAGTCTAAATTAATAAAA  6089
Gaac_spg6 TTATTTTCTTATTT--TGATAATAATACAATGATGATTAAAAGCTTTTCGGGTTGCAGCATTTTATAAAAGTAATTGTTAAGGATTTATACAGAATATAGTTTTAAACAATGCTGTTGAAAAAAAACAACGGGAGTCTAAATTAATAAAA  8423
Gaac_spg7 GACGATTAAAAACATTTAAGATTCCAAATGTTTACACGAGTACATCTTAAGTATTTATACTGATTATAGTTGAATCAACAACTGTTTGTTGTTGAGTGTAAAGTAAGTCAGTGTCACATTTTAGTAGATGTAGTACACATATGTCAAACC  8546
          .....12910.....12920.....12930.....12940.....12950.....12960.....12970.....12980.....12990.....13000.....13010.....13020.....13030.....13040.....13050

Gaac_spg1 TGTCCCATTTTAAATAGAATAAATGCATCATTGGTACATTTAATGT----------------------------------------------------------------------------------------------------------  5999
Gaac_spg2 -----------------------------ATTGGTACATTTAATGT----------------------------------------------------------------------------------------------------------  5623
Gaac_spg3 ------------------------------------------------------------------------------------------------------------------------------------------------------  5757
Gaac_spg4 ------------------------------------------------------------------------------------------------------------------------------------------------------  5457
Gaac_spg5 TGCCCCATTTTAA-TAGAATAAATAAATTGTTGGTACATTTAATGT----------------------------------------------------------------------------------------------------------  6134
Gaac_spg6 TGCCCCATTTTAA-TAGAATACATAAATCGTTGGTACATTTAATGT----------------------------------------------------------------------------------------------------------  8468
Gaac_spg7 CGGGGACGACTTTGCTATTGTGAGAATTACATAACGGTCCTCACGCATACATGGTCGCTTCTGCTTCTTTTATATCGATTCGTCCCTCCACCTCATGATTTTAATGGTCTTTGGTCTTTTAATAGTCTTTAATGGTTTGATCGAGGTGAG  8696
          .....13060.....13070.....13080.....13090.....13100.....13110.....13120.....13130.....13140.....13150.....13160.....13170.....13180.....13190.....13200

                                                                                                    17
Gaac_spg1 ----------------------------------------------------------------------------TGTGTCTCAGATCTGAAATGCCAGAAGAACCAAGAATTCTCCTACAGCATGCATATCTGCAACCGCACATGCAA  6073
Gaac_spg2 ----------------------------------------------------------------------------TGTGTCTCAGATTTGAAATGCCAGAAGAACCAAGAATGCACCAT--------------------GCACCTGCAC  5677
Gaac_spg3 ------------------------------------------------------------------------------------------------------------------------------------------------------  5757
Gaac_spg4 ------------------------------------------------------------------------------------------------------------------------------------------------------  5457
Gaac_spg5 ----------------------------------------------------------------------------TGTGTCTCAGATCTGAAATGCCAGAAGAACCAAGAATTCTCCTACAGCATGCATATCTGCAACCGCACATGCAC  6208
Gaac_spg6 ----------------------------------------------------------------------------TGTGTCTCAGATCTGAAATGCACTAAGAACCAAGAATTCTCCTACAGCATGCATACCTGCAACCGCACATGCAC  8542
Gaac_spg7 CAGTATCTGGCTTGAACCTACAATCAGTTTGACACCCCTGATGTAGTATAAATAAATCATTGGTGCATTTACTTACTGTGTCTCAGATCTGAAATGCCAGAAGAACCAAGAATTCTCCTACAACATGCATACCTGCAACCGCACATGCAC  8846
          .....13210.....13220.....13230.....13240.....13250.....13260.....13270.....13280.....13290.....13300.....13310.....13320.....13330.....13340.....13350

Gaac_spg1 TTCTCTGACCGGCCATGACATCCGCTGTGATATGAACGACGATGCTGTGGAGGGCTGTGGCTGTCCGGAGGGAACTCACCTGAACCAAGGACAGACCTGTTGCCCAAAGGAAGAGTGTGGTTGTATTTACTACGGTGGTATTGCAGCCCC  6223
Gaac_spg2 TTCTCTGTCCGGCCATGACATCTGCTGTGACATGAACGACAATCCTGTGGAGGGTTGTGGCTATCCGGAGGGAACTCACCTGAACCAAGGACAAATATGTTGCCCAAAGGAAGAGTGTGGTTGTATTTACTACGGTAGTATTGCAGACCC  5827
Gaac_spg3 ------------------------------------------------------------------------------------------------------------------------------------------------------  5757
Gaac_spg4 ------------------------------------------------------------------------------------------------------------------------------------------------------  5457
Gaac_spg5 TTCTCTTTCTGGCCATGACATCCGCTGTGACATGAACGACGATCCTGTGGAGGGCTGTGGCTGTCCGGAGGGAACTCACCTGAACCAAGGACAGACCTGTTGCCCAAAGGAAGAGTGTGGTTGTATTTACTACGGTGGTATTGCAGCCCC  6358
Gaac_spg6 TTCTCTTTCTGGCCATGACATCCGCTGTAATATGAACGACGATCCTGTGGAGGGCTGTGGCTGTCCGGAGGGAACTCACCTGAACCAAGGACAGACCTGTTGCCCAAAGGAAGAGTGTGGTTGTATTTACTACGGTGGTATTGCAGCCC-  8691
Gaac_spg7 TTCTCTGTCCGGCCATGACATTCGCTGTGATATGGACGACGATCCTGTGGAGGGCTGTGGCTGTCCGGAGGGAACTTACCTGAACCAAGGACAGACCTGTTCCCCAAAAAAGGAGTGTGCTTGTACTTACTACGGTGGGATTGCAGACCC  8996
          .....13360.....13370.....13380.....13390.....13400.....13410.....13420.....13430.....13440.....13450.....13460.....13470.....13480.....13490.....13500

Gaac_spg1 GGGGCCTGTTGTTATCGCTGGACAAAAGTGGTGAGTTGATTATCTGAGTATTGACAGCTTGTCTATAATAATACGACTGGTAATGGTTGAGGCTATTACCCATTGTCTTCCATAACCCCGTCTACCCAATGCTGTTATAGTGCGCTCGAT  6373
Gaac_spg2 TGGGTCTGTTGTTATCGCCGAACAAAAG------------------------------------------------------------------------------------------------------------------------  5855
Gaac_spg3 ------------------------------------------------------------------------------------------------------------------------------------------------------  5757
Gaac_spg4 ------------------------------------------------------------------------------------------------------------------------------------------------------  5457
Gaac_spg5 GGGGCCTGTTGTTATCGCCGGACAAAAGTGGTGGGTTGAATATCTGAGTATTGACAGCTTTTCCATAATAATACGAC--GTGATGGTTGAGGCTATTACCCGGTGTCTTCCTTAACCCTTTCTACCCAATGCTTTTATAGTGCACTCGTG  6506
Gaac_spg6 -----TGTTGTTATCGCCGGACAAAAGTGGTGAGTTGATTATCTGAGTATTGACAGCTTGTCTATAATAATACGACTGGTGATGGTTGAGGCTATTACCCGGTGTCTTCCTTAACCCTGTCTACCCAATGCTTTTATAGTGCACTCGTG  8835
Gaac_spg7 GGGGCCTGTTGTTATCTCAAGACAAAAGTGGTAAGGATCTTCTGAGTATAT--ACAGCCAGTCTATAATAATAAGACTGCTAATGCTTGAGGCTATTACCCGCTGTCTTCCTTAACCCTGTCTATCCAATGCAGTAATAGGGCACTCAA-  9143
          .....13510.....13520.....13530.....13540.....13550.....13560.....13570.....13580.....13590.....13600.....13610.....13620.....13630.....13640.....13650
```

```
                                                                                            18
Gaac_spg1 TGTGGTGTCTCCACCTTTGATGTACACATACTGAAGCAAAATTATGTTATATCTCAAAGCAAATATTGTGTCTGTTTCAGCGACTGCAAGAATGGGGTACTGAACTGCTTGCCGAATTGTGGTGAATATATATTCTTCCATCACAATCAT  6523
Gaac_spg2 ------------------------------------------------------------------------------------------------------------------------------------------------------  5855
Gaac_spg3 ------------------------------------------------------------------------------------------------------------------------------------------------------  5757
Gaac_spg4 ------------------------------------------------------------------------------------------------------------------------------------------------------  5457
Gaac_spg5 TGTGGTGTCTCCATCTTTGATGTTAAGATACT--AGCAAATATGTGTTATGTCTCAAAGCAAATATTGTGTCTGTTTTAGCAACTGCAAGAATGGGATACTGAGCTGTTTGCCAAATTGTGGTGAATATATATTCTTCCATCGCAATCAT  6654
Gaac_spg6 TGTGGTGTCTCCATCTTTGATGTTAAGATACT--AGCAAATATCTGTTATTTGTCAAAGCAAATATTGTGTCTGTTTTAGCAACTGCAAGAATGGGATACTGAGCTGTTTGTCAAATTGTGGTGAATATATATTCTTCCATCGCAATCAT  8983
Gaac_spg7 -------TCTCCACCTTTGATGTACACATACTGTCGCAAAATTGTGTTATATCTCAACACAAA---TGTGTTTATTTCAGCAACTGCGAGAATGGGGTACTGAGCTGTTTACCGGATTGTGGTGAATATAAATTCTTCTATCATAATTAT  9283
          .....13660.....13670.....13680.....13690.....13700.....13710.....13720.....13730.....13740.....13750.....13760.....13770.....13780.....13790.....13800

                                                                                                                                    19
Gaac_spg1 ATTAACATTGTACTCGTTCTTTGCTATCATAGTTTATATCATTATTTAAAATTCAAAAATGTGAAGAAAAGGAAATTTAAATTAG--GCATTTACCTTTCTGAAATATATATCACACAGATTGCAGAAATGGGAAGGTGTGTGTCAGTTG  6671
Gaac_spg2 ------------------------------------------------------------------------------------------------------------------------------------------------------  5855
Gaac_spg3 ------------------------------------------------------------------------------------------------------------------------------------------------------  5757
Gaac_spg4 ------------------------------------------------------------------------------------------------------------------------------------------------------  5457
Gaac_spg5 ATCAACATCATACTCGTTCTTTGTTTACCTTAGTTGA---ATTATTTCAAGTTCAAAAATTTAATTGAAAACAAATTCAAATTTG--ACATTTACCTGTCTGAAATATATATCACACAGATTGCAGAAATGGGAAGGTGTGTGTTAGTTG  6799
Gaac_spg6 ATCGACATCATACTCGTTCTTTGTTTACCTTAGTTCC---ATTATTTCAAGTTCAAAATTTTTAATGAAAACAAATTCAAATTTG--ACATTTACCTGTCTGAAATATATATCACACAGATTGCAGAAATGGGAAGGTGTGTGTTAGTTG  9128
Gaac_spg7 CTCATCATCAAACTTGTCCATTGTTATCTTAATTCCATTATTTAAACTTCAATAATCTTTAACAAAATGCAATTCAAACTTGGCAATGCATTTACTTGTCTCAAATATAATTCACACAGATTGCAAAAATGGGAAGGTGTGTGTCAGTTG  9433
          .....13810.....13820.....13830.....13840.....13850.....13860.....13870.....13880.....13890.....13900.....13910.....13920.....13930.....13940.....13950

Gaac_spg1 CTCTGAGGGCCAACATAAGAGGGTTCAGAAGACCTGTGACTATATTAGCAAACCAAAGGTAAGTCGACTGATTATTTGATTGTA------------------------------------------------------------------  6755
Gaac_spg2 ------------------------------------------------------------------------------------------------------------------------------------------------------  5855
Gaac_spg3 ------------------------------------------------------------------------------------------------------------------------------------------------------  5757
Gaac_spg4 ------------------------------------------------------------------------------------------------------------------------------------------------------  5457
Gaac_spg5 CTCTGAGGGCGAAAATAAAAGTGTTCGGAACACCTGTGACTATATCAGCAAACCAAAGGTAAGTCGCCTGGTTATGTGATTGTC------------------------------------------------------------------  6883
Gaac_spg6 CTCTGAGGGCGAAAATAAAAGTGTTCGGAACACCTGTGACTATATCAGCAAACCAAAGGTAAGTCGACTGATTATGTGATTGTCTCTGTTAGGGTTTCGGAAAGCTCTCAAGGGAGGCCTCACTCATTTTGTTCTCGTCCCGGCCGGAAA  9278
Gaac_spg7 CTCTGAGGGCCCAGTCAACATAGTTAAGAAGACCTGTGACCATATCGGGAAACCACTGGTAAGTCACCTGATTTTTTTGTTA--------------------------------------------------------------------  9515
          .....13960.....13970.....13980.....13990.....14000.....14010.....14020.....14030.....14040.....14050.....14060.....14070.....14080.....14090.....14100

Gaac_spg1 ------------------------------------------------------------------------------------------------------------------------------------------------------  6755
Gaac_spg2 ------------------------------------------------------------------------------------------------------------------------------------------------------  5855
Gaac_spg3 ------------------------------------------------------------------------------------------------------------------------------------------------------  5757
Gaac_spg4 ------------------------------------------------------------------------------------------------------------------------------------------------------  5457
Gaac_spg5 ------------------------------------------------------------------------------------------------------------------------------------------------------  6883
Gaac_spg6 CGAGGACACGAATGAGTCAATTTGTTTAAATTCAAAAATCAAAAGTTATTTAATAACTAAACAACGTTATAAGGAATGCAATTACGAAGTGAAATACTAAGCGAACAGTGGAATATTTCGCGAAATAGAGAATCCTCTGAGCAAGTCTAA  9428
Gaac_spg7 ------------------------------------------------------------------------------------------------------------------------------------------------------  9515
          .....14110.....14120.....14130.....14140.....14150.....14160.....14170.....14180.....14190.....14200.....14210.....14220.....14230.....14240.....14250

Gaac_spg1 ------------------------------------------------------------------------------------------------------------------------------------------------------  6755
Gaac_spg2 ------------------------------------------------------------------------------------------------------------------------------------------------------  5855
Gaac_spg3 ------------------------------------------------------------------------------------------------------------------------------------------------------  5757
Gaac_spg4 ------------------------------------------------------------------------------------------------------------------------------------------------------  5457
Gaac_spg5 ------------------------------------------------------------------------------------------------------------------------------------------------------  6883
Gaac_spg6 AGTGACTTTTCAAGGCGGCTGCAGGTCAGATTCTCCAGTCTTTTTCCCCGCCTTGGTCCTTTGAAAACTCTTGAGGTGTGTCTTCCTTGGTGCATTTGAATGTCATTGGCTTCTTCTCCAAAGGGCCACCTCCTGGACTGTCCCCTTCAC  9578
Gaac_spg7 ------------------------------------------------------------------------------------------------------------------------------------------------------  9515
          .....14260.....14270.....14280.....14290.....14300.....14310.....14320.....14330.....14340.....14350.....14360.....14370.....14380.....14390.....14400

Gaac_spg1 ------------------------------------------------------------------------------------------------------------------------------------------------------  6755
Gaac_spg2 ------------------------------------------------------------------------------------------------------------------------------------------------------  5855
Gaac_spg3 ------------------------------------------------------------------------------------------------------------------------------------------------------  5757
Gaac_spg4 ------------------------------------------------------------------------------------------------------------------------------------------------------  5457
Gaac_spg5 ------------------------------------------------------------------------------------------------------------------------------------------------------  6883
Gaac_spg6 GTCGAGGTGTGAAGCTGCAGCTGTAACCTGAAACCTCACTGTACTATCTATCCCTCACATTCCAATGCACTCAATGTAGATACAGTTGGCTTTATGCCCCAATGAGTGAATTTAACAAAGCATACATCGTTTAAGTCTTCATCTTATAAC  9728
Gaac_spg7 ------------------------------------------------------------------------------------------------------------------------------------------------------  9515
          .....14410.....14420.....14430.....14440.....14450.....14460.....14470.....14480.....14490.....14500.....14510.....14520.....14530.....14540.....14550

Gaac_spg1 ------------------------------------------------------------------------------------------------------------------------------------------------------  6755
Gaac_spg2 ------------------------------------------------------------------------------------------------------------------------------------------------------  5855
Gaac_spg3 ------------------------------------------------------------------------------------------------------------------------------------------------------  5757
Gaac_spg4 ------------------------------------------------------------------------------------------------------------------------------------------------------  5457
Gaac_spg5 ------------------------------------------------------------------------------------------------------------------------------------------------------  6883
Gaac_spg6 TAGTACACGTTCATTACAACAACCCATCATTAATGATCACCGTTCATCACACTTCATCATAAGATCTAAGACAGTTCATGTGGTCCAATTCTCAAGACATTTGCCTCAGTCGGCTGCCTTACATTAAGTTGTTCATTTATTACCTGACAG  9878
Gaac_spg7 ------------------------------------------------------------------------------------------------------------------------------------------------------  9515
          .....14560.....14570.....14580.....14590.....14600.....14610.....14620.....14630.....14640.....14650.....14660.....14670.....14680.....14690.....14700

Gaac_spg1 ------------------------------------------------------------------------------------------------------------------------------------------------------  6755
Gaac_spg2 ------------------------------------------------------------------------------------------------------------------------------------------------------  5855
Gaac_spg3 ------------------------------------------------------------------------------------------------------------------------------------------------------  5757
Gaac_spg4 ------------------------------------------------------------------------------------------------------------------------------------------------------  5457
Gaac_spg5 ------------------------------------------------------------------------------------------------------------------------------------------------------  6883
Gaac_spg6 GAGAAAAAACTCCCAAAAAACCCTCTTTGGGGATAAAATTGGATGAAATCCATAAAGAACGGCAATTGGCATCCGTCCCCAGGTTTTAACATCACATTGTGACAGAATGTTAATGTGTACAGTGTTTATATGATTAACATGACTATGAAT 10028
Gaac_spg7 ------------------------------------------------------------------------------------------------------------------------------------------------------  9515
          .....14710.....14720.....14730.....14740.....14750.....14760.....14770.....14780.....14790.....14800.....14810.....14820.....14830.....14840.....14850

Gaac_spg1 ------------------------------------------------------------------------------------------------------------------------------------------------------  6755
Gaac_spg2 ------------------------------------------------------------------------------------------------------------------------------------------------------  5855
Gaac_spg3 ------------------------------------------------------------------------------------------------------------------------------------------------------  5757
Gaac_spg4 ------------------------------------------------------------------------------------------------------------------------------------------------------  5457
Gaac_spg5 ------------------------------------------------------------------------------------------------------------------------------------------------------  6883
Gaac_spg6 TGTGTTTGATTGAAATTGCATTCACTTCATCTAACATGTTAATGTAATAACTAATATCTAAGAACACACAAACTATAATTCTAACACTAAACATTATACACGTACTATAATTTCCTGACTAGGGGTGCACTTCTGGCTTAAATCCCTAAC 10178
Gaac_spg7 ------------------------------------------------------------------------------------------------------------------------------------------------------  9515
          .....14860.....14870.....14880.....14890.....14900.....14910.....14920.....14930.....14940.....14950.....14960.....14970.....14980.....14990.....15000

Gaac_spg1 ---TCCATGGCTTGTTGGTCTATTTATTTGTATCCATTCTTCAATTGAAAGCAGTCACTACTTACCATTAAACAAAATTGGATAATGTTGCTATTTAATATCACGAGGGAGACTTGTTTTTACAGGATCAGGAATCAGGAATCAGGAAAC  6902
Gaac_spg2 ------------------------------------------------------------------------------------------------------------------------------------------------------  5855
Gaac_spg3 ------------------------------------------------------------------------------------------------------------------------------------------------------  5757
Gaac_spg4 ------------------------------------------------------------------------------------------------------------------------------------------------------  5457
Gaac_spg5 ---TCCATGGCTTGTTTGTCTATTTATTTGCATTCATTCATCAATTGAAAGCAGTCACTACTTACCATTAAAAATATAAGGATAATCTTGCTAATTAATATCATGAGGGAGACTTGTTTTTAAGGTTTTTTTGA----------------  7014
Gaac_spg6 ATCTCCATGGCTTGTTTGTCTATTTATTTGCATTCATTCATCAATTGAAAGCAGTCACTACTTACCATTAAAAATATAAGGATAATGTTGCTAATTAATATCATGAGGGAGACTTGTTTTTAAGGTTTTTTTAAGGTGATTCTTTTCTTG 10328
Gaac_spg7 --------TGCTTGTTTGTCTGTTTATTTGTATACATTCTTTAATTGAAATCAGTAACTAGTTGCAATTTAAAACATATAATATAGCTATTTAATATCATTAGGGATATGTTTTAAGGTAATGTGATGCTTAACTTAATTGAGTGTTACT  9657
          .....15010.....15020.....15030.....15040.....15050.....15060.....15070.....15080.....15090.....15100.....15110.....15120.....15130.....15140.....15150

Gaac_spg1 ATTTATTTGCCAAAATATGCTAAACATACATACAAGGAATTTGTCTTGGCGGTTAGTGCGCGACAGTAGACAGACAAGACAACAGTGCACTAGTAATAAAATAAAGTAAAATGAAATGCTCATGCAATGGGTTAGTAGAAAAAGGCTATG  7052
Gaac_spg2 ------------------------------------------------------------------------------------------------------------------------------------------------------  5855
Gaac_spg3 ------------------------------------------------------------------------------------------------------------------------------------------------------  5757
Gaac_spg4 ------------------------------------------------------------------------------------------------------------------------------------------------------  5457
Gaac_spg5 ------------------------------------------------------------------------------------------------------------------------------------------------------  7014
Gaac_spg6 CCCATTCT---------------------------------------------------------------------------------------------------------------------------------------------- 10336
Gaac_spg7 GATATTTTACCTTCATGTCAGTCAATTTTATCCTTAATATCAAAAAGAAAGGTTTATTTTATTTTTATTTCATTTTAATTTTAT------------------------------------------------------------------  9741
          .....15160.....15170.....15180.....15190.....15200.....15210.....15220.....15230.....15240.....15250.....15260.....15270.....15280.....15290.....15300

Gaac_spg1 GGTTAGTAGAAAACAAGGGAATAAAGTTAAAAATTTTAAATATTAAAAAAGTTAAAAAGAAAAATACTAAGCATAAGGTGCACTAACAAACAAGTAACAGACAAGTAACAAAGTGACGAGTGACGACAAAGTGATGAATTGCAGTTAAAG  7202
Gaac_spg2 ------------------------------------------------------------------------------------------------------------------------------------------------------  5855
Gaac_spg3 ------------------------------------------------------------------------------------------------------------------------------------------------------  5757
Gaac_spg4 ------------------------------------------------------------------------------------------------------------------------------------------------------  5457
Gaac_spg5 ------------------------------------------------------------------------------------------------------------------------------------------------------  7014
Gaac_spg6 ------------------------------------------------------------------------------------------------------------------------------------------------------ 10336
Gaac_spg7 ------------------------------------------------------------------------------------------------------------------------------------------------------  9741
          .....15310.....15320.....15330.....15340.....15350.....15360.....15370.....15380.....15390.....15400.....15410.....15420.....15430.....15440.....15450

Gaac_spg1 TGGCATGTGCAGTGTGAAGGGGAGTGACCGATGGAGTGTTATAGTCAGTCAGTGGGGGACCGGGCTCTGTTGATGAGCCCGAGTGCCGACGGGAAGAAACGGTTCGTGTGGCGGGAGGTCTTAGTCCTGATGGACCTCAGCCTCCTGCCA  7352
Gaac_spg2 ------------------------------------------------------------------------------------------------------------------------------------------------------  5855
Gaac_spg3 ------------------------------------------------------------------------------------------------------------------------------------------------------  5757
Gaac_spg4 ------------------------------------------------------------------------------------------------------------------------------------------------------  5457
Gaac_spg5 -----------------------------------------------------------------------------------------------------------------GGTGATTCTTTTCTTGCCCATTCTTAACTTCATGTTG  7051
Gaac_spg6 ------------------------------------------------------------------------------------------------------------------------------------------------------ 10336
Gaac_spg7 ------------------------------------------------------------------------------------------------------------------------------------------------------  9741
          .....15460.....15470.....15480.....15490.....15500.....15510.....15520.....15530.....15540.....15550.....15560.....15570.....15580.....15590.....15600
```

```
Gaac_spg1 GATGGAAGGGGCAACGGTTTTATAACTTCATGTTGTATGTGCATGTCTTCATG--------------GACATGTACATTGTATCCTCAATGTTAACCCTATTCCAAATACAGTTTATTATTGTCGTCTATAAAACACTTCCTAATAACAA  7488
Gaac_spg2 ------------------------------------------------------------------------------------------------------------------------------------------------------  5855
Gaac_spg3 ------------------------------------------------------------------------------------------------------------------------------------------------------  5757
Gaac_spg4 ------------------------------------------------------------------------------------------------------------------------------------------------------  5457
Gaac_spg5 A------------------------------------ATGTGCATGTCTTCATGTGCATGTCTTCATGTACATGTTCATTGTATCCTCAATGTTAACCCAAATTCAAATACAGTTTTTATTGTCGTCTATAAAACACTTTCTAATAACAA  7166
Gaac_spg6 -----------------------TAACTTCATGTTGAATGTGCATGTCTTCATGTGCATGTCTTCATGTACATGTTCATTGTATCCTCAAGGTTAACCCAAATTCAAACAAAGTTTTTTTTGTCGTCTATAAAACACTTTCTAATAACAA 10464
Gaac_spg7 ------------------------------------------------------------------------------------------------------------------------------------------------------  9741
          .....15610.....15620.....15630.....15640.....15650.....15660.....15670.....15680.....15690.....15700.....15710.....15720.....15730.....15740.....15750

                                   20
Gaac_spg1 AATATCTTCATTTGCTGTGATTGGCAGGGTACCAGGGAGAACTGTAAGAGTGGCTGTTACTGTCCAGATCACCAGTATGAAGATCACCATGGGAACTGTGTTTCACTCGATGATTGCACCTGTGTGTTCAGTGGCAAAGCATTCAAAGCT  7638
Gaac_spg2 ------------------------------------------------------------------------------------------------------------------------------------------------------  5855
Gaac_spg3 ------------------------------------------------------------------------------------------------------------------------------------------------------  5757
Gaac_spg4 ------------------------------------------------------------------------------------------------------------------------------------------------------  5457
Gaac_spg5 AATTTCTTCATTTGCTGTGATTGGCAGGGTACCAGGGAGACCTGTAAAAGTGGCTGTTACTGTCCAGATCACCAGTATGAAGATCACCATGGGAACTGTGTTTCACTCGATGATTGCACCTGTGTGTTCAGTGGCAAAGCATTCAAAGCA  7316
Gaac_spg6 AATATCTTCATTTGCTGTGATTGGCAGGGTACCAGGGAGACCTGTAAAAGTGTCCGTTACTGTCCAGATCACCAGTATGAAGATCACCATGGGAGCTGTGTTTCACTCGATGAATGCACTTGTGTGTTCAGTGGCAAAGCATTCAAAGCA 10614
Gaac_spg7 ----------------GTGATTGACAGGATACCAGTGAGACCTGTGAGAGTGGCTGTTACTGTCCAGATCACCAGTATGAGGATCACTTTGGGAACTGTGTTTCACTCGATGAATGCACCTGTGTTTTCAGTGGCAAAGCATTCAGAACA  9875
          .....15760.....15770.....15780.....15790.....15800.....15810.....15820.....15830.....15840.....15850.....15860.....15870.....15880.....15890.....15900

                                                                                                                                          21
Gaac_spg1 GGACAGCAAGTTACCAGCAACTGTAAAACATGGTAACTCCCA-------CTTATTTGATCAAAGGGTCCTATCCCGCCAAATGCTGATGAATCTGTGAATAAAAGGATGACATCACTCTATGTTACAGTACCTGTTATCGTGGTCAGTGG  7781
Gaac_spg2 ------------------------------------------------------------------------------------------------------------------------------------------------------  5855
Gaac_spg3 ------------------------------------------------------------------------------------------------------------------------------------------------------  5757
Gaac_spg4 ------------------------------------------------------------------------------------------------------------------------------------------------------  5457
Gaac_spg5 GGACAGCAAGTTACCAGCAACTGTAAAACATGGTAACTCCCA-------CTTATTTGATCAAAGGGTCCTATCCCGCCAAACGCTGATGAATCTGTGAATAAAAGGATGACATCACTCTATGTTACAGTACCTGTTATCGTGGTCAGTGG  7459
Gaac_spg6 GGACAGCAAGTTACCAGCAACTGTAAAACATGGTAACTCCCA-------CTTATTTGATCAAAGGGTCCTATCCCGCCAAACGCTGATGAATCTGTGAATAAAAGGATGACATCACTCTATGTTACAGTACCTGTTATCGTGGTCAGTGG 10757
Gaac_spg7 GGACAGCAAGTCAACAGCAGGTGTAAAACATGGTAACCCCTAACGCCTACTTATTAGTTTAAAGGGTCTTATCTCGCCAAACGCTGATGGAACTGTGAATAAAAGGTGTCCATCCCTCTATGTTACAGTACCTGTTATCGTGGTATGTGG 10025
          .....15910.....15920.....15930.....15940.....15950.....15960.....15970.....15980.....15990.....16000.....16010.....16020.....16030.....16040.....16050

Gaac_spg1 CACTGCATTGAGAAGCCTTGCCCGGGACAGTGCCAAGTCTACGGAAATGGACACTACCAGACCTTTGACTCCAAATGGTTCCGCTTTTCTGGACAATGTCTGTACACACTTGTGCAGGTAAGACCAAACAAGAAAGTAGGAATGAAATCC  7931
Gaac_spg2 ------------------------------------------------------------------------------------------------------------------------------------------------------  5855
Gaac_spg3 ------------------------------------------------------------------------------------------------------------------------------------------------------  5757
Gaac_spg4 ------------------------------------------------------------------------------------------------------------------------------------------------------  5457
Gaac_spg5 CACTGCATTGAGAAGCCTTGCCCGGGACAGTGCCAAGTCTACGGAAATGGACACTACCAGACCTTTGACTCCAAATGGTTCCGCTTTTCTGGACAATGTCTGTATACACTTGTGCAGGTAAGACCAAACAAGAAAGTAGGAATGAAATCC  7609
Gaac_spg6 CACTGCATTGAGAAGCCTTGCCCGGGACAGTGCCAAGTCTACGGAAATGGACACTACCAGACCTTTGACTCCAAATGGTTCCGCTTTTCTGGACAATGTCTGTATACACTTGTGCAGGTAAGACCAAACAAGAAAGTAGGAATGAAATCC 10907
Gaac_spg7 CAATGCACTGAGAAGCCTTGTCCGGGACAATGTCAAGTTTACGGAGATGGACACTACCAGACCTTTGACTCCAAATGGTACCGCTTTTCTGGACAATGTCTGTACACACTTGTGCAGGTAAGACCAAACTAAAAAGTAGGAATGAAATCC 10175
          .....16060.....16070.....16080.....16090.....16100.....16110.....16120.....16130.....16140.....16150.....16160.....16170.....16180.....16190.....16200

                                                                                                                          22
Gaac_spg1 ACTCACGCTCATATGTGTTTGCAATGCATCGCCAAATATTGAATGGCTTCTTTATTTATAGACAGAATTTGCCAAATTT-TATTAACTTTTAAACTCTTCTTGCTTCAGGATTCCTGTGACATGAGAAGAGGCACCTTCTCTATCAGAGT  8080
Gaac_spg2 ------------------------------------------------------------------------------------------------------------------------------------------------------  5855
Gaac_spg3 ------------------------------------------------------------------------------------------------------------------------------------------------------  5757
Gaac_spg4 ------------------------------------------------------------------------------------------------------------------------------------------------------  5457
Gaac_spg5 ACTCACGCTCATATGTGTTTGCAATGCATCGCCAAATACTGAAGGGCTTCTTTATTTATAGACAGAATTTGCCAAATTTGTATTAACTTTTAAACTCTTCTTGCTTCAGGATTCCTGTGACATGAGAAGAGGCACCTTCTCTATCAGAGT  7759
Gaac_spg6 ACTCACGCTCATATGTGTTTGCAATACATCGCCAAATGCTGAAGGGCTTCTTTATTTATAAACAGAATTTGCCAAATTTGTATTAACTTTTAAACTCTTCTTGCTTCAGGATTCCTGTGACATGAGAAGAGGCACCTTC----TCAGAGT 11053
Gaac_spg7 ACTCACGCTCATACGTGTTTCTAATCCTTT---AAATGACACAGCATATCATCCAAATACTAAAGGG-------------------CTTTT-AACTCTTCTTGTTTCAGGATTCCTGTGGAAATAAAAAAGGCACCTTCTCTGTCAGAGT 10302
          .....16210.....16220.....16230.....16240.....16250.....16260.....16270.....16280.....16290.....16300.....16310.....16320.....16330.....16340.....16350

Gaac_spg1 GGAGAGTGTCCCCTGCTGTGAGGAGGTGCTCACCTGCTCTCGCAACATCATCCTTGACCTGAAGGTGGATTTTTTAGTTTCTATTTTACAGATACATCAATCAATAAAAGTCTAGTTACTTGTTTATCAACGCATTTATTCTCGTAACCC  8230
Gaac_spg2 ------------------------------------------------------------------------------------------------------------------------------------------------------  5855
Gaac_spg3 ------------------------------------------------------------------------------------------------------------------------------------------------------  5757
Gaac_spg4 ------------------------------------------------------------------------------------------------------------------------------------------------------  5457
Gaac_spg5 GGAAAGTGTCCCCTGCTGTGAGGAGGTGCTCACCTGCTCTCGCAACATCATCCTTGACCTGAAGGTGGATTTTTTAGTTTCTATTTTACAGATACATCAATCAATAAAAGTCTAGTTACTTGTTTATCAACCCATTTATTCTCGTAACCC  7909
Gaac_spg6 GGCGAGTGTCCCCTGCTGTGAGGAGGTGCTCACCTGCTCTCGCAACATCATCCTTGACCTGAAGGTGGATTTTTTAGTTTCTATTTTACAGATACATCAATCAATAAAAGTCTAGTTACTTGTTTATCAACGCATTTATTCTCGTAACCC 11203
Gaac_spg7 GGAGAGTGTCCCCTGCAATCAGAATACGCTCACGTGCTCCCGCAATATCATCCTCAAACTGCAGGTGCATTTTTTTTTACTCTGACTGAATTTTACAGAGGAGTTTCAGTCACTAAAAGTTTAGTTGTTAATCAACACGTACATTGAATT 10452
          .....16360.....16370.....16380.....16390.....16400.....16410.....16420.....16430.....16440.....16450.....16460.....16470.....16480.....16490.....16500

                                             23
Gaac_spg1 AAGGTCTACT----CTTTTTTTTTT-ACCTAAAAAGGGCCAAGTCACCCTGACGCTGAGAGACATGCAGGTGATCAGACGCCTCCATGAAGGCTGGAATGGACAGGATGATTCACTTTACTCAATACACACTTTGGGACTTTACATTGTA  8375
Gaac_spg2 ------------------------------------------------------------------------------------------------------------------------------------------------------  5855
Gaac_spg3 ------------------------------------------------------------------------------------------------------------------------------------------------------  5757
Gaac_spg4 ------------------------------------------------------------------------------------------------------------------------------------------------------  5457
Gaac_spg5 AAGGTCTACT----CTTTTTTTTTTTACCTAAAAAGGGCCAAGTCACCCTGACGCTGAGAGACATGCAGGTGATCAGACGCCTCCATGAAGGCTGGAATGGGCAGGATGATTCACTTTACTCAATACACACTTTGGGACTTTACATTGTA  8055
Gaac_spg6 AAGGTCTACT----CTTTTTTTTT--ACCTAAAAAGGGCCAAGTCACCCTGACGCTGAGAGACATGCAGGTGACCAGATGCCTCCATGAAGGCTGGACTGGGCAGGATGATTCACTTTACTCAATACACACTTTGGGACTTTACATTGTA 11347
Gaac_spg7 TTACAACCCGAGTTCTACTTTTTTTAACCTAAAAAGGGCAAAGTCACCCTGACGCTGAGCGACATGCAGGTGACCAGACGCCTCCATAAAGGCTGGACTGGGCACGATGATTCACTTTACTCAATACAGACTTCGGGACTTTACATTGTA 10602
          .....16510.....16520.....16530.....16540.....16550.....16560.....16570.....16580.....16590.....16600.....16610.....16620.....16630.....16640.....16650

Gaac_spg1 ATCTCAGTGCCAAGCAAAGGGATAACTCTCATCTGGGACAAACACACCCGGATCACCGTAGAGCTGGCGGGTTCGTGGAAGGTGAGTCAAGAGGATTTAAAACCTAGCCATTTATTAACATCAGTTTGATGCTTTTGATTAGACAAAAT  8525
Gaac_spg2 ------------------------------------------------------------------------------------------------------------------------------------------------------  5855
Gaac_spg3 ------------------------------------------------------------------------------------------------------------------------------------------------------  5757
Gaac_spg4 ------------------------------------------------------------------------------------------------------------------------------------------------------  5457
Gaac_spg5 ATCTCAGTGCCAAGCAAAGGGATAACTCTCATCTGGGACAAACACACCCGGATCACCGTAGAGCTGGCGGGTTCGTGGAAGGTGAGTGAAGAGGATTTTAAAACCTAGCCATTTATTTACATCAGTTTGATGCTTTTGATTAGACAAAAT  8205
Gaac_spg6 ATCTCAGTGCCAAGCAAAGGGATAACTCTCATCTGGGACAAACACACCCGGATCACCGTAGAGCTGGCGGGTTTGTGGAAGGTGAGTGAAGAGGATTTTAAAACCTAGCCATTCATTTACATCAGTTTGATGCATTTGATTAGACAAAAT 11497
Gaac_spg7 ATCTCAGTTCCAAGCAAAGGGATAACTCTCATCTGGGACAAACACACCTGGATCACCGTAGAGCTGGCGGTTTCGTGGAAGGTGAGTGAGTAAAATCTAGTAATGTACATACTATACATCACTCTTCACTTTGATCCTCAGAAAAAAATT 10752
          .....16660.....16670.....16680.....16690.....16700.....16710.....16720.....16730.....16740.....16750.....16760.....16770.....16780.....16790.....16800

Gaac_spg1 AATAAAATGGGGGGGGGGGGGGGGACACTTCTAAAGACTGAATTGAATTTGAAACCAACATTTTATACGGTTACTTAATGTCTTCAACTTTGCAGTTATTTAGCACAAATCAACTCAAATTTATATCAAACTGAATATGAATCGGATTT  8675
Gaac_spg2 ------------------------------------------------------------------------------------------------------------------------------------------------------  5855
Gaac_spg3 ------------------------------------------------------------------------------------------------------------------------------------------------------  5757
Gaac_spg4 ------------------------------------------------------------------------------------------------------------------------------------------------------  5457
Gaac_spg5 AATAAAATGGTGGGGGGGGGGG---CACTTCTAAAGACTGAATTGATCTTGAAACCAACAATTTATACGGTTACTCAATGTCTTCTT--CTGCAGTTATTTAGCACAAATCAACTTAAATTTATATCAAACTGAATATGAATCGGAATT  8350
Gaac_spg6 AATAAAATGGGTGGGGG--------ACACTTCTAAAGACTGAATTGAATTTTAAACCAACATTTTATACGGTTACTCAATGTCTTCTT--CTGCGGTTATTTAGCACAAATCAACTCAAATTTATATCTAACTGACTATGAATCGGAATT 11637
Gaac_spg7 AAAAATGTTGGAGACGCTTCCCAACTTAGGCCTAAAT------TTGAATTTGAAACAAACATTTTATACGCTTACTTTCATCAACTTTAATTCTCAACTTTGCATTGATTTATTTCATCCAACAAGGAGAAATAAACTAAATTTCATATC 10896
          .....16810.....16820.....16830.....16840.....16850.....16860.....16870.....16880.....16890.....16900.....16910.....16920.....16930.....16940.....16950

Gaac_spg1 ACGGACTTTGGAAAAATCAAGACTCCAGCAGTTACTTAAATTTTG-TTTGGTACTTTTTCAGAACTTC----------------------------------------------------------------------CACATTTACTTT  8754
Gaac_spg2 ------------------------------------------------------------------------------------------------------------------------------------------------------  5855
Gaac_spg3 ------------------------------------------------------------------------------------------------------------------------------------------------------  5757
Gaac_spg4 ------------------------------------------------------------------------------------------------------------------------------------------------------  5457
Gaac_spg5 ACGGACTTTGGAAAAATCAAGACTCAAGCAGTTACTTACATTTTGCTTTGGTACTTTTTCAGAACTTC----------------------------------------------------------------------CACATTTACTTT  8430
Gaac_spg6 ACGGA-------AGAATCAAGACTCCAGCAGTTACTTAAATTTTG-TTTGGTACTTTTTCAGAACTTC----------------------------------------------------------------------CACATTTACTTT 11709
Gaac_spg7 GAAACAACCATCAATTTTAATGACCAACTTTCATAAAAAAAGAGTACGTCTCATTGTTGTATTCATCATAACTAATAGCTACATCCTCCAGCAGTTACCTACATTTGTTAGTATTTTTTAACTTTTGTATATATTTCACATTGACTTT 11046
          .....16960.....16970.....16980.....16990.....17000.....17010.....17020.....17030.....17040.....17050.....17060.....17070.....17080.....17090.....17100

                                24
Gaac_spg1 CTAACCATCTCTCCTACAGAACCGAGTGTGTGGCCTCTGTGGGAATTTTGACTCCAATGAGATGAATGACCTAAGTATAAGTGGTTCATCAGGTAGATAGGTGTTTTCTATGTTTGTTGTTTCATAAAAGATTTTAAATTTTTTGGTGG  8904
Gaac_spg2 ------------------------------------------------------------------------------------------------------------------------------------------------------  5855
Gaac_spg3 ------------------------------------------------------------------------------------------------------------------------------------------------------  5757
Gaac_spg4 ------------------------------------------------------------------------------------------------------------------------------------------------------  5457
Gaac_spg5 CTAACCATCTCTCCTACAGAACCGAGTGTGTGGCCTCTGTGGGAATTTTGACTCCAATGAGATGAATGACCTAAGTATAAGTGGTTCATCAGGTAGATAGGTGTTTTCTATGTTTGTTGTTTCATAAAAGATTTTACATTTTTTGGTGG  8580
Gaac_spg6 CTAACCATCTCTCCTACAGAACCGAGTGTGTGGCCTCTGTGGGAATTTTGACTCCAATGAGATGAATGACCTAAGTATAAGTGGTTCATCAGGTAGA--GGTGTTTTCTATGTTTGTTGTTTCATAAAGGACTTTACATTTTTTGGTGG 11857
Gaac_spg7 CTAACCATCTCTCCTACAGAACCGAGTGTGTGGCCTATGTGGGAATTTTGACTACAATGAGATGAATGACCTAATTATACGTAGCTCATCAGGTAAATAGATGTTCTCTTTGTTTGATAAAGGACTTTTACTTTTGTCATGGTGTTCAA- 11195
          .....17110.....17120.....17130.....17140.....17150.....17160.....17170.....17180.....17190.....17200.....17210.....17220.....17230.....17240.....17250

                                       25
Gaac_spg1 TCGACTTAACACCCAACAAATATGTTTTCTAGTGTCCAGTCCAATGGCATTTGGCAACTGCTGGAAAGTCCAAACACCTCCTTGCTCTGATGTGACCACTGATATATTTCCATGTGAACGCAACTCCTACTGCGCAGCCTGGGCCCAGCA  9054
Gaac_spg2 ------------------------------------------------------------------------------------------------------------------------------------------------------  5855
Gaac_spg3 ------------------------------------------------------------------------------------------------------------------------------------------------------  5757
Gaac_spg4 ------------------------------------------------------------------------------------------------------------------------------------------------------  5457
Gaac_spg5 TCGACTTAACACCCA-CAAATATGTTTTCTAGTGTCCAGTCCAATGGCATTTGGCAACTGCTGGAAAGTCCAAACACCTCCTTGCTCTGATGTGACCACTGATATATTTCCATGTGAACGCAACTCCTACTGCGCAGCCTGGGCCCAGCA  8729
Gaac_spg6 TCGACTTAACACCCAACAAATATGTTTTCTAGTGTCCAGTCCAATGGCATTTGGCAACTGCTGGAAAGTCCAAACACCTCCTTGCTCTGATGTGACCACTGATATATTTCCATGTGAACGCAACTCCTACTGCGCAGCCTGGGCCCAGCA 12007
Gaac_spg7 ---AC-TAACACCAAAAAAATATGTTTTCTAGTGTTCGGTCGCCTGGCATTTTGTAACAGCTGGAAAGTCCATACACATTCTTGCTCCGATGTGACCACTGAGATATATGCAGGTCAACGCAACTCCTACTGCACAGCCTGGGCCCAGCA 11341
          .....17260.....17270.....17280.....17290.....17300.....17310.....17320.....17330.....17340.....17350.....17360.....17370.....17380.....17390.....17400

Gaac_spg1 GCGCTGTTTGATCCTTACAGGAGAAACATTCAGTGAATGCCACTTAAAAGTATGCATGTTATAGATTTAGCACAAACAACGTCAGGTGTTAGACTGCTAAATATTTTACAAATTTCCATATCCGAATATGTCGTAATATGAAACCATGCA  9204
Gaac_spg2 ------------------------------------------------------------------------------------------------------------------------------------------------------  5855
Gaac_spg3 ------------------------------------------------------------------------------------------------------------------------------------------------------  5757
Gaac_spg4 ------------------------------------------------------------------------------------------------------------------------------------------------------  5457
Gaac_spg5 GCGCTGTTTGATCCTTACAGGAGAAACATTCAGTGAATGCCACTTAAAAGTATGCATGTTATAGATTTAGCACAAACAACGTCAGGTGTTCGACTGCTAGATATTTTATGAATTTCCATATCCCAATCTGTCCTTACATGAAACCA----  8875
Gaac_spg6 GCGCTGTTTGATCCTTACAGGAGAAACATTCAGTGAATGCCACTTAAAAGTATGCATGTTATAGATTTAGCACAAACAACGTCAGGTGTTAGACTGCTAAATATTTTATAAATTTCCATATCCCAATCTGTCCTAACATGAAACCA---- 12153
Gaac_spg7 GCGCTGTTCGGTCATTTCAGGAGAAACATTCAGTGAATGCCACTTAAAGGTGTGCATGTTACATATTTAGCACAGTACAAAGAAAAAGGTCAGGTGTTAGACTGCCATATATTTGGGATTTCCATATCCAAATCTGTTATATTGCAATAT 11491
          .....17410.....17420.....17430.....17440.....17450.....17460.....17470.....17480.....17490.....17500.....17510.....17520.....17530.....17540.....17550
```

26

```
Gaac_spg1 ACTGTTTACAAAAAAATATTCTCACAGATAGCCCTAAAATGTGTAGATACAAACTCCTTCCTGGTTGTTTTCCAATCCCCAGGTGGATCCAGATCCCTACTACGATGCCTGTGTGCAGGAGTCTTGCTCCTGTGAGTCAGACGGGAAATT  9354
Gaac_spg2 ------------------------------------------------------------------------------------------------------------------------------------------------------  5855
Gaac_spg3 ------------------------------------------------------------------------------------------------------------------------------------------------------  5757
Gaac_spg4 ------------------------------------------------------------------------------------------------------------------------------------------------------  5457
Gaac_spg5 --TGTTTACAAAAAAATATTCTCACAGATAGCCCTAAAATGTGTAGATACAAACTCCTTCCTGGTTGTTTTCCAATCCCCAGGTGGATCCAGATCCCTACTACGATGCCTGTGTGCAGGAGTCTTGCTCCTGTGAGTCAGACGGGAAGTT  9023
Gaac_spg6 --TATTTACAAAAAAAGATTCTCACAAATAGCCCTAAAATGTGTAGATACAAACTCCTTCCTGGTTGTTTTCCAATCCCCAGGAGGATCCAGATCCCTACTACGATGCCTGTGTGCAGGAGTCTTGCTCCTGTGAGTCAGACGGGAAGTT 12301
Gaac_spg7 GAAATCATATATCTATTTAAAGACCCTAACAATGAGCCCTTAAATGTTCTA-------TCCTGGTTGTTTTCTAATCCCCAGGTTGATCCAGATCCCTACTACGTTGCCTGTGTCCAGGAGTCTTGCTCCTGTGAGTTAGATGTACAATT 11634
          .....17560.....17570.....17580.....17590.....17600.....17610.....17620.....17630.....17640.....17650.....17660.....17670.....17680.....17690.....17700

Gaac_spg1 CCTCGGCTTCTGCACAGCTGTGGCTGCCTACGCACAGGCCTGCAGCGAGCAGCATGTGTGTGTGAATTGGAGGACACCTGATATGTGTCGTAAGTAAGGACCTTTTT---TCCCTCACTAACGACACATGAACTTTACTCTAAAACAGGG  9501
Gaac_spg2 ------------------------------------------------------------------------------------------------------------------------------------------------------  5855
Gaac_spg3 ------------------------------------------------------------------------------------------------------------------------------------------------------  5757
Gaac_spg4 ------------------------------------------------------------------------------------------------------------------------------------------------------  5457
Gaac_spg5 CCTCGGCTTCTGCACAGCTGTGGCTGCCTACGCACAGGCCTGCAGCGAGCAGCATGTGTGTGTGAATTGGAGGACACCTGATATGTGTCGTAAGTTAGGACCTTTTT---TCCCTCACTAACGACACATGAACTTTACTCTAAAAGAC--  9168
Gaac_spg6 CCTCGGCTTCTGCACAGCTGTGGCTGCCTACGCACAGGCCTGCAGCGAGCAGCATGTGTGTGTGAATTGGAGGACACCTGATATGTGTCGTAAG                                                        12395
Gaac_spg7 CCTTGGCTTCTGCACAGCTGTGGCTGCCTATGCACAGGCCTGCAGCGAGCAGCATGTTTGTGTGAATTGGAGGACACCTGATTTGTGTCGTAAGTTAAGACCTTTTCTTTCCTGTGAGTAACAAAACGTGACCTTAACTCCAAAAGAC-- 11782
          .....17710.....17720.....17730.....17740.....17750.....17760.....17770.....17780.....17790.....17800.....17810.....17820.....17830.....17840.....17850

Gaac_spg1 GTGTCAAACTCATTTCAGGTTCGGGCCAGATACTGCCCACTTCGATCGAACGTAGGCTAGACCATTAAAACGAGCTATAGTTCCAATTCATTGTCTTGCTAGACTGGGTTAATGTGACACATTTAGTATTACAGACAATGTAATAGTCTG  9651
Gaac_spg2 ------------------------------------------------------------------------------------------------------------------------------------------------------  5855
Gaac_spg3 ------------------------------------------------------------------------------------------------------------------------------------------------------  5757
Gaac_spg4 ------------------------------------------------------------------------------------------------------------------------------------------------------  5457
Gaac_spg5 -------ACTTTGTTTTAACATGTTTTAATAACTTGTAATTTAAAGCCA------GTAA----AATAATGTAAGCACCAGCC---------AGTTCTG--AAAATCAATTC----------CTTA-TAT--------------AGTTTG  9264
Gaac_spg6                                                                                                                                                        12395
Gaac_spg7 -------ACT-------GACATTTTTTAATAACTTCTAACTTAAAGCCAT-ACAGATAACATGGATAACACAAGCACCGGTC---------TGTTCTA--AAATTCAGTTC----------CTTAATGG--------------GATGTC 11881
          .....17860.....17870.....17880.....17890.....17900.....17910.....17920.....17930.....17940.....17950.....17960.....17970.....17980.....17990.....18000

Gaac_spg1 TTGGAATGTCACAAGCTCCGTAAACATTATATAATGTTTTATTGATTTAGTCATGTTTCTTA--GTCATTATGCAGAGACCTGACGCAGTGTAAGACATAAATAAAGTAATCAACTTTTTTAATCAACAATTAATCTGTTGAAAATTGTC  9799
Gaac_spg2 ------------------------------------------------------------------------------------------------------------------------------------------------------  5855
Gaac_spg3 ------------------------------------------------------------------------------------------------------------------------------------------------------  5757
Gaac_spg4 ------------------------------------------------------------------------------------------------------------------------------------------------------  5457
Gaac_spg5 TTAGAATCTTACAAACTTCATAAACATTATATAATGTTTTATCAATTTAGTCGTGTTTCTTA--GTGATTATGCAGAGACCTGACAAAGTGTAAGACTTACGTAAAGTAATAAACTTTTGTAAT--------------------------  9386
Gaac_spg6                                                                                                                                                        12395
Gaac_spg7 CTAATAGTTCCCAAACATAATTTAGATCTTTTAAAAAAAAA-CAATTTAATTCTATTGTGAAATGTGGGGAGATCAGTTTTTAAGACAGAAGTAAACTAAAGAGGAAAAGTAATTTGTTTTATTGAAAAAATATGTCATAGAGT------ 12024
          .....18010.....18020.....18030.....18040.....18050.....18060.....18070.....18080.....18090.....18100.....18110.....18120.....18130.....18140.....18150

Gaac_spg1 ATAATTATTACCTTACCTGTTTAGAAAATATAAACTATAACGGGGTATTTCTGTAAAAGTTTAACTTGTGATCATTCAAACTATAGCGGTGTACTGTGACTACTACAACAAGCATGGACAGTCTATCTGGCACTATGAAGCCTGTGGTCA  9949
Gaac_spg2 ------------------------------------------------------------------------------------------------------------------------------------------------------  5855
Gaac_spg3 ------------------------------------------------------------------------------------------------------------------------------------------------------  5757
Gaac_spg4 ------------------------------------------------------------------------------------------------------------------------------------------------------  5457
Gaac_spg5 ------------------------------------------------------------------------------------------------------------------------------------------------------  9386
Gaac_spg6                                                                                                                                                        12395
Gaac_spg7 ------------------------------------------------------------------------------------------------------------------------------------------------------ 12024
          .....18160.....18170.....18180.....18190.....18200.....18210.....18220.....18230.....18240.....18250.....18260.....18270.....18280.....18290.....18300

Gaac_spg1 GATGCTGACCTGTGGCAAAGAAAACTATATCTCTCACAAGCTGGAAGGTAACCAAACATTCCCATTTCTATACAAACAGCCACATTTAATTCTTATTCATGGTAAGATGCAAACTGAAAACAATGTATTTCTACAAACAACCATCACAAT 10099
Gaac_spg2 ------------------------------------------------------------------------------------------------------------------------------------------------------  5855
Gaac_spg3 ------------------------------------------------------------------------------------------------------------------------------------------------------  5757
Gaac_spg4 ------------------------------------------------------------------------------------------------------------------------------------------------------  5457
Gaac_spg5 ------------------------------------------------------------------------------------------------------------------------------------------------------  9386
Gaac_spg6                                                                                                                                                        12395
Gaac_spg7 ------------------------------------------------------------------------------------------------------------------------------------------------------ 12024
          .....18310.....18320.....18330.....18340.....18350.....18360.....18370.....18380.....18390.....18400.....18410.....18420.....18430.....18440.....18450

Gaac_spg1 CTAGATCAAATCAAAGGTTTTCTTCTATTCAAAGCATAAGAAAACAATAGCACTCATATTCAGACACATTTTGTTTATTTTTGGAAAAGGCTGCTACCCAAGATGTACAGAGAAGAAGCCATACTATGATGAAAACACTGGTGGATGCAC 10249
Gaac_spg2 ------------------------------------------------------------------------------------------------------------------------------------------------------  5855
Gaac_spg3 ------------------------------------------------------------------------------------------------------------------------------------------------------  5757
Gaac_spg4 ------------------------------------------------------------------------------------------------------------------------------------------------------  5457
Gaac_spg5 ------------------------------------------------------------------------------------------------------------------------------------------------------  9386
Gaac_spg6                                                                                                                                                        12395
Gaac_spg7 ------------------------------------------------------------------------------------------------------------------------------------------------------ 12024
          .....18460.....18470.....18480.....18490.....18500.....18510.....18520.....18530.....18540.....18550.....18560.....18570.....18580.....18590.....18600

Gaac_spg1 CAGTTTAGGAAACTGCACCTGTTACCTTAATGGCACTATCGTTCAACCTCAGGCAGTGGTAGTAATACAGAATGTTTTATGGTAAGTTGGGCAATTTGTTTTTTGTCATTTCTGTTCATTTTAAAAATACTAACATTATACGTATGGGGC 10399
Gaac_spg2 ------------------------------------------------------------------------------------------------------------------------------------------------------  5855
Gaac_spg3 ------------------------------------------------------------------------------------------------------------------------------------------------------  5757
Gaac_spg4 ------------------------------------------------------------------------------------------------------------------------------------------------------  5457
Gaac_spg5 ------------------------------------------------------------------------------------------------------------------------------------------------------  9386
Gaac_spg6                                                                                                                                                        12395
Gaac_spg7 ------------------------------------------------------------------------------------------------------------------------------------------------------ 12024
          .....18610.....18620.....18630.....18640.....18650.....18660.....18670.....18680.....18690.....18700.....18710.....18720.....18730.....18740.....18750

Gaac_spg1 CATTTTTAATATTGATATATATGTATATATACTGTTTCAAAATACATAAATGTACAGTATCTCCATACTAATAATTTCATTACTCATTATGTTGGCAACTGAATGTTTCTCACTTCCCAACTTTATTATTTTCAGCCGCTGTGAAAATGG 10549
Gaac_spg2 ------------------------------------------------------------------------------------------------------------------------------------------------------  5855
Gaac_spg3 ------------------------------------------------------------------------------------------------------------------------------------------------------  5757
Gaac_spg4 ------------------------------------------------------------------------------------------------------------------------------------------------------  5457
Gaac_spg5 ------------------------------------------------------------------------------------------------------------------------------------------------------  9386
Gaac_spg6                                                                                                                                                        12395
Gaac_spg7 ------------------------------------------------------------------------------------------------------------------------------------------------------ 12024
          .....18760.....18770.....18780.....18790.....18800.....18810.....18820.....18830.....18840.....18850.....18860.....18870.....18880.....18890.....18900

Gaac_spg1 AGCCATGAACTGCGGTAATTATCAATTATATTGATAAACCACTGCCTTTACTATTTCATGTGTGTAATATCGGTTACACGGAACTATGGAATAGTTGTGTAACTTTGGTTTTTAAATGGTTTAGAGTTTGTACCAAAACAGAGAATGTTT 10699
Gaac_spg2 ------------------------------------------------------------------------------------------------------------------------------------------------------  5855
Gaac_spg3 ------------------------------------------------------------------------------------------------------------------------------------------------------  5757
Gaac_spg4 ------------------------------------------------------------------------------------------------------------------------------------------------------  5457
Gaac_spg5 ------------------------------------------------------------------------------------------------------------------------------------------------------  9386
Gaac_spg6                                                                                                                                                        12395
Gaac_spg7 ------------------------------------------------------------------------------------------------------------------------------------------------------ 12024
          .....18910.....18920.....18930.....18940.....18950.....18960.....18970.....18980.....18990.....19000.....19010.....19020.....19030.....19040.....19050

Gaac_spg1 TCAAAACACCCAACCTCAATTCCGCCTCTTCTTTGACAGACCCTTCATCGTCCACCACTCCAACATCCAGCACTCACACTGCTTCTGATTCAACAACCAGGGATGTGACTCGAACACACCATACTCCCACCGCTTCTGATTCAACAACCA 10849
Gaac_spg2 ------------------------------------------------------------------------------------------------------------------------------------------------------  5855
Gaac_spg3 ------------------------------------------------------------------------------------------------------------------------------------------------------  5757
Gaac_spg4 ------------------------------------------------------------------------------------------------------------------------------------------------------  5457
Gaac_spg5 ------------------------------------------------------------------------------------------------------------------------------------------------------  9386
Gaac_spg6                                                                                                                                                        12395
Gaac_spg7 ------------------------------------------------------------------------------------------------------------------------------------------------------ 12024
          .....19060.....19070.....19080.....19090.....19100.....19110.....19120.....19130.....19140.....19150.....19160.....19170.....19180.....19190.....19200

Gaac_spg1 GGGATGTGACTCGAACACACCATACTCCCACCGCTTCTGATTCAACAACCAGGGATGTGACTCGAACACACCATACTCACACCGCTTCTGATTCAACAACCAGGGATGTGACTCGAACACACCATACTCACACCGCTTCTGATTCAACAA 10999
Gaac_spg2 ------------------------------------------------------------------------------------------------------------------------------------------------------  5855
Gaac_spg3 ------------------------------------------------------------------------------------------------------------------------------------------------------  5757
Gaac_spg4 ------------------------------------------------------------------------------------------------------------------------------------------------------  5457
Gaac_spg5 ------------------------------------------------------------------------------------------------------------------------------------------------------  9386
Gaac_spg6                                                                                                                                                        12395
Gaac_spg7 ------------------------------------------------------------------------------------------------------------------------------------------------------ 12024
          .....19210.....19220.....19230.....19240.....19250.....19260.....19270.....19280.....19290.....19300.....19310.....19320.....19330.....19340.....19350

Gaac_spg1 CCACCCATATGACTCGAACGCACCACACTCACACCTATACTGTTTCCACAACCAAGGGTGCGTGCCATCAACTTGTGCTGCACATTACACCATTGTTTCAGTTATTCTTGCGACCAGTTCCTTAAATCGCCAAAGTTATAATTGTGTCAT 11149
Gaac_spg2 ------------------------------------------------------------------------------------------------------------------------------------------------------  5855
Gaac_spg3 ------------------------------------------------------------------------------------------------------------------------------------------------------  5757
Gaac_spg4 ------------------------------------------------------------------------------------------------------------------------------------------------------  5457
Gaac_spg5 ------------------------------------------------------------------------------------------------------------------------------------------------------  9386
Gaac_spg6                                                                                                                                                        12395
Gaac_spg7 ------------------------------------------------------------------------------------------------------------------------------------------------------ 12024
          .....19360.....19370.....19380.....19390.....19400.....19410.....19420.....19430.....19440.....19450.....19460.....19470.....19480.....19490.....19500

Gaac_spg1 TTCTTTATTGAGTTTCTTCACCCGTCTTGTGCTCATCAAATCACCAGATTGCCAGTGTTTTGACCCAAAGAATTATAAAATCTGGCACTGCGGTGAGACGTG 11251
Gaac_spg2 ------------------------------------------------------------------------------------------------------  5855
Gaac_spg3 ------------------------------------------------------------------------------------------------------  5757
Gaac_spg4 ------------------------------------------------------------------------------------------------------  5457
Gaac_spg5 ------------------------------------------------------------------------------------------------------  9386
Gaac_spg6                                                                                                        12395
Gaac_spg7 ------------------------------------------------------------------------------------------------------ 12024
          .....19510.....19520.....19530.....19540.....19550.....19560.....19570.....19580.....19590.....19600..
```
