# Supplementary material for: Extensive lineage-specific gene duplication and evolution of the spiggin multi-gene family in stickleback
Source: BMC Evol Biol. 2007 Nov 4;7:209. doi: 10.1186/1471-2148-7-209 (PMC2180178; doi:10.1186/1471-2148-7-209)
Supplement: Additional file 2 — "Sequences of spiggin genes in threespine and ninespine sticklebacks and their homologs in other fishes". Sequences of the spiggin multi-gene family in threespine stickleback (Gaac_spg1, 3, 4, 5, and 7; spg1-4), ninespine stickleback (Pungitius_spgα-γ), and their homologs in torafugu (Takifugu_spiggin_homolog), spotted green pufferfish (Tetraodon_spiggin_homolog), medaka (Oryzias_spiggin_homolog), and zebrafish (Danio_spiggin_homolog) that were used in the phylogenetic analyses. The resulting data matrix, excluding ambiguous regions, was aligned before phylogenetic analyses were performed. [file 1471-2148-7-209-S2.pdf]

>Gaac_spg1
MTTQRWILAFCLSLAVFGTVELFKTKEIQTYTCRTFGSGIVQPFKGESYYVRSDCPFKLTSFNVNRGEYSVTIRRGHNGLLVQVEIIVN
KVTTLLQNGHILVQNNSVSLPYDHTYQHIFKYGIYTRLRSSLLPFTVTWHNVHGGINSLWVTLESELCTDMCGLCGKQNVAGHRDELIR
ESKLHDHRCKIRDPVLQKNHICRRFFLKTKNCLQDNNSHYHRLCKENICGFENSQSIFCPFFQEVASQCNQSRINRFWRRLTRCAKPRC
PGDLIYEKKGPAFIPSCSNPNPAPFYQELTETCACPNGKVLNNCEKGYRCIPKSSCSCEFAGKTYGNGEIRSSRCQSCTCDGGKWRCSE
NFCHRRCVIEGQFVTTFDGKQYVLPNKCLYVASKGPNWIIIIEFSQKKLHIRKVTVQLMEELFVFKKNKVLFDGQEIPEFHFSGHAQVY
WVSSMFVQVHTTIGINFQIQLSPEIHLFIDAPDTSNDKIKGLCGNSNSDTTDDFTTNSGIIENSAKPFAMSWSLLNCFGNIPTTCTNLE
NENYAHEKCAVLNQPTGIFAKCHPHIPTDYYYTACIQRICNSAGSRRQGLCIGLASYAKACAGVGVVIGDWRRITGCDLKCQKNQEFSY
SMHICNRTCNSLTGHDIRCDMNDDAVEGCGCPEGTHLNQGQTCCPKEECGCIYYGGIAAPGPVVIAGQKCDCKNGVLNCLPNCDCRNGK
VCVSCSEGQHKRVQKTCDYISKPKGTRENCKSGCYCPDHQYEDHHGNCVSLDDCTCVFSGKAFKAGQQVTSNCKTCTCYRGQWHCIEKP
CPGQCQVYGNGHYQTFDSKWFRFSGQCLYTLVQDSCDMRRGTFSIRVESVPCCEEVLTCSRNIILDLKGQVTLTLRDMQVIRRLHEGWN
GQDDSLYSIHTLGLYIVISVPSKGITLIWDKHTRITVELAGSWKNRVCGLCGNFDSNEMNDLSISGSSVSSPMAFGNCWKVQTPPCSDV
TTDIFPCERNSYCAAWAQQRCLILTGETFSECHLKVDPDPYYDACVQESCSCESDGKFLGFCTAVAAYAQACSEQHVCVNWRTPDMCRK
>Gaac_spg3
MTTQRRILAFSLSLAVFGTVELLKTKETQTYTCRTFGSGIVQPFQGESYYVRSDCPFTLTSFNVNQGEYSVTIRRGPNGLLVQVEIIVN
KVTTLLQDGNVVVKNKSVSDPCDQKYQQIFKYGIYTRLKSSLLPFSVTWHSVCGGINSLWVSHRDKLIRDSKLHDHKCKTSSPVLQKND
ICRQFFQEIKDCVRYNNSHYQQLCEENIFGFENSQSVFCPFFKEFASQCNQSRINRFWRDLTKCAEPRCPGDLIYREKGPAVIPSCSNP
KPPPFYQELTESCACPEGNVLNNGAKGYRCIPWSNCSCEFAGKSYRNGEIRRSKCHSCTCHGVVWRCSENFCRRRCVIEGPFVTTFDGK
QHVLPQKCSYVASKGPNWRIRIHFSRKGLYLRKVVVRFSEELFVFKENKVLWNGQEITKFHQFQENCNAKIYWVSSTFLQVHTTSGFFF
QIQLSPEIQLFIDAPDNSNYKIEGLCGNSNNNTTDDFITKSRIIENSAEPFALSWSLGNCQGNIHTNCTKREYENYAHEKCAVLNQPTG
VFAQGHPHIPTDYYYEACIQRICNSGRSQKEALCIGLASYAKACAGVGVVIGDWKKNMGCGKFSSKM
>Gaac_spg4
MQFDHWVSFASRVLKGSEISGKTNETQTYTCRTFGSGIVQPFKGESYYVRSDCQFILISFTVNGKEYSVTIRRGHNGLLVQVEIIINKV
TTLLQNGHVVVKNKKVTHPCNRKYQHIFKYGIYSRLRSSLLPFTVTWHNVHGGINSLWVTLESELNTTTSGLCGKQNIAGHRDKLIRDS
KLHDHKCKTSSPVLQKNDICRQFFQEIKDCVQYNISHYQRLCEENIFGFENNQSVFCPFFKEFASQCNQTRINRFWRRLTKCAEPRCPG
DLIYREKGPAVIPSCSNPKPLPFYQELTESCACPEGKVLNNGAKGYRCIPWSNCSCEFAGKSYRNGEIRRSKCHSCTCHGVVWRCSENF
CHRRCVIEVPFVTTFDGKKYVLPYKCAYLASKGPSWRIRIHFSRKGLYLRKVVVRLPGELIVFRKNKYAKIYWCLTSQVHTTPVIFQIQ
LSPEIQLFIDAPNQSKSDDKIEGLCGNSNRNTTDDFTTETGFFPNSVQLFALSWIMGNCQGNIPTTCTKRENENYAHEKCAVLNQPTGV
FAQGHPHIPTAYYYKACIQRICNSGRSQKEALCIGLASYAKACAGVGVVIGDWKKNMGCGKFSSEK
>Gaac_spg5
MTTQRWILAFCLSLAVFGTVELFKTKEIQTYTCRTFGSGIVQPFKGESYYVRSDCPFKLTSFNVNRGEYSVTIRRGHNGLLVQVEIIVN
KVTTLLQNGHILVQNNSVSLPYDHTYQHIFKYGIYTRLRSSLLPFTVTWHNVHGGINSLWVTLESELCTDMCGLCGKQNVAGHRDELIR
ESKLHDHRCKIRDPVLQKNHICRRFFLKTKNCLQDNNSHYHRLCKENICGFENSQSIFCPFFQEVASQCNQSRINRFWRRLTRCAKPRC
PGDLIYEKKGPAFIPSCSNPNPAPFYQELTETCACPKGKVLNNCEKGYRCIRKSSCSCEFAGKTYGNGEIRSSRCQSCTCDGGKWRCSE
NFCHRRCVIEGQFVTTFDGKQYVLPNKCLYVASKGPNWIIIIEFSQKKLHIRKVTVQLMEELFVFTKNKALFDGQEISEFHLSGHAQVY
WVSSMFIQVHTTIGINFQIQMSPEIQLFIDAPDTSNDKIKGLCGNSNSDTTDDFTTNSGIIENSAKPFAMSWSLLNCFGNIPTTCTNLE
NENYAYEKCAVLNQPTGIFAKCHPHIPTDYYYTACIQRICNSAGSRRQGLCIGLASYAKACAGVGVVIGDWRRITGCDLKCQKNQEFSY
SMHICNRTCTSLSGHDIRCDMNDDPVEGCGCPEGTHLNQGQTCCPKEECGCIYYGGIAAPGPVVIAGQKCNCKNGILSCLPNCDCRNGK
VCVSCSEGENKSVRNTCDYISKPKGTRETCKSGCYCPDHQYEDHHGNCVSLDDCTCVFSGKAFKAGQQVTSNCKTCTCYRGQWHCIEKP
CPGQCQVYGNGHYQTFDSKWFRFSGQCLYTLVQDSCDMRRGTFSIRVESVPCCEEVLTCSRNIILDLKGQVTLTLRDMQVIRRLHEGWN
GQDDSLYSIHTLGLYIVISVPSKGITLIWDKHTRITVELAGSWKNRVCGLCGNFDSNEMNDLSISGSSVSSPMAFGNCWKVQTPPCSDV
TTDIFPCERNSYCAAWAQQRCLILTGETFSECHLKVDPDPYYDACVQESCSCESDGKFLGFCTAVAAYAQACSEQHVCVNWRTPDMCRK
LGPFFPH
>Gaac_spg7
MTTQRWILVFIFSLADTCRTFGSGIVQPFKGESYYVRSDCPFTLTSFTVNRGEYSVTIRRGRNGLLVQVEIIINKVMTLLQNGHVVVQN
KSVSLPYDHTYQHIVKYGIYTKLRSSLLPFTVTWHNVDGGINSLWVTLESELSTDMCGLCGKQNLAGIRLIRDSQLHDLRCKTRDPVLQ
KNHVCSQFFRKTKSCLQDNNSHFHQLCRDNIFGFENSHEVFCPFFQEVASQCNQSRINRFWRRLTRCEKPSCPGDLIYEKKGPAFIPSC

SNPDPAPFYQELTETCVCPRDKVFNNHAIGHHCIPESSCSCEFAGKSYGNGEIRSSKCQSCTCHGGKWRCSENLCHRKCVIEGQFVTTF
DGKQYVLPRKCLYVASKGPNWMITIQFNQKGLSLRKVMVHLSKELFVFKNSKVVFEGREIMEFHQTDHAQVYWLSSMFVQVHTTCGIHF
QIQLSPEIQLFIDTPDKSNDKIKGLCGNSNSDTTDDFTANSGIIENSAQPFALSWSLGHCHGNIPSNCTNRENENYAHEKCAVLHQPTG
MFAKCHPHIPTDYYYTACIQRICNTGRSRREALCIGLASYAKACDGLGVVIGDWRRNTGCNLKCQKNQEFSYNMHTCNRTCTSLSGHDI
RCDMDDDPVEGCGCPEGTYLNQGQTCSPKKECACTYYGGIADPGPVVISRQKCNCENGVLSCLPDCDCKNGKVCVSCSEGPVNIVKKTC
DHIGKPLDTSETCESGCYCPDHQYEDHFGNCVSLDECTCVFSGKAFRTGQQVNSRCKTCTCYRGMWQCTEKPCPGQCQVYGDGHYQTFD
SKWYRFSGQCLYTLVQDSCGNKKGTFSVRVESVPCNQNTLTCSRNIILKLQGKVTLTLSDMQVTRRLHKGWTGHDDSLYSIQTSGLYIV
ISVPSKGITLIWDKHTWITVELAVSWKNRVCGLCGNFDYNEMNDLIIRSSSVFGRLAFCNSWKVHTHSCSDVTTEIYAGQRNSYCTAWA
QQRCSVISGETFSECHLKVDPDPYYVACVQESCSCELDVQFLGFCTAVAAYAQACSEQHVCVNWRTPDLCRKLRPFLSCE
>spg1
MTTQQWILAFCLSLAVFGTVELFKTKEILTYTCRTFGSGIVQPFKGESYYVRSDCPFKLTSFNVNRGEYSVTIRRGHNGLLVQVEIIIN
KVTTLLQNGNILVQNKSVSLPYDHTYQHIFKYGIYTRLRSSLLPFTVTWHNVHGGINSLWVTLESELCTDMCGLCGKQNVAGHRDELIR
DSKLHDHRCKIKDPVLRKNHICRQFFRKTKNCLQDNNSHYHRLCKENICGFENSQSIFCPFFQEIASQCNQSRINRFWRRLTRCAKPRC
PGDLIYEKKGPAFIPSCSNPNPAPFYQELTETCSCPEGKVLNNCEKGYRCIPKSSCSCEFAGKTYGNGEIRSSRCQSCTCHGGKWRCSE
NFCHRRCVIEGQFVTTFDGKQYVLPNKCLYVASKGPNWIIIIEFSQKKLYIRKVTVQLMEELFVFKNNKVLFDGQEIPEFHFSGHAQVY
WVSSMFVQVHTTIGINFQIQMSPEIHLFIDAPDTSSDKIKGLCGNSNSDTTDDFTTNSGIIENSAKPFAMSWSLLNCFGNIPTTCTNLE
NENYAHEKCAVLNQPTGIFAKCHPHIPTDYYYTACIQRICNSAGSRRQGLCIGLASYAKACAGVGVVIGDWRRITGCDLKCQKNQEFSY
SMHTCNRTCNSLTGHDIRCGMNDDAVEGCGCPEGTHLNQGQTCCPKEECGCIYYGGIAAPGPVVIAGQKCDCKNGILNCLPNCDCRNGK
VCVSCSEGQHKRVQKTCDYISKPKGTRENCKSGCYCPDHQYEDHHGNCVSLDDCTCVFSGKAFKAGQQVTSNCKTCTCYRGQWHCIEKP
CPGQCQVYGNGHYQTFDSKWFRFSGQCLYTLVQDSCDMRRGTFSIRVESVPCCEEVLTCSRNIILDLKGQVTLTLRDMQVTRRLHEGWT
GQDDSLYSIHTLGLYIVISVPSKGITLIWDKHTRITVELAGSWKNRVCGLCGNFDSNEMNDLSISGSSVSGPMAFGNCWKVQTPPCSDV
TTDIFPCERNSYCAAWAQQRCLILTGETFSECHLKVDPDPYYDACVQESCSCESDGKFLGFCTAVAAYAQAAASSMCV
>spg2
MTTQRWILAFCFSLATYTCRTFGSGIVQPFKGESYYVRSDCQFILIGFTVNGKEYSVTIRRGHNGLLVQVEIIINKVTTLLQNGHVVVK
SALFELRQKTGLGHRHQWRRQRLKVTHPCNRKYQHIFKYGIYNRLKSSLLPFSVTWHNVHGGINSLWVTLESELNTTTSGLCGKQNIAG
HRDELIRDSKLHDHKCNTSSPVLQKNDICRQFFQEIKDCVQYNNSHYQRLCEENIFGFENNQSVFCPFFKEFASQCNQSRINRFWRRLT
KCAKPRCPGDLIYRENGPAVIPSCSNPKPLPFYQELTETCACPEGKVLNNGAKGYRCIPWSNCSCEFAGKSYRNGEIRRSKCHSCTCHG
VVWRCSENFCHRRCVIEGPFVTTFDGKKYVLPYKCAYLASKGPSWRIRIHFSRKGLYLRKVVVRLPGELIVFRKNKVLWNWQEITQFHQ
FQETCNAKIYWVSSTFLQVHTTSGLFFQIQLSPEIQLFIDAPNQSKSDDKIEGLCGNSNRNTTDDFTTETGFFPNSVQLFALSWIMGNC
QGNIPTTCTKRENENYAHEKCAVLNQPTGIFAKCHPHIPTDYYYTACIPRICNTGRSRREALCIGLASYAKACDGLGVVIGDWRRNTGC
NLKCQKNQEFSYNMHTCNRTCTSLSGHDFRCDMDDDPVEGCGCPEGTYLNQGQTCSPKKECACTYYGGIADPGPVVISRQKCNCENGVL
SCLPDCDCKNGKVCVSCSEGPVNIVKKTCDHIGKPLDTSETCESGCYCPDHQYEDHFGNCVSLDECTCVFSGKAFRTGQQVNSRCKTCT
CYRGMWQCTEKPCPGQCQVYGDGHYQTFDSKWYRFSGQCLYTLVQDSCGNKKGTFSVRVESVPCNQNTLTCSRNIILKLQVHFFYAD
>spg3
MTTQRWILAFCFSLATYTCRTFGSGIVQPFKGESYYVRSDCQFILIGFTVNGKEYSVTIRRGHNGLLVQVEIIINKVTTLLQNGHVVVK
SALFELRQKTGLGHRHQWRRQRLKVTHPCNRKYQHIFKYGIYNRLKSSLLPFSVTWHNVHGGINSLWVTLESELNTTTSGLCGKQNIAG
HRDELIRDSKLHDHKCNTSSPVLQKNDICRQFFQEIKDCVQYNNSHYQRLCEENIFGFENNQSVFCPFFKEFASQCNQSRINRFWRRLT
KCAKPRCPGDLIYRENGPAVIPSCSNPKPLPFYQELTESCACPEGKVLNNGAKGYRCIPWSNCSCEFAGKSYRNGEIRRSKCHSCTCHG
VVWRCSENFCHRRCVIEGPFVTTFDGKKYVLPYKCAYLASKGPSWRIRIHFSRKGLYLRKVVVRLPGELIVFRKNKVLWNWQEITQFHQ
FQETCNAKIYWVSSTFLQVHTTSGLFFQIQLSPEIQLFIDAPNQSKSDDKIEGLCGNSNRNTTDDFTANSGIIENSAQPFALSWSLGHC
HGNIPSNCTNSENENYAHEKCAVLHQPTGMFAKCHPHIPTDYYYTACIQRICNTGRSRREALCVGLASYAKACDGLGVVIGDWRRNTGC
NLKCQKNQEFSYNMHTCNRTCTSLSGHDIRCDMDDDPVEGCGCPEGAYLNQGQTCSPKKECACTYYGGIADPGPVVISGQKCDCENGVL
SCLPDCDCKNGKVCVSCSEGPVNIVKKTCDRIGKPLDTSETCESGCYCPDHQYEDHFGNCVSLDECTCVFSGKAFRTGQQVNSRCKTCT
CYRGMWQCTEKPCPGQCQVYGDGHYQTFDSKWYRFSGQCLYTLVQDSCGNKKGTFSIRVKGEFVNCR
>spg4
MTTQRWILAFCLSLAVSGTVELFKTKEILTYTCRTFGSGIVQPFKGESYYVRSDCPFKLTSFNVNRGEYSVTIRRGHNGLLVQVEIIIN
KVTTLLQNGHILVQNKSVSLPYDHTYQHIFKYGIYTRLRSSLLPFTVTWHNVHGGINSLWVTLESELCTDMCGLCGKQNVAGHRDELIR

DSKLHDHRCKIRDPVLRKNHICRRFFRKTKNCLQDNNSHYHRLCKENICGFENSQSIFCPFFQEVASQCNQSRINRFWRRLTRCAKPRC
PGDLIYEKKGPAFIPSCSNPNPAPFYQELTETCACPEGKVLNNCEKGYRCIPKSSCSCEFAGKTYGNGEIRSSRCQSCTCDGGKWRCSE
NFCHRRCVIEGQFVTTFDGKQYVLPNKCLYVASKGPNWIIIIEFSQKKLYIRKVTVQLMEELFVFTKNKALFDGQEISEFHLSGHAQVY
WVSSMFIQVHTTIGINFQIQMSPEIQLFIDAPDTSNDKIKGLCGNSNSDTTDDFTANSGIIENSAQPFALSWSLGHCHGNIPTTCTNLE
NENYAYEKCAVLNQPTGIFAKCHPHIPTDYYYTACIQRICNSAGSRRQGLCIGLASYAKACAGVGVVIGDWRRITGCDLKCQKNQEFSY
SMHICNRTCTSLSGHDIRCNMNDDPVEGCGCPEGTHLNQGQTCCPKEECGCIYYGGIAAPGPVVIAGQKCDCKNGILNCLPNCDCRNGK
VCVSCSEGQHKRVQKTCDYISKPKGTRENCKSGCYCPDHQYEDHHGNCVSLDDCTCVFSGKAFKAGQQVTSNCKTCTCYRGQWHCIEKP
CPGQCQVYGNGHYQTFDSKWFRFSGQCLYTLVQDSCDMRRGTFSIRVESVPCCEEVLTCSRNIILDLKGQVTLTLRDMQVTRRLHEGWT
GQDDSLYSIHTLGLYIVISVPSKGITLIWDKHTRITVELAGSWKNRVCGLCGNFDSNEMNDLSISGSSVSGPMAFGNCWKVQTPPCSDV
TTDIFPCERNSYCAAWAQQRCLILTGETFSECHLKVDPDPYYDACVQESCSCESDGKFLGFCTAVAAYAQAAASSMCV
>Pungitius_spgα
LAFCFSLASVFGTVELFETKETQTYTCRTFGSGVVQPFKGETYYVRSDCPFTLTSFTVNREEYSVSIRRGHNGLLVQVEIIINKVMTLL
QNGQILEQNKSVSLPYDHTYQHIFKYGIYTRLRSTVLPFTVTWHNVHGGINSMWVTLESKLSADMSGLCGQQKVAAPRHELIRDSKLDD
HRCKTRDPVTQKNHMCGQFFRKTKNCLQDNNRHYHRLCKENILALKIARSIYCPLFQEVASQCNQSRIXGFWRRLTRCAKPSCPGDLIY
EKKGPAFIPSCSNLNPSPFYQELTETCACPKGSVLDNGAIGHHCIPESGCSCEFAGKTYGNGEMRSSKCQSCTCHGGKWRCSENLCHKR
CVIEGQFVTTFDGKQYVLPQKCSYVASQGPSWIIIIHFSRKGLFLRKITVQLSEELFVFKKNEVLLYPDCDCRNGKVCVSCSEDRHERI
QKSCDYISKPRGTSETCKSGCYCPEYQYEDHHGNCVSLDECTCVFSGKTFKAGQQVNSNCKTCTCYQGQWHCIERPCPGQCQVYGNGHY
QTFDSKWFRFSGQCLYTLVQDSCGNGNGTFSIGVESVPCCEEALTCSRNIILDLKGQVSLTLSDMQVTRHLYKGWTGQEDSLYLV
>Pungitius_spgβ
LAFCFSLASVFGTVELFETKETHIYTCRTFGSGVVQPFKGETYYVRSDCPFTLTSFTVKRGEYSVSIRRGHNGLLVQVEIIINKVTTLL
KNGHILVQNKSVSLPYDHTYQHIFKYGIYTRLRSTVLPFTVTWHNVHGGINSMWVTLESKLSADMSGLCGQQKVAAPRHELIGDSKLDD
HRCKTRDPVMQKNHHMCFQFFLDTKDCLQDNNRHYHRLCKENIFGFENSQSIYCPLFQEVASQCSQSSINPFWRRLTRCAKPSCPGHLI
YEKKGPAFIHSCSNPNPSPFYQELTETCACPKGKVLNNGAKGHHCIPKSSCSCEFAGKSYGNGEIRSSKCQSCKCHGVKWRCSENFCKK
RCVIEGQFVTTFDGKQYVLPQKCSYVASQGPNWKIEIHFSRKRLFLRKATVQLSEELFVFKKNKVMWHAQIYWVSSMFVQVHTTSGLNF
QIQLSPEIQLFIGAPDKSKDKIKGLCGNSNSDTTDDFTTNRGIIGNSAKPFALSWSLGNCHENIPTTCTNRENENYAHEKCASVLNHPT
GIFAVCHPHILTDYYYTACIQRICNSGRSRREALCISLASYAKVCAGVGVVI
>Pungitius_spgγ
MTTQRWILAFCFSLASVFGTVELFETKETHIYTCRTFGSGVVQPFKGETYYVRSDCPFTLTSFTVNRGEYSVSIRRGHNGLLVQVEIII
NKVTTLLQNGQILVQNKSVSLPYDHTYQHIFKYGIYTRLRSTVLPFTVTWHSVHGGINSMWVTLESKLSADMSGLCGQQKVAAPRHELI
RDSKLDDHRCKTRDPVMQKNHMCGXFFRKTKNPSCPGDXIYEKKXPAFIPSCSNLNPSPFYQELTETCACPKGSVLDNGAIGHHCIPES
GCSCEFAGKTYGNGEMRSSKCQSCTCHGGKWRCSENLCHKRCVIEGQFVTTFDGKQYVLPQKCSYVASQGPSWIIIIHFSRKGLFLRKI
TVQLSEELFVFKKNEVLLYGHEITEIHQSGHAQIYWVSSMFVQVHTTSGLN
>Tetraodon_spiggin_homolog
MTSQRWMLMLSFSLVVFGLAKIVPTAETHKYTCRTFGSGVIQSFNGSSFYVRSNCPFTLTRFTQNRVECDITTRRDDSGLLVRVEIIIN
KIRTVLQNGSILVEGTSVSLPYDHTYQHIFQYGIYVKLRSSLLPLSVTWHNVPGGLDALLVEVEQEPSSDVAGLCGKPNVTADRQQLTA
QSRIADDTCQTRDPARSDNAVCRTFFSQAMGCLGAGHRLQEYIQLCEENMYTYEANTHIRCAFFREMMQQCPSESPVWDIWRHTTSCQE
PTCPGDLVYEERGPAFPSSCSNPNPAPSNEDHVSSCVCPPNTVLDDHGGGFRCVSERSCPCVFAGKSYSTGDVWTTKCQTCICKSGKWR
CSENFCPSRCVVEGQFVTTIDGKHFDVPGECTYIASQGHNWTVTIEFSKTRSLKTILLHLFQEEYTFSHNLVKIGEDVIGELHTSDHAL
VFWESSMYVQVHTSLGLKIQVQVSPEIQMYLTPPANHMGPISGLCGNSNSDTRDDFTTSSGIIENSAQAFALSWSVGTCAVTPNPCINT
DNEIFAEEKCSILNNPTGIFGKCHAHIPTDHYHRACIQQTCNNGRSLQQSVCVALGNYAKACANQGVFIGDWRTATNCTVKCEKNQEFS
YNTHACNHTCRSLFGSDPRCGLDDAPAEGCGCPEGTHLNLESTCTPKADCFCHYSGGTTPPGPVVIDGRQCLCKDGELRCSKDCGCHTG
KVCVHCAENPVNTARKTCDSLSKPVGVGAACDSGCYCPYNQYEDHLGNCVSVENCTCVYSGRVFSAGQSVKSNCKTCVCGQGQWTCSDQ
PCSGKCQVYGNGHYQTFDSNWYRFSGHCQYTFVE
>Takifugu_spiggin_homolog
MISPRGTLTLCFLLVGTITDFINTCSTFGSGVIQPFNGSSFYVRSNCPFTLTRFTHNRVDCDITMRRGDSGLLVHVEIIINKIRTVLQN
GSILVEGRSVSLPYDHTYQHIFQYGVFTKLRSSLLPLSVTWHNVPGGIDSLRVEVEQQLSGLVTGLCGKHNATAHRQQLVAESTLADDT
CQTRDPAFADNKICRDFFSYTLECLQARTLHYIQLCEENLYTYEANSYIACAFFREVVQQCGNSSHIWNIWRTETSCKEPTCPGDLIYA

EQGPAFAPSCSNPDPAISNQDLVASCVCPKNKLRNDVGGGFGCVSVPSCPCVFAGKSYSTGDTRSTKCQTCMCDSGKWHCSANFCPATC
VIEGHFVTTIDGKRYTVPGKCTYVASQGYNWTVITEFYKATPTLKTVILHLFQEVYTFSHNMVKIGEDKITELHTSDHALVFWESSMYV
QVHTSLGLKIQVQVSPQIQLYLTPPTNHTGPISGLCGNSNSDTRDDFTTSSGIVENSAQPFALSWTLGTCAVNILDTCINTDNEIFAEE
KCSILNDPNGMFAKCHHHIPTDHYHKACIQRTCNCVRRLEECICVALGNYAKACAGLGIFIGDWRKATNCTVKCENNQEFSYNIRACNH
TCRSLSGSDPRCGLDDMPVDGCGCPEGTHLNLEATCTTKADCFCNYYGGTTPPGPVVIDGRQCICEDGELHCSKDCGCRNGKVCVHCAE
FPVNTARKTCDSLSKPVVSVTCDSGCYCPYDQYEDHLGNCVSVENCTCIYSGRVFTAGQSVKSNCKTCICGQGQWSCRDEPCSGKCQVY
GNGHYQTFDSNLYRFDGHCQYTFVEDACGTRNGTFSIRIESVPCCDEALTCSRSVVFDLQGIVTLTLSDMKVTRRHHDGWTLGKDLLYT
VRSVGLYIIVSVPSKGITLIWDKHTRLTVELQPHWRNQVCGLCGNFDASEMNDLQLMIGSPMTFGNSWKATTPPCSDVTTEIFPCERNS
YCSAWAQRRCMILKGDTFKQCHLLVEPEPYYHACVQESCSCEFEGKFLGFCTAVAAYAEACSDQLVCINWRTPDLCR
>Oryzias_spiggin_homolog
CSTFLSPATECLKEKWKPYEDICSKNNYKASKEVKCSFFNEIALQCKDKPYFTKSKLCEKPTCPGELKFNETGSPFAPSCSNPNPSSSE
TVQTCVCRDGKVRNDRVNASQCVRRSDCPCVFAGKIYQPGTSRNTRCQSCSCNGGNWVCSANICPPKCTVEGQFVETFDGKPYTLPKRC
RYVVSKGSNWTIKADFSASEIEVTKVVIELFEETYTFEDKKVKLKEKEITEFHKSDQALVFWQSSMFVLVQTSFDMKIQVQMSPIMQLY
ITLPGNNIETLSGLCGNGNNDTTDDFTSSNNIRESSSGPFALSWAYGLTGGSQCTTEDIPTVCVNSAAEIFAADRCAALINNKVFAECH
SYISPEAYRADCIKTTCTCGSNKEDCVCTALGNYAKACTGLGIKLGDWRSSTNCTISCQTNQVFSYNNVICNSTCSSLSGNDPRCEVKD
DPVEGCGCPEGTHLGDTNVCISKAECPCHYLGGTTPPGQKVIDGLLCNCENGELECSKDCGCTKGKVCYHCSQGWMSTAQKTCESLLK
>Danio_spiggin_homolog
MFNQSIINCLISLLIKQIFSQSISTNQTDQSVTPSDVCRTYGSGVFSSFNGTLFHLKSSCAVTLTHFTHAGADCHVIVQRGTTGLMDRV
EIIVNKITTLVHNNTVTVDGSRISLPYDHTYQHVFRYGIYTRLQSKILPLSVTWYSVPDGVSSLWVSNHLTLSILCTYQVNHLCYKSMN
GLWHHEVNCSFFKEISLMCGSSSPFWSIWRSKTNCCKSHSLKYFLLVCLKCERILFAAMPLCPGDLRYVEMGPAFPPTCSNPQNSTADF
TSTCLPAEGQFFIVGYKCHNLNIFVTVKHWRDLGCAYPHSTCIRGKWACSANTCPVKCIIEGQFITTFDGKQYSLPDRCTYVAARVTLT
IIMSSQMWHFALFYENASFWYSGALDFDCAVFCTNVKYSRLPFSNMYFAFVSESTTVFWQSSMFVQVYTSFGLKMQVQVSPEIQIYLNL
PATENTKGLCGTYNNDTNDDFTTFSGIIESSVQLFAQSWSMGSCTPITGCINTNNGKEIRDLKNRTQRIHKNKNCTRDVFGALQWIGYI
FRMSLGGNKCIPFLPTVLVVCAHIYIQYVSPTHIFWFSVPPCMGNLMFAYETMACNHTCRSLSAPDPTCAVLDDPVEGCGCASDSHLDN
QQTCSPKTLCSCYHPGGVAPPGPVVIGGRQWRLKLCKCYKHYGYYEPWFTFIRVPLAVQTQASELLVPNCTILDVFAFPTTHQFAFYCV
LPLCFSGQCQVYGDPHYISFQGTTFDFMENCTYTLVEEQLVNQRLSITVDNYYCLPEIDDSCSRGITLNYWNDVVTLMVTEEYTVEVCL
YSKQLYDYLCTW
