# Supplementary figures and images for: Extensive lineage-specific gene duplication and evolution of the spiggin multi-gene family in stickleback
Source: BMC Evol Biol. 2007 Nov 4;7:209. doi: 10.1186/1471-2148-7-209 (PMC2180178; doi:10.1186/1471-2148-7-209)

Torafugu

Male

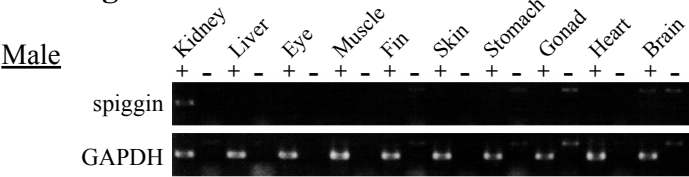

Female

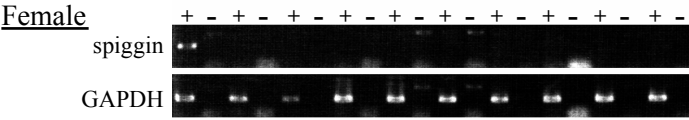

Zebrafish

Male

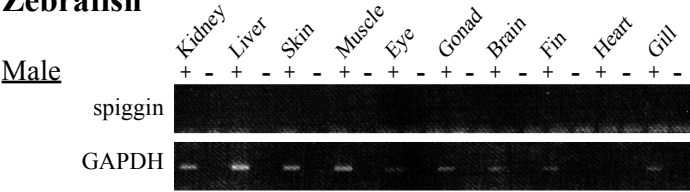

Female

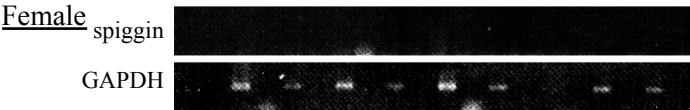

Supplement: Additional file 3 — "Expression analysis of spiggin homologs in torafugu and zebrafish". Expression of spiggin homologs in various tissues of torafugu and zebrafish determined by RT-PCR. Glyceraldehyde-3-phosphate dehydrogenase (GAPDH) was used as a positive control. Plus signs indicate amplification using reverse-transcribed cDNA from each tissue; minus signs indicate negative controls using RNA samples without reverse-transcription. [file 1471-2148-7-209-S3.pdf]

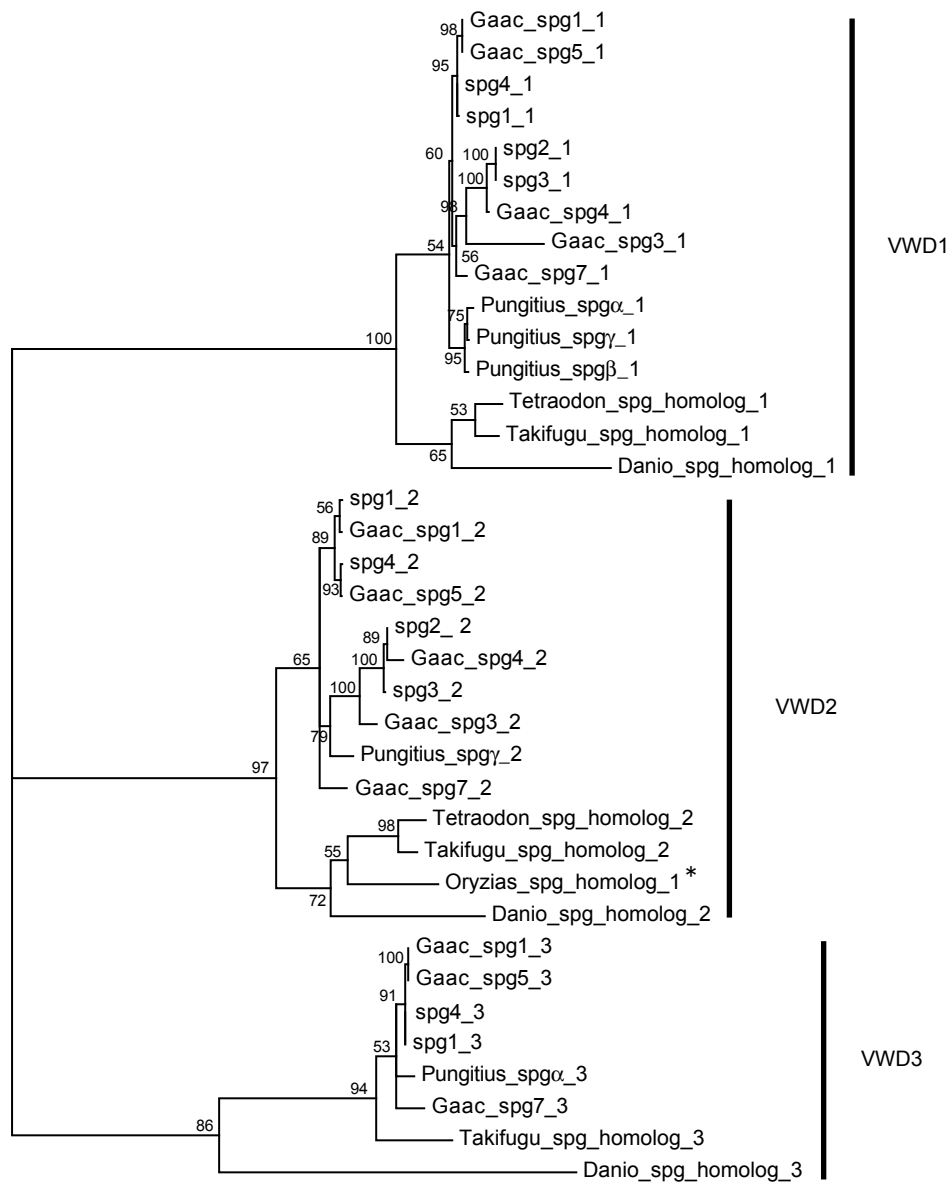

Supplement: Additional file 5 — "Phylogenetic tree using conserved domain structures". Reconstructed phylogenetic tree based on the conserved domain structures (VWD) of spiggins and their homologs. We used translated sequences of threespine and ninespine stickleback spiggin genes that were isolated from the genome sequence (Gaac_spg1, 3, 4, 5, and 7), published spiggin cDNA sequences (spg1-spg4; Pungitius_spgα-γ), and spiggin homologs isolated in torafugu (Takifugu_spg_homolog), spotted green pufferfish (Tetraodon_spg_homolog), medaka (Oryzias_spg_homolog), and zebrafish (Danio_spg_homolog). We extracted conserved domain structures (VWD), numbered them from the N-terminal end, and subjected them to phylogenetic analysis. The ML tree is shown; numbers at nodes in internal branches indicate % bootstrap values (500 replicates). The asterisk indicates the VWD domain of medaka spiggin homolog, for which the relative position is incongruent with those of other domains. [file 1471-2148-7-209-S5.pdf]
