# Supplementary material for: Extensive lineage-specific gene duplication and evolution of the spiggin multi-gene family in stickleback
Source: BMC Evol Biol. 2007 Nov 4;7:209. doi: 10.1186/1471-2148-7-209 (PMC2180178; doi:10.1186/1471-2148-7-209)
Supplement: Additional file 4 — "Primers used". Sequences of primers that were used in this study. [file 1471-2148-7-209-S4.pdf]

Additional file

Primers used in this study

Primer	Sequence (5'- 3') [a]	Source	Purpose of this study
Spg2_ex3_Fw	AGGACATTTGGGAGCGGGATCATCCAGCC	This study	Genomic PCR
Spg2_ex3_Rv	CATTAAATTCTAGTAGTGTAAAATG	This study	Genomic PCR
Spg2_ex16_Fw	CCTGGAATCTACAAAAGGGCCGTAAC	This study	Genomic PCR
Spg2_ex16_Rv	CAACCCTCCACAGGATTGTCGTTC	This study	Genomic PCR
Spg6_ex13_Fw	CAACGTCACGATGTAACAGACACAG	This study	Genomic PCR
Spg6_ex13_Rv	GTCATAAAGGAAGTGATTGCCAGTG	This study	Genomic PCR
Spg6_ex17_Fw	CCCATTTTAATAGAATACATAAATCG	This study	Genomic PCR
Spg6_ex17_Rv	GAGCTTTCCGAAACCCTAACAGAGAC	This study	Genomic PCR
Spg6_ex22_Fw	GTTTGCAATACATCGCCAAATGCTGAAGG	This study	Genomic PCR
Spg6_ex22_Rv	GTAACTGCTGGAGTCTTGATTCTTCCG	This study	Genomic PCR
Fspg_Fw	TACCAGCAYATCTTYCARTAYGG	This study	Expression analysis
Fspg_Rv	CCATCGATGGTGGTNACRAANTG	This study	Expression analysis
Zspg_Fw	CTATCAGCATGTGTTTCGCTACG	This study	Expression analysis
Zspg_Rv	CCATCAAACGTCGTAATGAACTGTCC	This study	Expression analysis
Gapdh_Fw	GCCATCAAYGAYCCYTTCATYGAC	Kawahara and Nishida, 2006	Expression analysis
Gapdh_Rv	TTDCCRTTVAGCTCRGGGATRAC	Kawahara and Nishida, 2006	Expression analysis

[a] IUB codes are used to identify redundancies.
